# Polyoxa- and Polyazamacrocycles Incorporating 6,7-Diaminoquinoxaline Moiety: Synthesis and Application as Tunable Optical pH-Indicators in Aqueous Solution

**DOI:** 10.3390/molecules28020512

**Published:** 2023-01-04

**Authors:** Igor A. Kurashov, Alisa D. Kharlamova, Anton S. Abel, Alexei D. Averin, Irina P. Beletskaya

**Affiliations:** 1 Department of Chemistry, Lomonosov Moscow State University, Leninskie Gory, 1-3, Moscow 119991, Russia; 2 Frumkin Institute of Physical Chemistry and Electrochemistry, Russian Academy of Sciences, Leninsky Pr. 31, Moscow 119071, Russia

**Keywords:** macrocycles, amination, Pd-catalysis, quinoxaline, colorimetry, fluorescence, pH indicator

## Abstract

Synthetic approach to fluorescent polyaza- and polyoxadiazamacrocycles comprising a structural fragment of 6,7-diamino-2,3-diphenylquinoxaline has been elaborated using Pd-catalyzed amination providing target compounds in yields up to 77%. A series of nine novel *N*- and *N*,*O*-containing macrocyclic ligands differing by the number of donor sites and cavity size has been obtained. These compounds possess well-pronounced fluorescent properties with emission maxima in a blue region in aprotic solvents and high quantum yields of fluorescence, while in proton media, fluorescence shifts towards the green region of the spectrum. Using macrocycles **5c** and **5e** as examples, we have shown that such compounds can serve as dual-channel (colorimetric and fluorimetric) pH indicators in water media, with pH transition point and response being dependent on the macrocycle structure due to different sequences of protonation steps.

## 1. Introduction

Elaboration of efficient synthetic approaches to novel macrocyclic compounds of various types (porphyrins, calixarenes, crown ethers, tetraazamacrocycles, cryptands) is of great interest due to their unique properties. They find wide applications in supramolecular chemistry [1,2,3,4,5,6], in the construction of molecular machines [7,8,9], in coordination chemistry [10,11,12], for medical applications [13,14], and as new components of advanced materials [15]. Macrocyclic ligands are used as universal building blocks in the design of receptors [16,17,18] and optical sensors [19,20,21]. Substituted polyazamacrocyclic ligands are widely used as chelators for lanthanide ions for the design of various luminescent and photoactive materials [22,23,24].

**Figure 1:**
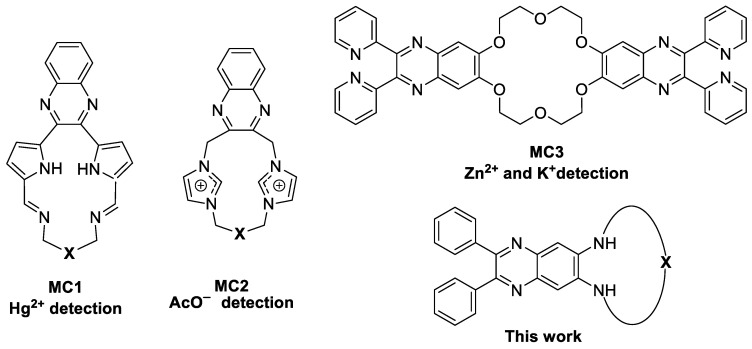
Previously reported macrocyclic chemosensors based on quinoxaline signaling units.

Quinoxalines and their derivatives are important structural fragments of various chemosensors due to their optical properties. They are applied as signaling groups for detecting metal cations [25,26,27,28], anions [29,30,31,32,33] and as pH detectors [34,35,36]; quinoxalines are also used in the fabrication of novel organic light-emitting diodes (OLEDs) [27,37,38]. The ability of quinoxalines to sustain fluorescent properties in the solid phase allows the manufacturing of fluorescent materials using this fluorophore which also can be used for detection needs [39,40]. Wide possibilities of quinoxaline functionalization with electron donor and electron-withdrawing substituents in different positions open the way to the compounds of various architectures like linear molecules, macrocycles, and polymeric chains. A combination of the quinoxaline fragment and macrocyclic receptor unit in several cases was shown to be efficient for the synthesis of sensing molecules. Thus, a series of quinoxaline-containing derivatives of calixarenes [41,42,43] and porphyrinoid-based macrocycles with quinoxaline groups [44] were described as anion-sensing chemosensors. Substituted quinoxalines containing macrocyclic moieties (**MC**) were reported (Figure 1), allowing the detection of Hg(II) cations (**MC1**) [45] and acetate anions (**MC2**) [46]. Tritopic ligand featuring two quinoxaline moieties and annelated 18-crown-6 ether (**MC3**) produces a different response to Zn(II) cations in the presence and in the absence of K^+^ ions serving as a logic gate [47].

Earlier, we demonstrated that Pd-catalyzed amination of halogenoarenes could be used for one-step synthesis of various macrocycles in good yields [48,49,50,51]. Such macrocycles can be further developed into colorimetric [52,53] and fluorescent [54,55,56] chemosensors for metal cations detection, as well as for enantioselective recognition of chiral organic molecules [57,58]. In this work, we propose an approach to macrocyclic ligands on the basis of 6,7-annelated 2,3-diphenylquinoxaline and diazacrown ethers (Figure 1) and show the results of the preliminary studies of their optical and sensing properties.

## 2. Results and Discussion

### 2.1. Synthesis

**Scheme 1:**
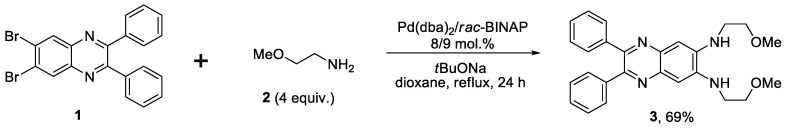
Synthesis of diamine **3** via Pd-catalyzed diamination of dibromoquinoxaline **1** with amine **2**.

An available 6,7-dibromo-2,3-diphenylquinoxaline (**1**) was used as a starting compound. It was obtained by the reaction of benzil with 4,5-dibromo-1,2-diaminobenzene [59]. Possibilities of the Pd-catalyzed diamination of **1** were studied using a model 2-methoxyethylamine (**2**) (Scheme 1).

The reaction was carried out in the presence of the catalytic system Pd(dba)_2_/BINAP (8/9 mol%) (dba—dibenzylideneacetone, BINAP—2,2′-bis(diphenylphosphino)-1,1′-binaphthalene) in dioxane (0.1 M) under reflux for 24 h in the presence of 3 equiv. *t*BuONa as a base. Usually, the Pd-catalyzed diamination of the *ortho*-dihalogenoarenes is hindered because the introduction of the first amino group decreases the reactivity of the resting halogen atom [60,61]. In our case, the application of 4 equiv. 2-methoxyethylamine led to a 69% isolated yield of the target compound **3**. This favorable result can be explained by a higher reactivity of the halogen atoms in the starting compound **1**, an electron-deficient heterocycle.

**Scheme 2:**
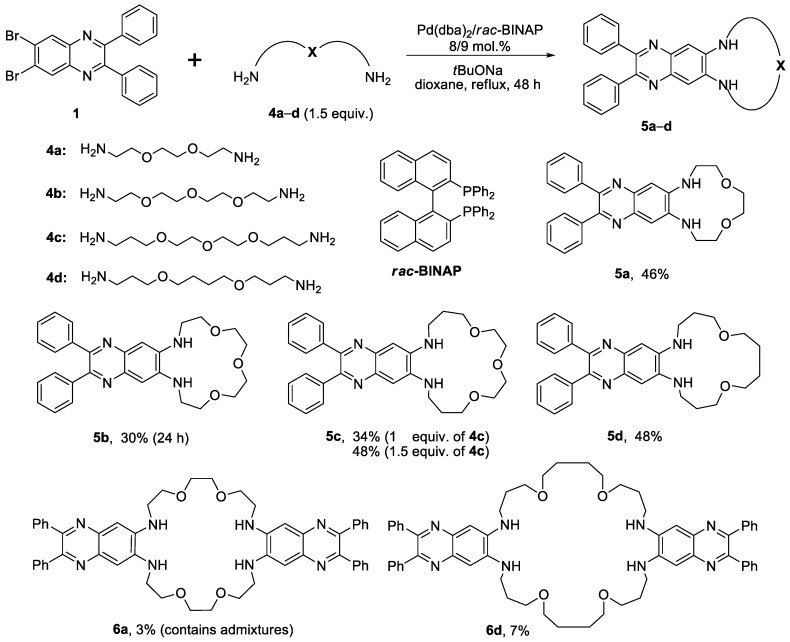
Pd-catalyzed synthesis of oxaazamacrocycles **5a**–**d**.

Encouraged by this result, we studied the possibility of the synthesis of oxaazamacrocycles **5a**–**d** using a series of oxadiamines **4a**–**d** (Scheme 2).

The reaction conditions were adjusted to the synthesis of macrocycles by diminishing the concentration of the reagents to 0.02 M in view of reducing the oligomerization processes. Initial experiments were carried out with oxadiamine **4a**. In the case of applying 1 equiv. of dioxadiamine **4a,** the conversion of the starting dibromoquinoxaline **1** reached only 50%. After the increase in the reaction time up to 48 h, the conversion reached 75%, and the application of 1.5 equiv. dioxadiamine **4a** resulted in full conversion of the starting compound and 46% isolated yield of the target macrocycle **5a**. Cyclic dimer **6a** was also obtained, though in a tiny yield of 3%. Note compound **5a** is a cyclic analog of the diamino derivative **3**, which is important for further coordination studies. In the reaction of trioxadiamine **4b,** a full conversion of **1** was achieved in 24 h, and the yield of the corresponding macrocycle **5b** was 30%. In the case of 1 equiv. of dioxadiamine **4c,** the reaction provided a 34% yield of the macrocycle **5c** and the application of 1.5 equiv. raised the yield to 48%. Compound **5d** was equally obtained in 48% yield under optimized conditions, and the cyclic dimer **6d** was isolated in 7% yield.

**Scheme 3:**
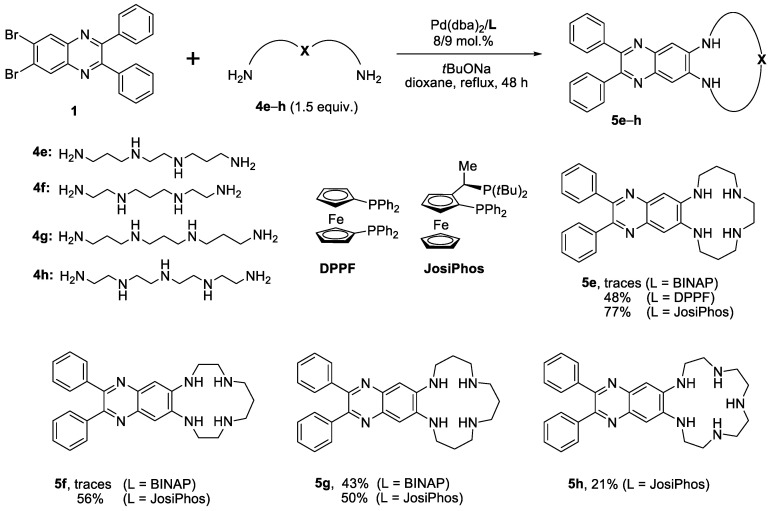
Pd-catalyzed synthesis of polyazamacrocycles **5e–h**.

In order to furnish polyazamacrocyclic compounds, the reactions with a number of linear polyamines **4e**–**h** were carried out under similar conditions (Scheme 3). The much higher reactivity of the primary amino groups than that of the secondary amino groups in the Pd-catalyzed amination reactions is well established and ensures the formation of the corresponding macrocycles [62]. However, polyamines are better chelators for palladium than oxadiamines and can lead to a decrease in the efficiency of the process and diminish the yield of the target products [61].

The reactions conducted with the tetraamine **4e** showed that Pd(dba)_2_/BINAP catalytic system was inefficient even at a higher concentration (0.06 M). Only products of C-Br bond reduction and oligomers were observed in the reaction mixture. The application of the Pd(dba)_2_/DPPF (8/9 mol%, DPPF—1,1′-bis(diphenylphosphino)ferrocene), which previously was shown to be optimal for the diamination of **1** with 1,3-diaminopropane [36], provided the desired macrocycle **5e** in 48% yield. Earlier JosiPhos ligand ((*R*)-1-[(*SP*)-2-(diphenylphosphino)ferrocenyl]ethyldi-*tert*-butylphosphine) and ligands of this type were noted for its good performance in the Pd-catalyzed amination of halogen heteroarenes [63,64], and its application in our case increased the yield of **5e** to 77%. Under analogous conditions, tetraazamacrocycles **5f**,**g** were obtained in 56 and 50% yields, respectively, while the reaction with the pentaamine **4h** was more difficult and afforded **5h** in only 21% yield. The use of a less expensive Pd(dba)_2_/BINAP catalytic system was successful only in the synthesis of the macrocycle **5g** (43%), the result being quite comparable with that obtained with the JosiPhos ligand. The structure of oxadiamines and polyamines strongly governs the catalytic conditions and demands fine-tuning of the reaction conditions in each case. Thus, we obtained a series of nine macrocyclic ligands differing by the number of donor sites and the macrocyclic cavity size.

### 2.2. Optical Properties

Initially, the spectral properties of the obtained macrocycles were studied in acetonitrile solutions, and the main spectral data are given in Table 1. The compounds **3** and **5a**–**h** possess well-pronounced photoluminescent properties, which are characteristic of the electron push-pull systems of quinoxaline [36,65,66]. The absorption spectra possess strong bands at 260–280 nm corresponding to π–π* transitions and broad bands in the 404–410 nm region corresponding to ICT [67]. Measured quantum yields range from 44 to 68% providing high brightness of the compounds.

**Figure 2:**
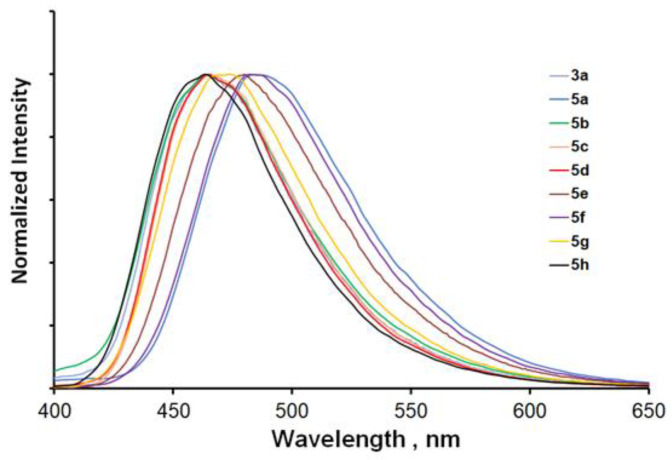
Normalized fluorescence spectra of **3** and **5a–h** (λ_ex_ = 405 nm) in MeCN.

The majority of compounds (**3**, **5b**–**d**, **5g**–**h**) possess emission maxima at 464–466 nm (Figure 2), while some macrocycles with small-size macrocycles (**5a**, **5e**, and **5f**) have emission maxima at a greater wavelength (485, 480, and 486 nm, respectively). It might be explained by the fact that higher conformational rigidity of these macrocycles hinders full conjugation of the amino groups at positions 6 and 7 with the aromatic system. To a certain extent, it can be supported by the values of the chemical shifts of the protons at positions 5 and 8 (Figures S47–S64). In the non-cyclic compound **3**, the chemical shift of these protons is 7.15 ppm. In the macrocycles **5b**–**d,** it is shifted to 7.05–7.09 ppm; in the macrocycle **5e,** these protons lay at 7.01 ppm, while macrocycles **5g**,**h** are characterized by substantially upfield chemical shifts (ca 6.85 ppm). In the macrocycles **5a** and **5f**, with the smallest macro ring sizes, these protons are downshifted to 7.27–7.29 ppm. Thus, the rigidity of the macrocyclic ring influences spectral properties of the compounds. Analogous interdependence of the chemical shifts, size of the macrocyclic ring and photophysical data was observed by us earlier for the quinoline-containing macrocycles [55].

**Figure 3:**
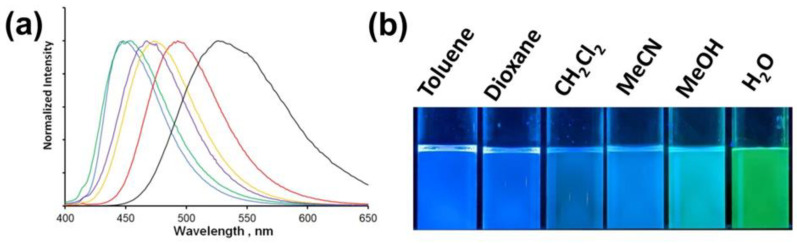
(**a**) Normalized fluorescence spectra of **5e** (λ_ex_ = 400 nm) in toluene (blue), dioxane (green), CH_2_Cl_2_ (violet), MeCN (yellow), MeOH (red), water (1 vol% MeOH, pH = 6, black); (**b**). Color evolution of solutions of ligand **5e** observed in various solvents under UV light (λ = 365 nm).

The influence of the solvent polarity on the optical properties of the compounds was studied using exemplary **3**, **5c** and **5e**. Absorption and fluorescence spectra were registered in toluene, dioxane, dichloromethane, acetonitrile and methanol. The data are presented in Table S1. The dependence of solvent polarity on the fluorescence of the ICT systems is well established [69]. The excited state of the fluorophore is characterized by high polarity because of charge separation, and this leads to a strong interaction with the polar solvent. This interaction energy strongly depends on the polarity of the solvent and influences the energy gap between the ground and excited states. The polar solvents cause a diminishing of the gap. In our case, the increase in the solvent polarity leads to a bathochromic shift of the emission maxima of compounds **3** and **5c**: in toluene and dioxane it lies at 450 nm, in dichloromethane and acetonitrile at 462–465 nm, more polar methanol shifts it to the 482–485 nm region. Compound **5e** features a similar tendency, but in polar solvents, emission maxima are more red-shifted compared to **3** and **5c** (Figure 3). The influence of the solvent on the quantum yields is indefinite. It may be due to various specific interactions of the molecules of the solvent with the coordination sites of the compounds tested, leading to non-emissive relaxations of various intensities.

In order to study the spectral properties of **3**, **5c** and **5e** in water, their solubility was preliminarily investigated. The first two compounds are insoluble in water; thus, we used a 1 vol% solution of MeOH. In the 0.5–8 µM range, the Beer–Lambert law was shown to be observed (Figures S7 and S10). For A_408_ < 0.1 (Figures S8 and S10). Polyazamacrocycle **5e** is sufficiently soluble in water without methanol additive due to partial protonation of the dialkylamino groups, and the Beer–Lambert law is observed for a wider range of concentrations, likely it was the case with a non-cyclic analog [36]. Compounds **3**, **5c** and **5e** possess fluorescence in the green region, their emission maxima being 507, 510 and 526 nm, respectively (Table S1). Water is considered to be one of the most polar solvents (1.3 times more polar than methanol), which leads to such a strong shift in the emission maximum. As expected, quantum yields in water are lower than in organic solutions (27, 34 and 36%, respectively). However, they still provide enough high emission intensity, which can be observed by a naked eye (Figure 3), which is important for the construction of sensor devices.

### 2.3. Detection of Metal Cations in Acetonitrile

Compounds **3** and **5a**–**d** were evaluated for their possibilities of metal detection in acetonitrile. Absorption and fluorescence spectra of these compounds were registered in the presence of 18 metal perchlorates (Figures S13, S17, S21, S25, S29). Every *N,O*-ligand showed almost full emission quenching in the presence of such cations as Cu(II), Hg(II), Al(III). Partial quenching of fluorescence was noted in the presence of Pb(II) and Zn(II). A bathochromic shift of the absorption band corresponding to ICT was also observed. The lack of selectivity allows only for the use of these ligands as fluorescent molecular probes for the mentioned cations.

**Figure 4:**
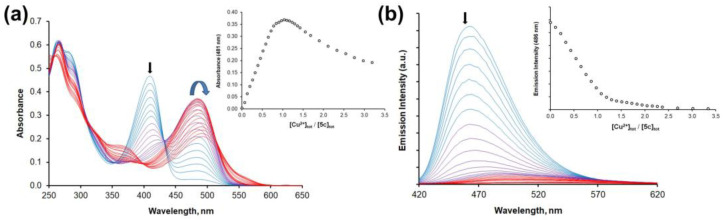
(**a**) Evolution of the UV-vis absorption spectrum of **5c** in acetonitrile ([**5c**] = 24.4 μM) upon addition of Cu(ClO_4_)_2_ (0–3.3 equiv.). Inset: changes of the absorbance as a function of the [Cu^2+^]_tot_/[**5c**]_tot_ ratio at λ = 481 nm. (**b**) Evolution of the emission spectrum of **5c** in acetonitrile ([**5c**]_tot_ = 4.2 μM, λ_ex_ = 409 nm) upon addition of Cu(ClO_4_)_2_ (0–3.3 equiv.). Inset: changes of the emission intensity as a function of the [Cu^2+^]_tot_/[**5c**]_tot_ ratio at λ_em_ = 486 nm. The arrows show the change of the spectra.

More detailed investigations were carried out with Cu(II) salt in order to elucidate the influence of the nature of the receptor unit on the composition and stability of complexes. For this purpose, spectrophotometric and spectrofluorimetric titrations were fulfilled (Figures S14–S16, S18–S20, S22–S24, S26–28 and S30–32). As an example, Figure 4 depicts the changes in the absorption and fluorescence spectra of the macrocycle **5c** in the presence of different amounts of Cu(II). Detection limits (LODs) determined for **3** and **5a**–**d** using fluorescence spectroscopy (3σ-method) do not substantially depend on the nature of the ligand and are in the range of 4–5 µM.

At the first stage of the titration experiments, as it was expected, each ligand showed the formation of the complex of 1:1 stoichiometry with the bathochromic shift of the absorption maximum and fluorescence quenching. Further addition of copper salt led to a gradual decrease in the intensity of the red-shifted absorption band, indicating the formation of the complexes with another stoichiometry.

Using the HypSpec program, spectral data for spectrophotometric titrations were subjected to the factor analysis, which provided the composition and binding constants of the complexes, which are collected in Table 2. In the case of fluorescent titrations, the formation of 1:1 complexes can be observed, but as all forming complexes are non-emissive, it is impossible to reliably identify them using this kind of titration. All *N,O*-ligands form [Cu**L**]^2+^ complexes of medium stability, the most stable complex (logβ = 7.57(7)) was observed for a non-cyclic ligand **3**, possibly due to conformational lability of its receptor part. Oxygen atoms and nitrogen atoms of the heteroaromatic moiety probably participate in the formation of such complexes.

Macrocycles **5a** and **5b** both form the complexes [Cu**L**]^2+^ and [Cu_4_(**L**)_3_]^8+^, their stabilities being similar, independent of the fact that these macrocycles differ by the cavity size and the number of oxygen atoms. As macrocycles with enough short linkers cannot form polynuclear complexes, the formation of [Cu_4_(**L**)_3_]^8+^ could be explained by the association of three [Cu**L**]^2+^ complexes around one copper(II) center via coordination employing heterocyclic nitrogen atoms. As macrocycles **5c** and **5d** possess larger cavities with longer polymethylene chains between atoms, they can promote the formation of the complexes of another composition. Indeed, with these two macrocyclic ligands, besides [Cu**L**]^2+^, complexes featuring two macrocycles and four Cu(II) cations [Cu_4_(**L**)_2_]^8+^ were observed. Thus, the size and flexibility of the macrocyclic cavity influence stability and composition of the complexes. The studied *N,O*-ligands can form mono- and polynuclear copper complexes.

### 2.4. Protonation Studies

**Figure 5:**
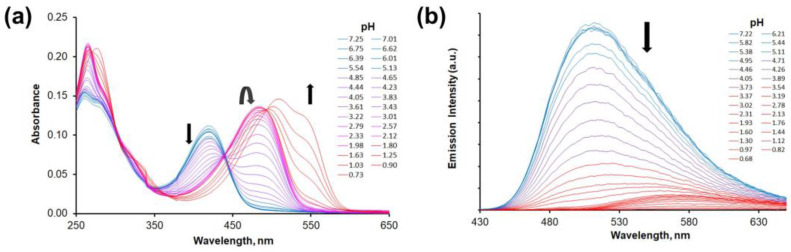
(**a**) Spectrophotometric titration of **5c** as a function of pH ([**5c**]_tot_ = 8.4 μM, 1 vol% MeOH,*I* = 0.1 M NaClO_4_, pH = 0.7–7.2); (**b**) Fluorimetric titration of **5c** as a function of pH ([**5c**]_tot_ = 4.2 μM, 0.5 vol% MeOH, *I* = 0.1 M NaClO_4_, *λ*_ex_ = 420 nm, pH = 0.7–7.2). The arrows show the change of the spectra.

The protonation of the macrocycles in the aqueous media was studied using oxygen-containing compound **5c** and tetraazamacrocycle **5e**. Quinoxaline derivatives were described as convenient platforms for the development of pH sensors [34,35]. As macrocycle **5c** is insoluble in pure water, 0.5–1 vol% methanol solutions in water were applied, which allowed the concentrations of **5c** up to 8 µM. Upon the addition of the acid to an aqueous solution of **5c** notable changes both in absorption and fluorescence spectra take place, like the bathochromic shift of the absorption maximum from 420 nm to 480 nm and partial fluorescent quenching with the shift of the emission maximum from 510 to 570 nm (Figure 5).

**Figure 6:**
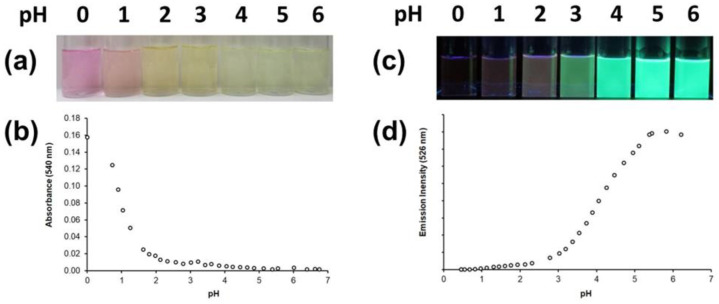
(**a**) Evolution of color of aqueous solution of ligand **5c** with pH increase observed under visible light; (**b**) Absorbance changes with pH at λ = 480 nm for **5c**; (**c**) Evolution of color of aqueous solution of ligand **5c** with pH increase observed under UV light (λ = 365 nm), and (**d**) Emission intensity changes with pH at λ = 480 nm for **5c** (λ_ex_ = 420 nm).

Further diminishing pH value gives rise to a novel absorption band centered at 536 nm resulting in a rose color of the solution visible by a naked eye (Figure 6). Thus, macrocycle **5c** can be proposed as a dual-channel pH sensor providing monitoring of the acidity of the media using both absorption and emission spectra.

**Figure 7:**
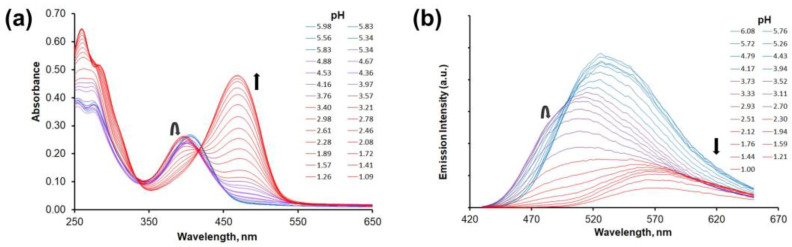
(**a**) Spectrophotometric titration of **5e** ([**5e**]_tot_ = 31.1 μM, *I* = 0.1 M NaClO_4_, pH = 1.0–6.0); (**b**) Fluorimetric titration of **5e** as a function of pH ([**5e**]_tot_ = 8.04 μM, *I* = 0.1 M NaClO_4_, *λ*_ex_ = 420 nm, pH = 1.0–6.0). The arrows show the change of the spectra.

**Figure 8:**
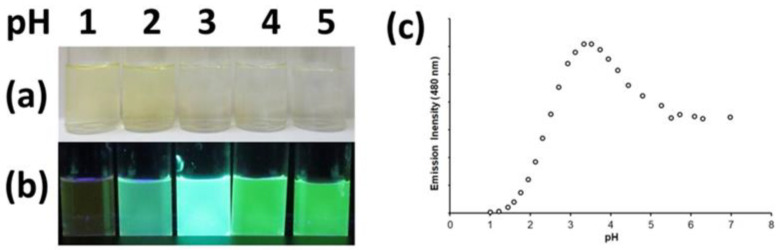
Color evolution of aqueous solution of ligand **5e** with pH increase observed under visible (**a**) and UV (λ = 365 nm) (**b**) light; (**c**) Emission intensity changes with pH at λ = 480 nm for **5e** (λ_ex_ = 420 nm).

A different response to the change of pH is manifested by the tetraazamacrocycle **5e** (Figure 7). A decrease in pH leads to a small hypsochromic shift in the absorption spectrum (from 407 to 395 nm) which is accompanied by a partial emission quenching with a simultaneous shift of the maximum from 528 to 502 nm. A naked-eye note of the change of the fluorescence color from green to blue is possible (Figure 8).

Further addition of acid leads to more pronounced emission quenching with a bathochromic shift of the maximum from 502 to 575 nm, also visible by a naked eye, which coincides with the bathochromic shift of the absorption band from 395 to 475 nm. It was impossible to increase pH above 8.5 due to the formation of the precipitate (Figures S45, S46), which can be explained by the low solubility of non-protonated **5e**. Thus, this macrocycle also can be put forward as a pH sensor like compound **5c**, but the transition points and color responses of these two compounds are different.

The acquired data of spectrophotometric and fluorimetric titrations for the macrocycles **5c** and **5e** were processed with the HypSpec program. Calculated absorption and emission spectra for all species present in the solution and speciation diagrams are given in Figures S33–S44. Protonation constants compared with the known compounds **7** [36] and **8**–**9** [70] (Figure 9) are collected in Table 3.

Earlier, our colleagues studied in detail the protonation of a linear quinoxaline-containing tetraamine **7** using NMR spectra and DFT calculations [36] which provided us with the grounds to put forward the protonation hypothesis for our macrocycles.

**Scheme 4:**
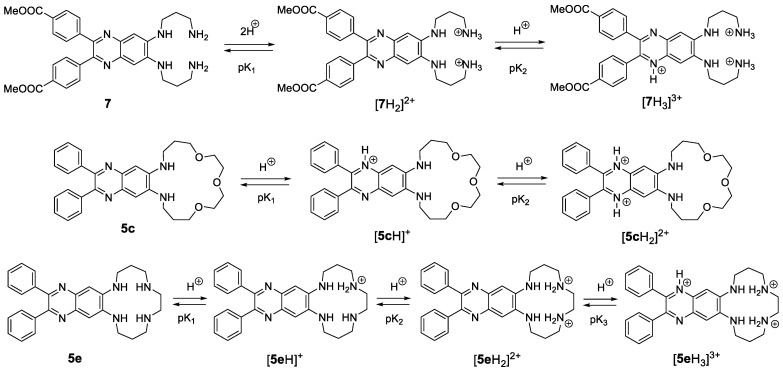
Proposed protonation sequences for quinoxalines **7** [36], **5c** and **5e**.

The proposed protonation sequence is given in Scheme 4. It is assumed that the dialkylamino groups which are not conjugated with the aromatic system are first protonated, and their protonation leads but to a small hypsochromic shift of the ICT band. Such response was noted for 6,7-diaminoquinoxaline [36] and for 6-aminoquinoline derivatives [71]. In a more acidic media, the protonation of the nitrogen atoms of 6,7-diaminoquinoxaline takes place, and the bathochromic shift of the ICT band in absorption and emission spectra is observed. This assumption is in agreement with the calculations provided in [36].

In the case of the macrocycle **5c**, the molecule does not contain amino groups which are not conjugated with the aromatic system, and the protonation of the nitrogen atom of the diaminoquinoxaline fragment takes place accompanied by a bathochromic shift of the ICT band in the absorption and emission spectra. The calculated pK_1_ constant equals 4.10(5) and 3.96(5) (from UV and fluorescence titrations, respectively). These values are in agreement with the protonation constants of the aminoquinoxalines reported earlier [72] but are higher than those reported for compound **7** (2.61 and 3.07). It can be explained by the presence of the protonated dialkyl amino groups and electron-withdrawing COOMe groups in compound **7**. The second protonation step is associated with pK values of 0.6(1) and 1.20(5) (from UV and fluorescence titrations, respectively), which corresponds to the protonation of the second aromatic nitrogen atom. A notable difference between the two values calculated from spectrophotometric and spectrofluorimetric titrations stems from the difference in the basicity of the molecule in the ground and excited states [73].

**Figure 9:**
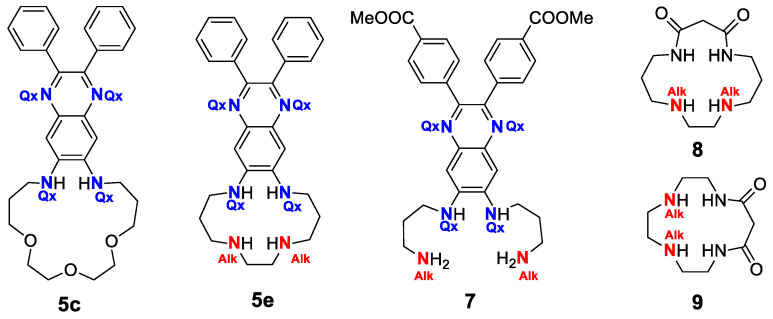
Labeling scheme for nitrogen atoms in chemosensors **5a** and **5e** and relevant compounds **7**–**9** reported in the literature.

Macrocycle **5e** possesses two dialkylamino groups. One of them obviously is protonated at the first step. It seems reasonable to compare this macrocycle with the non-cyclic analog **7**. It is known that the conformational effects in the macrocyclic systems lead to a wide variation in the basicity constants of polyazamacrocycles [70,74,75]. Unfortunately, there are no data in the literature on the protonation constants of benzocyclam. Therefore, for comparison, we selected tetraazamacrocycles **8** and **9**, which are the closest in their structures and in which two neighboring dialkylamino groups connected by an ethylene linker undergo protonation (Figure 9). An attempt to accurately measure the pK_1_ value for compound **5e** failed because when pH 8.5 was reached, precipitation was observed, and the fluorescence of the solution disappeared (Figures S45, S46). This observation confirms the fact that enough good solubility of **5e** in water is due to its protonation. The curve depicting the changes in absorption and fluorescence spectra allows only an approximate estimate of the pK_1_ value ca 9.5, which is consistent with the data for similar macrocycles **8** and **9** [70]. In the molecule of compound **7**, two alkylamino groups are distant from each other, as a result, their protonation occurs independently and the values of the amino group protonation constants do not differ (Table 3). In macrocyclic molecules, due to electrostatic repulsion, the protonation constant of the second nitrogen atom is lower than the first one by several orders of magnitude, and this difference strongly depends on the flexibility of the macrocycle [74]. In the case of compound **5e** spectrophotometric and spectrofluorimetric studies upon the addition of acid gave the values for pK_2_ 3.69(5) and 4.01(5), respectively. These values correspond to the analogous protonation constant of compound **9**. A small hypsochromic shift of the ICT band and the blue shift of the emission maximum observed in the spectrum are consistent with the hypothesis that this constant corresponds to the protonation of the alkylamino group, which is not conjugated with the heterocyclic system. At the next step, the nitrogen atom of the heterocyclic nucleus is protonated, which is accompanied by a characteristic bathochromic shift of the ICT band in the absorption spectrum. A similar situation was described for molecule **7 [36]**. The pK_3_ values obtained from UV and fluorescence spectroscopy data equal 2.36(5) and 2.46(5), respectively, and are also close to those for molecule **7**. In the [**5e**H_2_]^2**+**^ structure, charged protonated aliphatic nitrogen atoms are positioned inevitably closer to the heterocyclic core due to the macrocyclic structure, which leads to lower pK_3_ **5e** values compared to **7**. Upon further addition of acid new changes are observed in the fluorescence spectrum, which allows us to estimate the value of pK_4_ 1.0 (Figure S43). However, in the absorption spectrum, the appearance of the next bathochromic shift (similar to that noted for compound **5c**) is observed only in strongly acidic media (pH < 0), which can be explained by a notable difference in the basicity of the [**5e**H_3_]^3+^ particle in the ground and excited states.

## 3. Materials and Methods

### 3.1. Reagents

Unless otherwise noted, all chemicals and starting materials were obtained commercially from ABCR (Karlsruhe, Germany) and Sigma-Aldrich (Merck Co., Rahway, NJ, USA) and used without further purification. Preparative column chromatography was carried out using Silica gel 60 (40–63 µm) from Merck Co. Dioxane was distilled successively over NaOH and sodium under argon, CH_2_Cl_2_ and CH_3_CN were distilled over CaH_2_, chloroform was distilled over P_2_O_5_, MeOH was used freshly distilled. 4,5-Dibromobenzene-1,2-diamine was obtained according to the described 3-step procedure [76]. Pd(dba)_2_ was synthesized according to a known method [77] and used without recrystallization. *N^1^*-(2-aminoethyl)-*N^2^*-(2-(2-aminoethylamino)ethyl)ethane-1,2-diamine (**4h**) was prepared by treatment of the corresponding pentahydrochloride with a 2.5 M solution of KOH in methanol.

### 3.2. Apparatus

The ^1^H and ^13^C NMR spectra were registered with a Bruker Avance-400 spectrometer (Bruker Daltonics, Bremen, Germany) in chloroform-*d1* or methanol-*d4*, using the residual signals of chloroform or CHD_2_OD as internal standards. MALDI-TOF mass-spectra were registered on a Bruker Daltonics Autoflex II mass-spectrometer (Bruker Daltonics, Bremen, Germany) in positive ion mode with a dithranol matrix and polyethyleneglycols as internal standards. FTIR spectra were registered on a Nicolet iS 5 (Thermo Fisher Scientific, Waltham, MA, USA) with iD3 ATR accessory (ZnSe).

The pH measurements were carried out using the «Mettler Toledo» apparatus (Greifensee, Switzerland) with a combined electrode LE438 in a glass cell. The electrode was calibrated with commercial buffers (pH = 4.01 and 7.00). UV-vis spectra were registered with a Hitachi U-2900 device (Tokyo, Japan) in a quartz cuvette (Hellma, *l* = 1 cm). Fluorescence spectra were registered with Horiba Jobin Yvon Fluoromax-2 apparatus (Edison, NJ, USA) in a quartz cuvette (Hellma, *l*= 1 cm). Luminescence quantum yields were determined relative to quinine sulfate solution in 0.05 H_2_SO_4_ (Φ = 0.53(2)) according to a standard procedure [78].

### 3.3. Synthesis

*6,7-Dibromo-2,3-diphenilquinoxaline,* [59] 4,5-Dibromobenzene-1,2-diamine (2.55 g, 9.6 mmol), benzyl (1.98 g, 9.4 mmol), and isopropanol (18.8 mL) were placed in a flask equipped with a condenser and a magnetic stirrer. The mixture was refluxed for 3 h. The mixture was allowed to cool to room temperature. The brown precipitate was separated on a glass filter. Yield 3.4 g (83%), brown powder, m.p. 166 °C. ^1^H-NMR (400 MHz, CDCl_3_) δ 7.35 (t, 4H, *^3^J* = 7.3 Hz, H(Ph)), 7.40 (t, 2H, *^3^J* = 7.0 Hz, H(Ph)), 7.49 (d, 4H, *^3^J* = 6.7 Hz, H(Ph)), 8.48 (s, H(Qx)). ^13^C-NMR (100.6 MHz, CDCl3) δ 126.5 (2C, C-Br), 128.3 (4C, CH(Ph)), 129.3 (2C, CH(Ph)), 129.8 (4C, CH(Ph)), 133.1 (2C, CH (Qx)), 138.2 (2C, C_quat_(Ph)), 140.3 (2C, C_quat_(Ph)), 154.5 (2C, C_quat_(Ph)). HRMS (MALDI-TOF): *m*/*z* [M + H]^+^ calcd for C_20_H_12_N_2_Br_2_: 438.944; found: 438.946. 

*Palladium-Catalyzed Amination of 6,7-dibromo-2,3-diphenilquinoxaline—General Procedure.* A two-neck flask equipped with a condenser and a magnetic stirrer, flushed with dry argon, was charged with corresponding 6,7-dibromo-2,3-diphenilquinoxaline (132 mg, 0.3 mmol), Pd(dba)_2_ (14 mg, 8 mol%), phosphine ligand (9 mol%), and absolute dioxane (3–15 mL). The mixture was stirred for 2–3 min, then corresponding amine (0.45–1.2 mmol) and *t*BuONa (87 mg, 0.9 mmol) were added, and the reaction mixture was refluxed for 24–48 h. After cooling it down to ambient temperature, the reaction mixture was diluted with CH_2_Cl_2_, the solution filtered and evaporated in vacuo, and the residue was chromatographed on silica gel using a sequence of eluents: CH_2_Cl_2_, CH_2_Cl_2_/MeOH 200:1–3:1, CH_2_Cl_2_/MeOH/NH_3aq_ 100:20:1–100:30:5. In the cases of the compounds **5a** and **5b**, the residue before column chromatography was dissolved in CH_2_Cl_2_ (20 mL) and washed with deionized water (2 × 20 mL) to eliminate sodium salts. The organic phase was dried over 3 Å sieves and concentrated in vacuo, and only then subjected to column chromatography according to the general procedure.

*N,N`-bis(2-methoxyethyl)-2,3-diphenylquinoxaline-6,7-diamine* (**3**) obtained according to General Procedure from 6,7-dibromo-2,3-diphenilquinoxaline (132 mg), amine **2** (90 mg) in the presence of Pd(dba)_2_ (14 mg), BINAP (17 mg) and *t*BuONa (87 mg) in dioxane (3 mL) in 24 h. Eluent CH_2_Cl_2_/MeOH 100:1. Yield 76 mg (59%), yellowish oil. ^1^H-NMR (400 MHz, CDCl_3_) δ_H_ 3.39 (s, 6H, CH_3_), 3.44 (q, 4H, ^3^*J* = 5.2 Hz, CH_2_(NH)), 3.71 (t, 4H, ^3^*J* = 5.1 Hz, CH_2_(OMe)), 4.39 (t, 2H, ^3^*J* = 5.2 Hz, NH), 7.15 (s, 2H, H5, H8 (Qx)), 7.27–7.29 (m, 6H, H3, H4, H5 (Ph)), 7.46–7.49 (m, 4H, H2, H6 (Ph)). ^13^C-NMR (100.6 MHz, CDCl_3_) δ_C_ 43.5 (2C, CH_2_NH), 58.7 (2C, OCH_2_), 70.2 (2C, MeO), 104.9 (2C, C5, C8 (Qx)), 127.7 (2C, (Ph)), 128.0 (4C, (Ph)), 129.7 (4C, (Ph)), 138.4 (2C, C4a, C8a (Qx)), 140.0 (2C, C_quat_ (Ph)), 141.8 (2C, C6, C7 (Qx)), 149.0 (2C, C2, C3 (Qx)). IR (neat): 612 (vw), 624 (vw), 641 (vw), 668 (w), 698 (s), 750 (Vs), 759 (s), 765 (vs), 806 (vw), 832 (w), 853 (w), 917 (vw), 978 (w), 1001 (w), 1024 (m), 1065 (m), 1079 (m), 1117 (m), 1157 (w), 1199 (s), 1240 (m), 1261 (m), 1267 (m), 1274 (m), 1313 (vw), 1354 (m), 1391 (vw), 1447 (m), 1482 (m), 1495 (m), 1518 (m), 1573 (w), 1599 (w), 1615 (w), 1719 (vw), 2816 (w), 2829 (w), 2876 (w), 2885 (w), 2926 (w), 2980 (w), 3054 (vw), 3084 (vw), 3371 (w), 3382 (w) cm^−1^. HRMS (MALDI-TOF): *m*/*z* [M + H]^+^ calcd for C_26_H_28_N_4_O_2_^+^: 429.2285; found: 429.2266.

*2,3-Diphenyl-6,7,8,10,11,13,14,15-octahydro-[1,4]dioxa[7,10]diazacyclododecino[8,9-g]quinoxaline* (**5a**) obtained according to General Procedure from 6,7-dibromo-2,3-diphenilquinoxaline (132 mg), amine **4a** (67 mg) in the presence of Pd(dba)_2_ (14 mg), BINAP (17 mg) and *t*BuONa (87 mg) in dioxane (15 mL) in 48 h. Eluent CH_2_Cl_2_/MeOH 100:1. Yield 59 mg (46%), yellowish oil. ^1^H-NMR (400 MHz, CDCl_3_) δ_H_ 3.41 (t, 4H, ^3^*J* = 4.5 Hz, CH_2_(NH)), 3.70 (s, 4H, CH_2_(O)), 3.74 (t, 4H, ^3^*J* = 4.6 Hz, CH_2_(O)) 4.96 (br.s., 2H, NH), 7.27-7.30 (m, 8H, H5, H8 (Qx), H3, H4, H5 (Ph)), 7.45-7.47 (m, 4H, H2, H6 (Qx)). ^13^C-NMR (100.6 MHz, CDCl_3_) δ_C_ 45.7 (2C, CH_2_NH), 69.1 (2C, CH_3_O), 69.8 (2C, OCH_2_), 110.8 (2C, C5, C8 (Qx)), 127.9 (2C, C4 (Ph)), 128.0 (4C, (Ph)), 129.7 (4C, (Ph)), 138.8 (2C, C4a, C8a (Qx)), 139.9 (2C, C_quat_ (Ph)), 143.6 (2C, C6, C7 (Qx)), 149.7 (2C, C2, C3 (Qx)). IR (neat): 612 (w), 614 (w), 624 (w), 668 (w), 675 (m), 694 (s), 698 (vs), 712 (w), 749 (s), 759 (s), 763 (s), 790 (w), 805 (w), 830 (m), 847 (w), 857 (w), 868 (w), 887 (w), 928 (w), 945 (w),977 (m), 1001 (w), 1015 (m), 1023 (m), 1033 (m), 1054 (m), 1061 (m), 1071 (m), 1079 (m), 1106 (m), 1116 (m), 1128 (m), 1149 (w), 1158 (w), 1196 (m), 1226 (m), 1264 (m), 1292 (w), 1309 (w), 1339 (m), 1348 (w), 1369 (w), 1407 (w), 1455 (m), 1474 (m), 1493 (w), 1520 (m), 1565 (w), 1598 (w), 1618 (w), 2851 (w), 2880 (w), 2914 (w), 2952 (w), 3051 (vw), 3317 (w) cm^−1^. HRMS (MALDI-TOF): *m*/*z* [M + K–CH_2_CH_2_]^+^ calcd for C_24_H_22_N_4_O_2_K^+^: 437.1374; found: 437.1405.

*2,3,18,19-Tetraphenyl-6,7,8,10,11,13,14,15,22,23,24,26,27,29,30,31-hexadecahydro-[1,4,13,16]tetraoxa[7,10,19,22]tetraazacyclotetracosino[8,9-g:20,21-g’]diquinoxaline* (**6a**) obtained in mixture with as a by-product in the synthesis of **5a**. Eluent CH_2_Cl_2_/MeOH 50:1. Yield 4 mg (3%), yellow-brown oil. ^1^H-NMR (400 MHz, CDCl_3_) δ_H_ 3.47–3.49 (m, 8H, CH_2_(NH)), 3.72 (s, 8H, CH_2_(O)), 3.90 (t, 8H, *^3^J* = 4.4 Hz, CH_2_(O)) 7.12 (s, 4H, H5, H8 (Qx)), 7.27–7.31 (m, 12H, H3, H4, H5 (Ph)), 7.45–7.47 (m, 8H, H2, H6 (Qx)). NH-protons were not unambiguously assigned due to line broadening. HRMS (MALDI-TOF): *m*/*z* [M + Na]^+^ calcd for C_52_H_52_N_8_O_4_Na^+^: 875.4004; found: 875.3983.

*2,3-Diphenyl-6,7,8,10,11,13,14,16,17,18-decahydro-[1,4,7]trioxa[10,13]diazacyclopentadecino [11,12-g]quinoxaline* (**5b**) obtained according to General Procedure from 6,7-dibromo-2,3-diphenilquinoxaline (132 mg), amine **4b** (87 mg) in the presence of Pd(dba)_2_ (14 mg), BINAP (17 mg) and *t*BuONa (87 mg) in dioxane (15 mL) in 24 h. Eluent CH_2_Cl_2_/MeOH 100:1. Yield 43 mg (30%), yellowish oil. ^1^H-NMR (400 MHz, CDCl_3_) δ_H_ 3.42–3.46 (m, 4H, CH_2_(NH)), 3.66–3.67 (m, 4H, CH_2_(O)), 3.76–3.78 (m, 4H, CH_2_(O)), 3.89 (t, 4H, ^3^*J* = 5.0 Hz, CH_2_(O)), 4.82 (br.s, 2H, NH), 7.09 (s, 2H, H5, H8 (Qx)), 7.27–7.29 (m, 6H, H3, H4, H5 (Ph)), 7.46–7.49 (m, 4H, H2, H6 (Ph)). ^13^C-NMR (100.6 MHz, CDCl_3_) δ_C_ 42.9 (2C, CH_2_NH), 68.1 (2C, OCH_2_) 70.0 (2C, OCH_2_), 70.2 (2C, OCH_2_), 103.7 (2C, C5, C8 (Qx)), 127.7 (2C, C4 (Ph)), 128.0 (4C, (Ph)), 129.9 (4C, (Ph)), 138.5 (2C, C6, C7 (Qx)), 140.0 (2C, C_quat_ (Ph)), 141.5 (2C, C4a, C8a (Qx)), 148.4 (2C, C2, C3 (Qx)). IR (neat): 609 (w), 668 (vw), 699 (s), 733 (m), 768 (m), 792 (vw), 805 (vw), 844 (m), 882 (vw), 919 (w), 943 (w), 979 (w), 1001 (w), 1025 (m), 1074 (s), 1096 (s), 1110 (s), 1130 (vs), 1202 (s), 1226 (s), 1246 (s), 1265 (m), 1354 (s), 1402 (w), 1417 (w), 1455 (s), 1495 (s),1528 (s), 1577 (w), 1618 (m), 1725 (w), 2867 (m), 2906 (m), 3058 (w), 3077 (w), 3365 (m) cm^−1^. HRMS (MALDI-TOF): *m*/*z* [M + K–CH_2_CH_2_]^+^ calcd for C_26_H_26_N_4_O_3_K^+^: 481.1636; found: 481.1605.

*2,3-Diphenyl-6,7,8,9,11,12,14,15,17,18,19,20-dodecahydro-[1,4,7]trioxa[11,14]diazacyclohepta decino[12,13-g]quinoxaline* (**5c**) obtained according to General Procedure from 6,7-dibromo-2,3-diphenilquinoxaline (132 mg), amine **4c** (99 mg) in the presence of Pd(dba)_2_ (14 mg), BINAP (17 mg) and *t*BuONa (87 mg) in dioxane (15 mL) in 48 h. Eluent CH_2_Cl_2_/MeOH 200:1. Yield 72 mg (48%), yellowish oil. ^1^H-NMR (400 MHz, CDCl_3_) δ_H_ 2.06 (quint, 4H, ^3^*J* = 5.3 Hz, CH_2_), 3.50 (q, 4H, ^3^*J* = 5.1 Hz, CH_2_(NH)), 3.68–3.69 (m, 4H, CH_2_(O)), 3.71–3.74 (m, 8H, CH_2_(O)), 4.86 (br. t, 2H, ^3^*J* = 4.9 Hz, NH), 7.07 (s, 2H, H5, H8 (Qx)), 7.27–7.28 (m, 6H, H3, H4, H5 (Ph)), 7.45–7.48 (m, 4H, H2, H6 (Ph)). ^13^C-NMR (100.6 MHz, CDCl_3_) δ_C_ 27.9 (2C, CH_2_), 42.9 (2C, CH_2_NH), 70.7 (2C, OCH_2_), 70.8 (2C, OCH_2_), 70.9 (2C, OCH_2_), 103.3 (2C, C5, C8 (Qx)), 127.5 (2C, C4 (Ph)), 127.9 (4C, (Ph)), 129.8 (4C, (Ph)), 138.5 (2C, C4a, C8a (Qx)), 140.4 (2C, C_quat_ (Ph)), 141.8 (2C, C6, C7 (Qx)), 148.4 (2C, C2, C3 (Qx)). IR: 638 (vw), 654 (w), 668 (m), 675 (w), 700 (vs), 734 (s), 768 (m), 804 (m), 832 (m), 871 (w), 896 (w), 916 (w), 937 (w), 979 (m), 1001 (m), 1025 (s), 1066 (vs), 1079 (vs), 1089 (Vs), 1092 (vs), 1112 (vs), 1200 (vs), 1240 (vs), 1264 (s), 1353 (vs), 1437 (m), 1462 (vs), 1482 (s), 1496 (s), 1522 (vs), 1559 (w), 1599 (w), 1616 (m), 2865 (m), 2918 (m), 2942 (w), 3051 (w), 3366 (w) cm^−1^. HRMS (MALDI-TOF): *m*/*z* [M + H]^+^ calcd for C_30_H_35_N_4_O_3_^+^: 499.2704; found: 499.2665.

*2,3-Diphenyl-6,7,8,9,11,12,13,14,16,17,18,19-dodecahydro-[1,12]dioxa[5,8]diazacyclohexa decino[6,7-g]quinoxaline* (**5d**) obtained according to General Procedure from 6,7-dibromo-2,3-diphenilquinoxaline (132 mg), amine **4d** (92 mg) in the presence of Pd(dba)_2_ (14 mg), BINAP (17 mg) and *t*BuONa (87 mg) in dioxane (15 mL) in 48 h. Eluent CH_2_Cl_2_/MeOH 200:1. Yield 69 mg (48%), yellowish oil. ^1^H-NMR (400 MHz, CDCl_3_) δ_H_ 1.77 (br. s, 4H, CH_2_), 2.06 (quint, 4H, ^3^*J* = 5.1 Hz, CH_2_), 3.48 (q, 4H, ^3^*J* = 4.9 Hz, CH_2_(NH)), 3.58 (br. s, 4H, CH_2_O), 3.67 (t, 4H, ^3^*J* = 5.1 Hz, CH_2_(O)), 5.15 (br. s, 2H, NH), 7.05 (s, 2H, H5), 7.27–7.29 (m, 6H, H3, H4, H5 (Ph)), 7.46–7.48 (m, 4H, H2, H6 (Ph)). ^13^C-NMR (100.6 MHz, CDCl_3_) δ_C_ 26.3 (2C, CH_2_), 28.3 (2C, CH_2_), 44.0 (2C, CH_2_NH), 70.6 (2C, OCH_2_), 70.8 (2C, OCH_2_), 102.8 (2C, C5, C8 (Qx)), 127.5 (2C, C4 (Ph)), 128.0 (4C, (Ph)), 129.9 (4C, C2 (Ph)), 138.5 (2C, C4a, C8a (Qx)), 140.3 (2C, C_quat_ (Ph)), 141.8 (2C, C6, C7 (Qx)), 148.1 (2C, C2, C3 (Qx)). IR (neat): 669 (m), 699 (vs), 732 (m), 804 (m), 830 (s), 979 (m), 1001 (m), 1024 (s), 1065 (s), 1078 (s), 1097 (s), 1109 (s), 1199 (vs), 1240 (s), 1265 (m), 1352 (vs), 1459 (vs), 1487 (s), 1496 (s), 1522 (vs), 1560 (m), 1599 (m), 1617 (m), 2799 (m), 2854 (s), 2918 (s), 3057 (m), 3344 (m), 3388 (m) cm^−1^. HRMS (MALDI-TOF): *m*/*z* [M + H]^+^ calcd for C_30_H_35_N_4_O_2_^+^: 483.2755; found: 483.2725.

*2,3,22,23-Tetraphenyl-6,7,8,9,11,12,13,14,16,17,18,19,26,27,28,29,31,32,33,34,36,37,38,39-tetracosahydro-[1,12,17,28]tetraoxa[5,8,21,24]tetraazacyclodotriacontino[6,7-g:22,23-g’]diquinoxaline* (**6d**) obtained as a by-product in the synthesis of **5a**. Eluent CH_2_Cl_2_/MeOH 50:1. Yield 10 mg (7%), yellow-brown oil. ^1^H-NMR (400 MHz, CDCl_3_) δ_H_ 1.77 (obs.s, 8H, CH_2_), 2.07 (quint, 8H, *^3^J* = 5.4 Hz, CH_2_), 3.40–3.43 (m, 8H, CH_2_(NH)), 3.51 (obs. s, 8H, CH_2_(O)), 3.67 (t, 8H, *^3^J* = 5.4 Hz, CH_2_(O)), 4.92 (br.s., 4H, NH), 7.09 (s, 4H, H5), 7.27–7.29 (m, 12H, H3, H4, H5 (Ph)), 7.45–7.47 (m, 8H, H2, H6 (Ph)). ^13^C-NMR (100.6 MHz, CDCl_3_) δ_C_ 26.7 (4C, CH_2_), 28.5 (4C, CH_2_), 43.8 (4C, CH_2_NH), 71.0 (4C, OCH_2_), 71.3 (4C, OCH_2_), 103.7 (4C, C5, C8 (Qx)), 127.6 (4C, C4 (Ph)), 128.0 (8C, (Ph)), 129.8 (8C, C2 (Ph)), 138.5 (4C, C4a, C8a (Qx)), 140.2 (4C, C_quat_ (Ph)), 142.3 (4C, C6, C7 (Qx)), 148.5 (4C, C2, C3 (Qx)). HRMS (MALDI-TOF): *m*/*z* [M + K]^+^ calcd for C_60_H_68_N_8_O_4_K^+^: 1003.4995; found: 1003.4983.

*2,3-Diphenyl-6,7,8,9,10,11,12,13,14,15,16,17-dodecahydro-[1,4,8,11]tetraazacyclotetradecino[2,3-g]quinoxaline* (**5e**) obtained according to General Procedure from 6,7-dibromo-2,3-diphenilquinoxaline (132 mg), amine **4e** (78 mg) in the presence of Pd(dba)_2_ (14 mg), JosiPhos (15 mg) and *t*BuONa (87 mg) in dioxane (15 mL) in 48 h. Eluent CH_2_Cl_2_/MeOH 10:1. Yield 105 mg (77%), brown glassy compound. ^1^H-NMR (400 MHz, CDCl_3_/CD_3_OD) δ_H_ 2.06 (br.s, 4H, CH_2_), 2.97-3.07 (m., 8H, CH_2_(NH)), 3.45 (br.s, 4H, CH_2_(NH)), 7.01 (s, 2H, H5, H8 (Qx)), 7.26 (br.s, 6H, H3, H4, H5 (Ph)), 7.40-7.42 (m, 4H, H2, H6 (Ph)). 4 NH protons were not unambiguously assigned because of exchange with MeOD. ^13^C-NMR (100.6 MHz, CDCl_3_/CD_3_OD) δ_C_ 24.8 (2C, CH_2_), 44.3 (2C, CH_2_NH), 46.3 (2C, CH_2_NH), 49.7 (2C, CH_2_NH), 103.9 (2C, C5, C8 (Qx)), 127.9 (6C, (Ph)), 129,6 (4C, (Ph)), 138.3 (2C, C6, C7 (Qx)), 139.3 (2C, C_quat_ (Ph)), 142.1 (2C, C4a, C8a (Qx)), 148.7 (2C, C2, C3 (Qx)). IR (neat): 668 (w), 698 (s), 750 (s), 759 (s), 765 (vs), 806 (w), 834 (w), 851 (w), 917 (w), 979 (w), 1001 (w), 1025 (m), 1066 (m), 1078 (m), 1126 (m), 1157 (w), 1200 (s), 1226 (m), 1261 (m), 1267 (m), 1274 (m), 1355 (s), 1459 (s), 1494 (s), 1526 (s), 1563 (m), 1599 (m), 1619 (m), 2846 (m), 2933 (m), 2952 (m), 3054 (m), 3243 (m), 3350 (m) cm^−1^. HRMS (MALDI-TOF): *m*/*z* [M + Na]^+^ calcd for C_28_H_32_N_6_Na^+^: 475.2581; found: 475.2589.

*2,3-Diphenyl-7,8,9,10,11,12,13,14,15,16-decahydro-6H-[1,4,7,10]tetraazacyclotridecino[5,6-g]quinoxaline* (**5f**) obtained according to General Procedure from 6,7-dibromo-2,3-diphenilquinoxaline (132 mg), amine **4f** (72 mg) in the presence of Pd(dba)_2_ (14 mg), JosiPhos (15 mg) and *t*BuONa (87 mg) in dioxane (15 mL) in 48 h. Eluent CH_2_Cl_2_/MeOH 10:1. Yield 73 mg (56%), yellow-brown glassy compound. ^1^H-NMR (400 MHz, CDCl_3_/CD_3_OD) δ_H_ 1.92 (s, 2H, CH_2_), 2.86–2.95 (m, 8H, CH_2_(NH)), 3.87 (br.s, 4H, CH_2_(NH)), 5.70 (br.s., 2H, NH), 7.27–7.30 (m, 8H, H5, H8 (Qx) H3, H4, H5 (Ph)), 7.42 (m, 4H, H2, H6 (Ph)). 2 NH-protons were not unambiguously assigned because of exchange with MeOD. ^13^C-NMR (100.6 MHz, CDCl_3_/CD_3_OD) δ_C_ 24.5 (1C, CH_2_), 41.4 (2C, CH_2_NH), 45.2 (2C, CH_2_NH), 49.5 (2C, CH_2_NH), 108.5 (2C, C5, C8 (Qx)), 127.8 (4C, (Ph)), 127.9 (2C, C4 (Ph)), 129,4 (4C, (Ph)), 137.8 (2C, C6, C7 (Ph)), 139.1 (2C, C_quat_ (Ph)), 141.0 (2C, C4a, C8a (Qx)), 149.6 (2C, C2, C3 (Qx)). IR (neat): 612 (w), 624 (vw), 693 (vs), 699 (vs), 731 (m), 751 (m), 759 (m), 764 (s), 789 (vw), 810 (w), 852 (w), 904 (vw), 917 (w), 955 (vw), 979 (m), 1001 (w), 1024 (m), 1063 (s), 1077 (m), 1113 (m), 1155 (m), 1195 (s), 1217 (s), 1231 (s), 1262 (m), 1350 (s), 1377 (w), 1470 (s), 1494 (m), 1511 (m), 1531 (m), 1577 (w), 1597 (m), 1618 (m), 2846 (m), 2923 (m), 2952 (m), 3056 (m), 3334 (m) cm^−1^. HRMS (MALDI-TOF): *m*/*z* [M + H]^+^ calcd for C_27_H_31_N_6_^+^: 439.2605; found: 439.2629.

*2,3-Diphenyl-7,8,9,10,11,12,13,14,15,16,17,18-dodecahydro-6H-[1,4,8,12]tetraazacyclopenta decino[2,3-g]quinoxaline* (**5g**) obtained according to General Procedure from 6,7-dibromo-2,3-diphenilquinoxaline (132 mg), amine **4g** (85 mg) in the presence of Pd(dba)_2_ (14 mg), JosiPhos (15 mg) and *t*BuONa (87 mg) in dioxane (15 mL) in 48 h. Eluent CH_2_Cl_2_/MeOH 10:1. Yield 71 mg (50%), yellow-brown glassy compound. ^1^H-NMR (400 MHz, CDCl_3_/CD_3_OD) δ_H_ 2.00–2.06 (m, 6H, CH_2_), 3.01 (t, 4H, ^3^*J* = 6.4 Hz, CH_2_(NH)), 3.04–3.08 (m, 4H, CH_2_(NH)), 3.37–3.39 (m, 4H, CH_2_(NH)), 6.85 (s, 2H, H5, H8 (Qx)), 7.04–7.07 (m, 6H, (Ph)), 7.12–7.15 (m, 4H, (Ph)). 4NH-protons were not unambiguously assigned because of exchange with MeOD and overlapping. ^13^C-NMR (100.6 MHz, CDCl_3_/CD_3_OD) δ_C_ 23.3 (1C, CH_2_), 24.0 (2C, CH_2_), 42.3 (2C, CH_2_NH), 46.7 (2C, CH_2_NH), 49.7 (2C, CH_2_NH), 104.2 (2C, C5, C8 (Qx)), 127.8 (2C, C4 (Ph)), 127.9 (4C, (Ph)), 129,6 (4C, (Ph)), 138.2 (2C, C6, C7 (Qx)), 139.4 (2C, C_quat_ (Ph)), 140.9 (2C, C4a, C8a (Qx)), 148.9 (2C, C2, C3 (Qx)). IR (neat): 649 (w), 659 (w), 663 (w), 668 (s), 674 (w), 679 (w), 698 (m), 721 (vw), 734 (w), 766 (w), 792 (vw), 816 (w), 834 (w), 845 (vw), 924 (vw), 985 (w), 1001 (w), 1025 (w), 1082 (m), 1097 (m), 1118 (m), 1179 (s), 1217 (s), 1255 (m), 1268 (m), 1288 (m), 1332 (m), 1354 (w), 1363 (w), 1395 (m), 1405 (m), 1419 (m), 1430 (m), 1437 (m), 1448 (m), 1457 (m), 1465 (m), 1472 (m), 1491 (m), 1496 (m), 1507 (m),1521 (w), 1540 (m), 1559 (w), 1576 (m), 1606 (m), 1617 (m), 1653 (m), 1684 (w), 1700 (m), 1718 (m), 1730 (s), 1734 (s), 1740 (s), 1743 (s), 2926 (m), 2951 (m), 3054 (m), 3296 (m) cm^−1^. HRMS (MALDI-TOF): *m*/*z* [M + H]^+^ calcd for C_29_H_35_N_6_^+^: 467.2918; found: 467.2947.

*2,3-Diphenyl-7,8,9,10,11,12,13,14,15,16,17,18-dodecahydro-6H-[1,4,7,10,13]pentaazacyclo pentadecino[2,3-g]quinoxaline* (**5h**) obtained according to General Procedure from 6,7-dibromo-2,3-diphenilquinoxaline (132 mg), amine **4h** (85 mg) in the presence of Pd(dba)_2_ (14 mg), JosiPhos (15 mg) and *t*BuONa (87 mg) in dioxane (15 mL) in 48 h. Eluent CH_2_Cl_2_/MeOH 10:1. Yield 30 mg (21%), yellow-brown glassy compound. ^1^H-NMR (400 MHz, CDCl_3_/CD_3_OD) δ_H_ 2.78 (br.s, 4H, CH_2_(NH)), 3.05 (br.s, 4H, CH_2_(NH)), 3.23 (br.s, 4H, CH_2_(NH)), 3.63 (s, 4H, CH_2_(NH)), 5.57 (br. s, NH), 6.84 (s, 2H, H5, H8 (Qx)), 7.11–7.19 (m, 10H, H2, H3, H4, H5, H6 (Ph)). 3 NH-protons were not unambiguously assigned because of exchange with MeOD and overlapping. ^13^C-NMR (100.6 MHz, CDCl_3_/CD_3_OD) δ_C_ 38.9 (2C, CH_2_NH), 44.6 (2C, CH_2_NH), 45.1 (2C, CH_2_NH), 46.7 (2C, CH_2_NH), 103.7 (2C, C5, C8), 127.8 (6C, (Ph)), 129,5 (4C, (Ph)), 138.2 (2C, C6, C7 (Qx)), 139.3 (2C, C_quat_ (Ph)), 139.8 (2C, C4a, C8a (Qx)), 149.1 (2C, C2, C3 (Qx)). IR (neat): 611 (m), 618 (m), 651 (m), 669 (m), 698 (vs), 732 (s), 777 (m), 807 (m), 835 (m), 919 (w), 978 (m), 1024 (m), 1065 (s), 1131 (m), 1157 (m), 1199 (s), 1242 (s), 1264 (m), 1288 (m), 1355 (s), 1456 (s), 1473 (s), 1481 (s), 1496 (s), 1507 (m), 1521 (m), 1535 (m), 1539 (s), 1559 (m), 1576 (m), 1599 (m), 1617 (m), 2750 (m), 2853 (m), 2928 (m), 2958 (m), 3054 (m), 3313 (m), 3329 (m) cm^−1^. HRMS (MALDI-TOF): *m*/*z* [M + Na]^+^ calcd for C_28_H_33_N_7_Na^+^: 490.2690; found: 490.2689.

NMR spectra of the compounds are given in the Supplementary Materials.

### 3.4. Protonation Studies

Protonation studies of compounds **5c** and **5e** were performed at room temperature. The solutions were prepared with double-deionized high-purity water (18.2 MΩ cm) obtained from a «Millipore Simplicity» apparatus (Merck Co., Rahway, NJ, USA). Solution concentrations and other experiment conditions are given in the title of the corresponding figures and tables. Protonation studies were conducted in a glass cell equipped with a magnetic stirrer and pH-electrode, adding HClO_4_ (4 M or 0.01 M) or NaOH (5 M or 0.01 M) to the solutions of ligands (*I* = 0.1 M, NaClO_4_). The pK_a_, spectra of the species and speciation diagrams were calculated using nonlinear least-squares analysis by means of the HypSpec program [79] after factor analysis of the combined data sets [80]. The goodness of fit was assessed through the scaled standard deviation of the residuals (s), which has an expected value of unity in the absence of systematic errors assuming a correct weighting scheme. UV and fluorescence spectra, calculated spectra of the species, speciation diagrams and titration curves are given in the Supplementary Materials.

### 3.5. Metal Ions Complexation Studies

Complexation studies of compounds **3** and **5a**–**d** were performed at room temperature in acetonitrile. Metal-binding experiments were conducted by manual addition of the aliquots of metal salt solutions in acetonitrile by a Hamilton syringe to a solution of ligand placed in a quartz cuvette. All metal salts used were perchlorates of the general M(ClO_4_)*n*·*x*H_2_O formula. *Caution! Although no problems were experienced, perchlorate salts are potentially explosive when combined with organic ligands and should be manipulated with care and used only in very small quantities.* 18 metal ions *(*Li^+^, Na^+^, K^+^, Mg^2+^, Ca^2+^, Sr^2+^, Ba^2+^, Al^3+^, Cr^3+^, Mn^2+^, Fe^2+^, Co^2+^, Ni^2+^, Cu^2+^, Zn^2+^, Ag^+^, Cd^2+^, Pb^2+^ and Hg^2+^) were tested. Solutions of metal perchlorates were prepared with concentrations of about 1000-fold that of the ligands in order to decrease the influence of the medium changes on the spectra of the studied solutions. Stability constants, spectra of the species and speciation diagrams were calculated using nonlinear least-squares analysis by means of the HypSpec program [79] after factor analysis of the combined data sets [80]. The goodness of fit was assessed through the scaled standard deviation of the residuals (s), which has an expected value of unity in the absence of systematic errors assuming a correct weighting scheme. The results were checked by plotting calculated molar extinction graphs. UV and fluorescence spectra, calculated spectra of the species, speciation diagrams and titration curves are given in the Supplementary materials.

## 4. Conclusions

To sum up, we elaborated an approach to polyoxadiaza- and polyazamacrocycles **5a**–**h** comprising 6,7-diamino-2,3-diphenylquinoxaline moiety in yields up to 77% using Pd-catalyzed amination reaction. Compounds with oxadiamino chains can be obtained using a classical Pd(dba)_2_/BINAP catalytic system, whereas macrocycles with tetraamino chains demand the application of an advanced Pd(dba)_2_/JosiPhos system. Compounds thus obtained possess strong luminescence in various solvents, which can be detected by a naked eye. Macrocycles **5a**–**d** were tested for metal cations detection in acetonitrile but did not demonstrate selectivity. Thus, one needs to further modify the receptor unit to achieve this goal. Protonation studies of the macrocycles **5c** and **5e** were carried out using spectrophotometric and spectrofluorimetric titrations, and their protonation constants were calculated. Significant changes in their absorption and emission spectra at different pH, including a possibility of a naked-eye inspection, make them attractive dual channel indicators working in acidic media. The fabrication of thin films employing the compounds obtained in this work and their derivatives will be the next important step in the application of the sensing abilities of the quinoxaline-based detectors.

## Figures and Tables

**Table 1:** Photophysical data for compounds **3** and **5a–h** in MeCN.

Compound	λ_abs_, nm (log ε)	λ_em_, nm	Φ, % ^a^	Brightness ^b^, B, Μ^−1^ cm^−1 c^
**3**	261 (4.51) 279 (4.50) 404 (4.36)	465	59	13,494
**5a**	260 (4.37) 282 (4.34) 408 (4.20)	485	52	8241
**5b**	260 (4.48) 279 (4.46) 404 (4.35)	465	48	10,746
**5c**	262 (4.38) 282 (4.35) 409 (4.24)	465	44	9674
**5d**	263 (4.51) 280 (4.47) 410 (4.41)	466	56	14,394
**5e**	257 (4.39) 278 (4.36) 400 (4.19)	480	44	6795
**5f**	281 (4.29) 404 (4.11)	486	68	8760
**5g**	260 (4.30) 279 (4.29) 404 (4.19)	464	65	10,067
**5h**	262 (4.39) 280 (4.37) 406 (4.26)	465	58	10,554

^a^ Quantum yields were determined using quinine sulfate in 0.05M H_2_SO_4_ (Φ = 53%) as a standard.^b^ *B = Φ(λ) × ε (λ_ex_)* [68], the lowest energy absorption band (ICT) was used for calculation.

**Table 2:** Calculated stability constants of the complexes of **3** and **5a**–**d** Cu(II) ions in acetonitrile.

Ligand	Complex	log(β) ^a^
**3**	[Cu(**3**)]^2+^ [Cu_2_(**3**)]^4+^ [Cu_3_(**3**)]^6+^	7.57(7) 13.3(3) 18.7(2)
**5a**	[Cu(**5a**)]^2+^ [Cu_4_(**5a**)_3_]^8+^	6.53(1) 33.9(3)
**5b**	[Cu(**5b**)]^2+^ [Cu_4_(**5b**)_3_]^8+^	6.73(1) 34.7(1)
**5c**	[Cu(**5c**)]^2+^ [Cu_4_(**5c**)_2_]^8+^	6.47(1) 28.3(3)
**5d**	[Cu(**5d**)]^2+^ [Cu_4_(**5d**)_2_]^8+^	7.01(1) 28.7(1)

^a^ The stability constants were calculated from spectrophotometric titration data using the HypSpec program.

**Table 3:** Apparent stepwise protonation of polyamines **5a** and **5e** and relevant compounds **7**–**9** reported in the literature.

Compound	Method	pK(N_alk_)	pK(N_alk_)	pK(N_Qx_)	pK(N_Qx_)
**5c** ^a^	UV-vis ^b^	–	–	4.10(5)	0.6(1)
	FL ^c^	–	–	3.96(5)	1.20(5)
**5e** ^a^	UV-vis ^b^	{9.5} ^d^	3.69(5)	2.36(5)	– ^e^
	FL ^c^	{9.5} ^d^	4.01(5)	2.46(5)	1.0(1)
**7** ^f^	UV-vis ^b^	10.25	10.25	2.61	–
	FL ^c^	9.32	9.32	3.07	–
**8** ^g^	PT ^h^	9.40	6.52	–	–
**9** ^g^	PT ^h^	9.05	3.82	–	–

^a^ *I* = 0.1 M NaClO_4_, *T* = 298(2) K. ^b^ UV-vis spectrophotometric measurements. ^c^ Fluorescence measurements. ^d^ Only estimation of the protonation constant can be done because of precipitation. ^e^ Not observed. ^f^ Ref. [36]. ^g^ Ref. [70]. ^h^ Potentiometric measurements.

## Data Availability

Not applicable.

## Supplementary Materials

The following supporting information can be downloaded at: https://www.mdpi.com/article/10.3390/molecules28020512/s1, Table S1: Photophysical data for compounds **3**, **5c** and **5e** in various solvents. Figures S1–S6: UV-vis and fluorescence spectra of **3**, **5c** and **5e** in toluene, dioxane, CH_2_Cl_2_, MeCN and MeOH. Figures S7–S12: Dependence of UV-vis and fluorescence spectra of **3**, **5c** and **5e** in aqueous solutions on concentration. Figures S13, S17, S21, S25 and S29: Luminescence and absorption spectra of **3** and **5a**–**d** in acetonitrile before and after addition of 5 equiv. of metal perchlorate salts. Figures S14, S18, S22, S26 and S30: Evolution of the emission spectra of **3** and **5a–d** in acetonitrile upon addition of various quantities of Cu(ClO_4_)_2_. Figures S15, S19, S23, S27 and S31: Evolution of the UV-vis absorption spectra of **3** and **5a–d** in acetonitrile upon addition of various quantities of Cu(ClO_4_)_2_. Figures S16, S20, S24, S28 and S32: UV-vis spectra of copper complexes of **3** and **5a**–**d** and species distribution diagrams in acetonitrile calculated using the HypSpec program. Figures S33, S34, S39, S40: Spectrophotometric titration of **5c** and **5e** as a function of pH. Figures S35 and S41: UV-vis spectra of protonated species of **5c** and **5e** and their distribution diagrams in aquatic media calculated using the HypSpec program. Figures S36, S37, S42, S43: fluorimetric titration of **5c** and **5e** as a function of pH. Figures S38 and S44: fluorescence spectra of protonated species of **5c** and **5e** and their distribution diagrams in aquatic media calculated using the HypSpec program. Figures S45 and S46: UV-vis spectra and luminescence spectra of **5e** aqueous solutions at pH 7–10. Figures S47–S69: 1H and ^13^C NMR spectra of the compounds **1**, **3**, **5a**–**h**, **6a** and **6d**.

## Sample Availability

Samples of the compounds are available from the authors (A.S.A.).
