# Supplementary material for: Polyoxa- and Polyazamacrocycles Incorporating 6,7-Diaminoquinoxaline Moiety: Synthesis and Application as Tunable Optical pH-Indicators in Aqueous Solution
Source: Molecules. 2023 Jan 4;28(2):512. doi: 10.3390/molecules28020512 (PMC9866286; doi:10.3390/molecules28020512)
Supplement: Supplementary file 1 [file molecules-28-00512-s001.zip › molecules-2132820-supplementary.pdf]

Supporting information

for the article

Polyoxa- and Polyazamacrocycles Incorporating 6,7-diaminoquinoxaline Moiety: Synthesis and Application as Tunable Optical pH-Indicators in Aqueous Solution

by
Igor A. Kurashov, Alisa D. Kharlamova, Anton S. Abel,
Alexei D. Averin, and Irina P. Beletskaya

Table of content

1. Spectroscopic studies of **3**, **5c** and **5e** in organic solutions S3
2. Spectroscopic studies of **3, 5c** and **5e** in aqueous solutions S6
3. Studies of **3** and **5a–d** for metal cations binding in acetonitrile S9
4. Protonation studies of **5c** and **5e** S19
5. NMR spectra of new compounds S24
6. References S36

1.Spectroscopic Studies of 3, 5c and 5e in Organic Solvents.

Table S1. Photophysical data for compounds **3**, **5c** and **5e** in various solvents

Compound	Solvent	λ_{abs}, nm (log ε)[a]	λ_{em}, nm	Φ, %[b]	Brightness, B, M^{-1} cm^{-1} [c]
**3**	toluene	400 (4.35)	453	34	7597
	dioxane	258 (4.49)<br>281 (4.47)<br>404 (4.36)	450	53	12261
	CH_2Cl_2	402 (4.25)	465	52	9296
	MeCN	261 (4.51)<br>279 (4.50)<br>404 (4.36)	465	59	13494
	MeOH	261 (4.62)<br>279 (4.53)<br>416 (4.36)	482	48	10978
	H_2O (pH = 6) [c]	256 (4.22)<br>277 (4.18)<br>412 (4.06)	507	27	3132
**5c**	toluene	408 (4.29)	450	50	12492
	dioxane	261 (4.24)<br>283 (4.22)<br>408 (4.14)	451	70	12125
	CH_2Cl_2	410 (4.14)	462	59	10220
	MeCN	262 (4.38)<br>282 (4.35)<br>409 (4.24)	465	44	9674
	MeOH	261 (4.45)<br>279 (4.39)<br>420 (4.16)	486	64	11726
	H_2O (pH = 6) [c]	256 (4.19)<br>277 (4.14)<br>418 (4.06)	510	34	3944
**5e**	toluene	416 (4.30)	452	42	8355
	dioxane	262 (4.31)<br>283 (4.30)<br>411 (4.17)	456	33	4837
	CH_2Cl_2	405 (3.94)	476	42	3628
	MeCN	257 (4.39)<br>278 (4.36)<br>400 (4.19)	480	44	6795
	MeOH	256 (4.39)<br>279 (4.34)	498	60	9423

		412 (4.20)			
	H_2O (pH = 6) [c]	256 (4.38)<br>277 (4.33)<br>407 (4.15)	526	36	5148

[a]Molar extinction coefficient (ε) is expressed in M^{-1} cm^{-1}. [b] Quantum yields were determined using quinine sulfate in 0.05M H_2SO_4 (Φ = 53%) as a standard. [c] $B = \Phi(\lambda) \times \varepsilon(\lambda_{ex})$.[1], the lowest absorption band (ICT) was used for the calculation. [c] 1 vol% MeOH in H_2O.

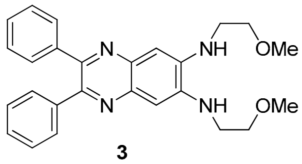

3

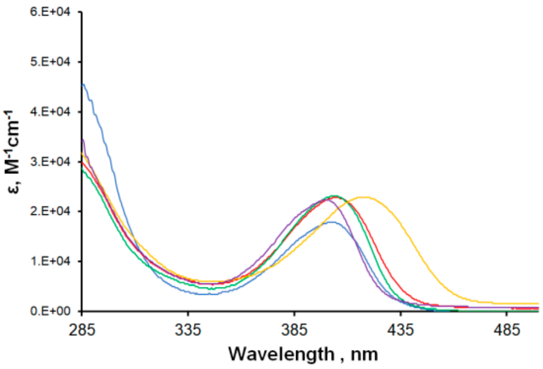

Figure S1. UV-vis spectra of **3** in toluene (violet), dioxane (green), CH_2Cl_2 (blue), MeCN (red) and MeOH (yellow).

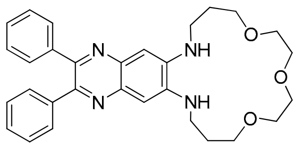

5c

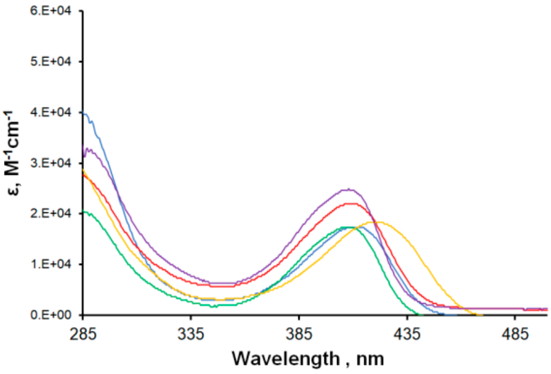

Figure S2. UV-vis spectra of **5c** in toluene (violet), dioxane (green), CH_2Cl_2 (blue), MeCN (red) and MeOH (yellow).

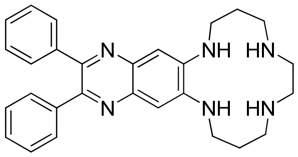

5e

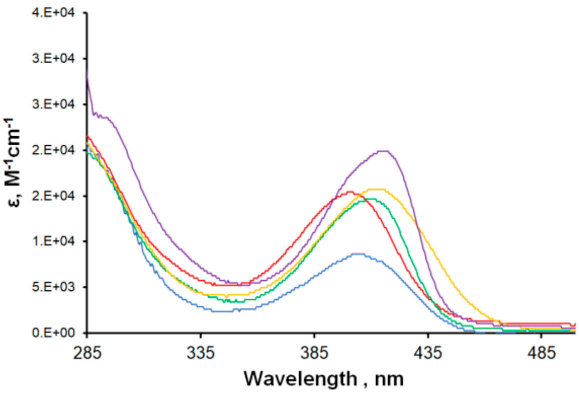

Figure S3. UV-vis spectra of **5e** in toluene (violet), dioxane (green), CH_2Cl_2 (blue), MeCN (red) and MeOH (yellow).

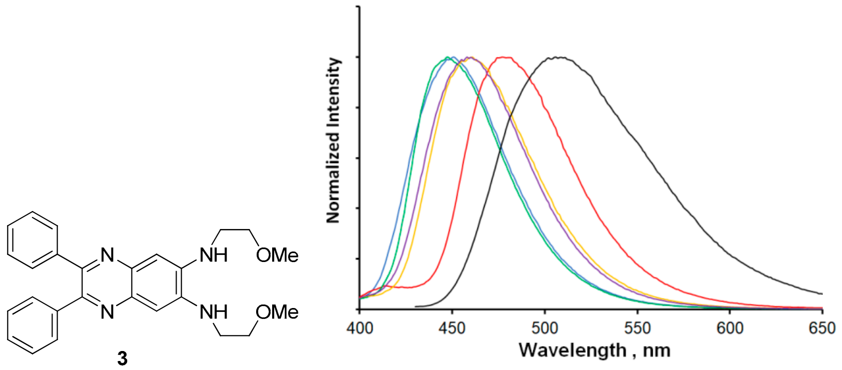

Figure S4. Normalized fluorescence spectra of **3** (λ_{ex} = 420 nm) in toluene (blue), dioxane (green), CH_2Cl_2 (violet), MeCN (yellow), MeOH (red), water (1 vol% MeOH, pH = 6, black).

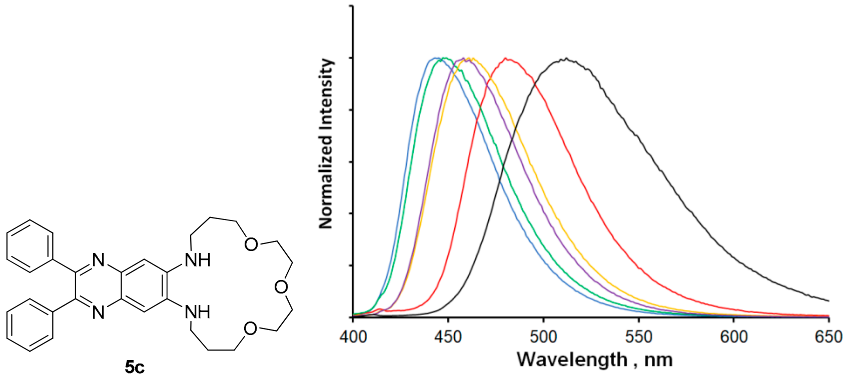

Figure S5. Normalized fluorescence spectra of **5c** (λ_{ex} = 420 nm) in toluene (blue), dioxane (green), CH_2Cl_2 (violet), MeCN (yellow), MeOH (red), water (1 vol% MeOH, pH = 6, black).

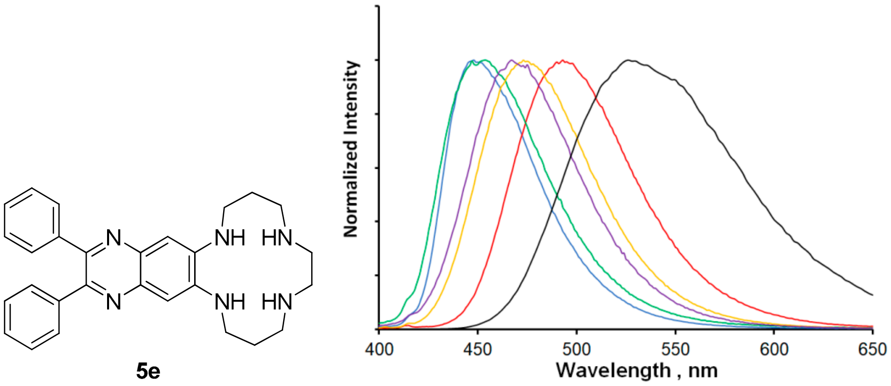

Figure S6. Normalized fluorescence spectra of **5c** (λ_{ex} = 420 nm) in toluene (blue), dioxane (green), CH_2Cl_2 (violet), MeCN (yellow), MeOH (red), water (pH = 6, black). The spectrum in water corresponds [**5e**H]$^+$.

2. Spectroscopic studies of 3, 5c and 5e in aqueous solutions

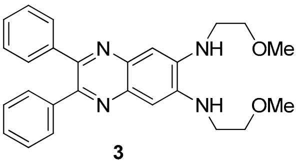

3

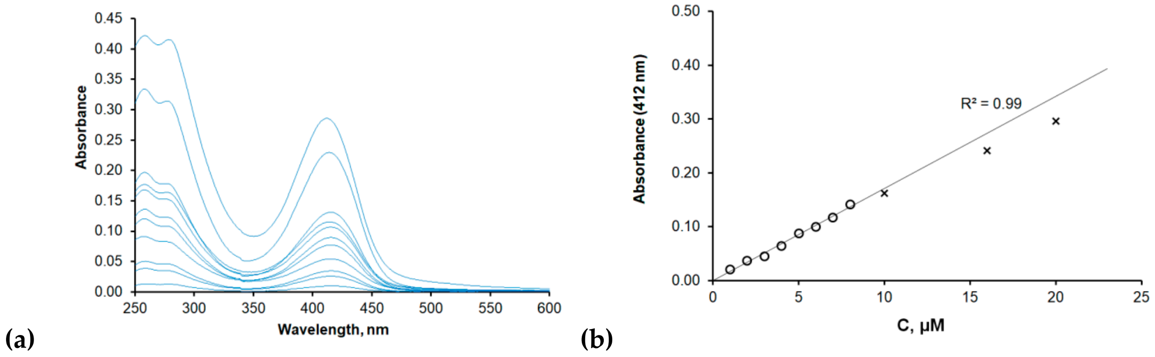

Figure S7. (**a**) UV-vis spectra of **3** in aqueous solutions (1% MeOH, pH = 6). (**b**) Dependence of the absorbance intensity on **3** concentration in aqueous solutions (1 vol% MeOH, pH = 6).

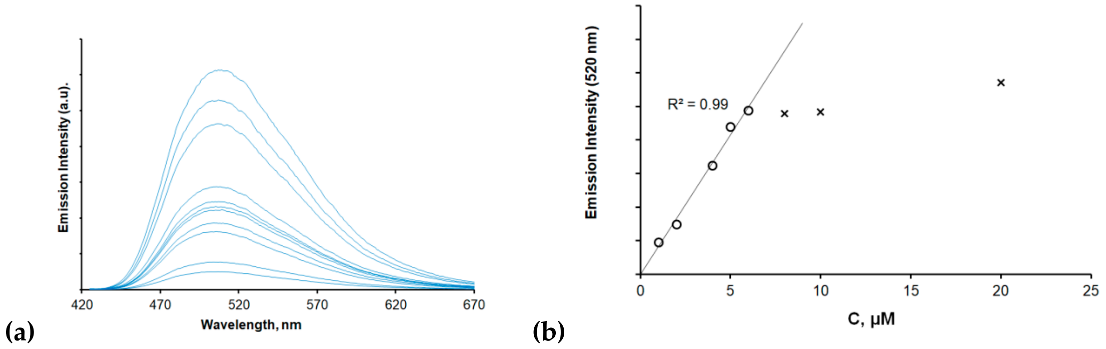

Figure S8. (**a**) Fluorescence spectra of **3** in aqueous solutions (1 vol % MeOH, pH = 6, λ_{ex} = 415 nm). (**b**) Dependence of the emission intensity on **3** concentration in aqueous solutions (1 vol % MeOH, pH = 6, λ_{ex} = 415 nm).

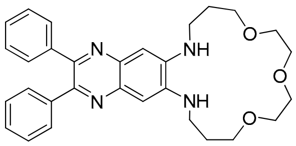

5c

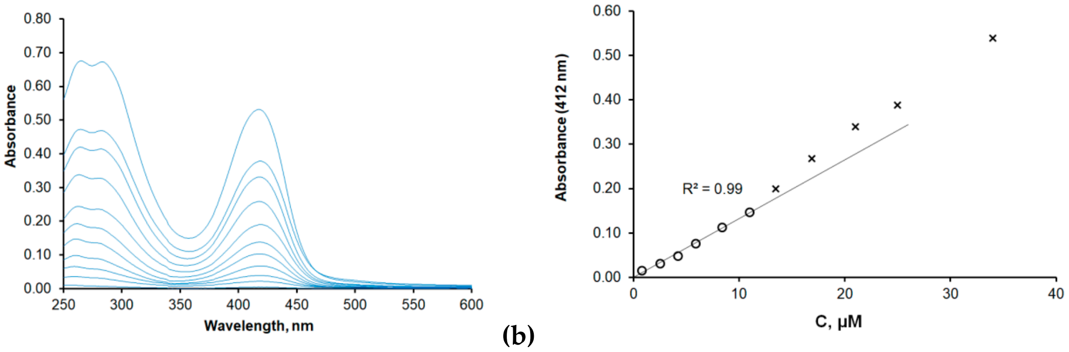

(a) **(b)**

Figure S9. (**a**) UV-vis spectra of **5c** in aqueous solutions (1 vol % MeOH, pH = 6). (**b**) Dependence of the absorbance intensity on **5c** concentration in aqueous solutions (1 vol % MeOH, pH = 6).

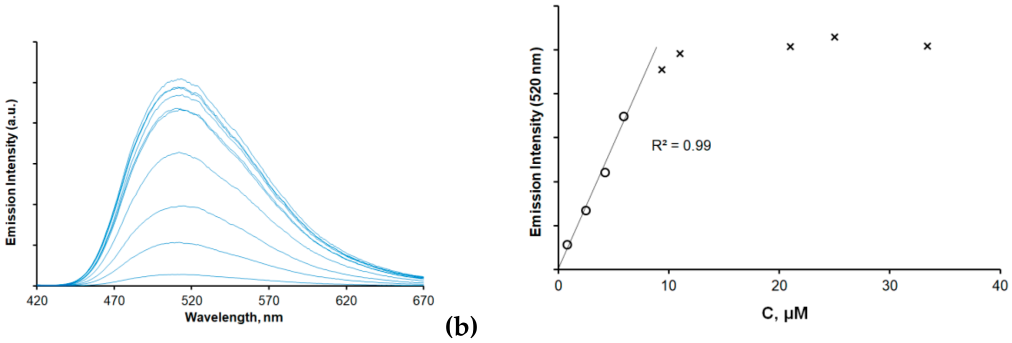

(a) **(b)**

Figure S10. (**a**) Fluorescence spectra of **5c** in aqueous solutions (1 vol% MeOH, pH = 6, λ_{ex} = 415 nm). (**b**) Dependence of the emission intensity on **5c** concentration in aqueous solutions (1 vol% MeOH, pH = 6, λ_{ex} = 415 nm).

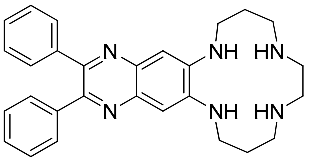

5e

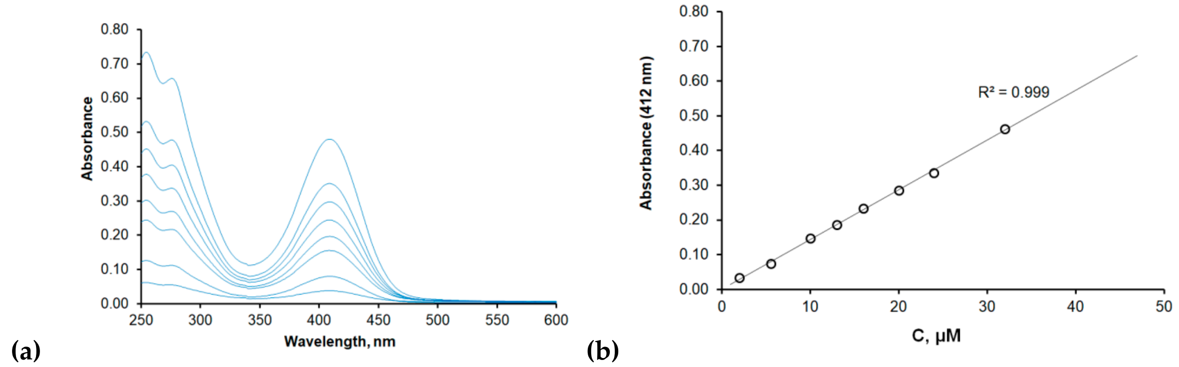

Figure S11. (**a**) UV-vis spectra of **5e** in aqueous solutions (pH = 6). (**b**) Dependence of the absorbance intensity on **5e** concentration in aqueous solutions (pH = 6).

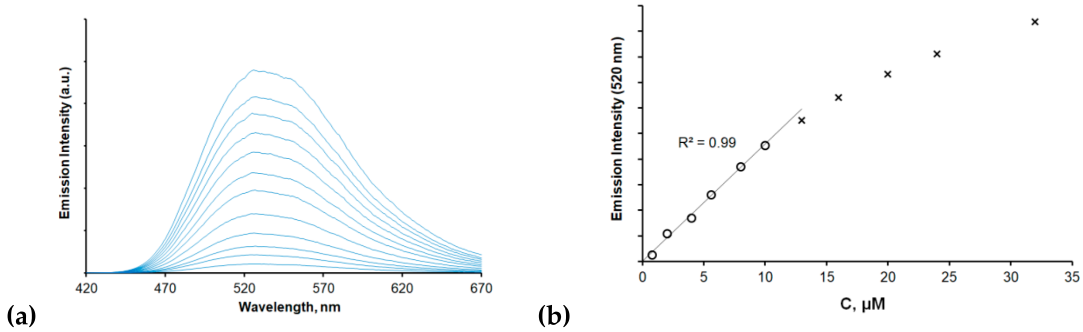

Figure S12. (**a**) Fluorescence spectra of **5e** in aqueous solutions (pH = 6, λ_{ex} = 415 nm). (**b**) Dependence of the emission intensity on **5e** concentration in aqueous solutions (pH = 6, λ_{ex} = 415 nm).

3. Studies of 3 and 5a-d for Metal Cations Binding in Acetonitrile.

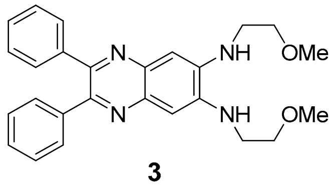

3

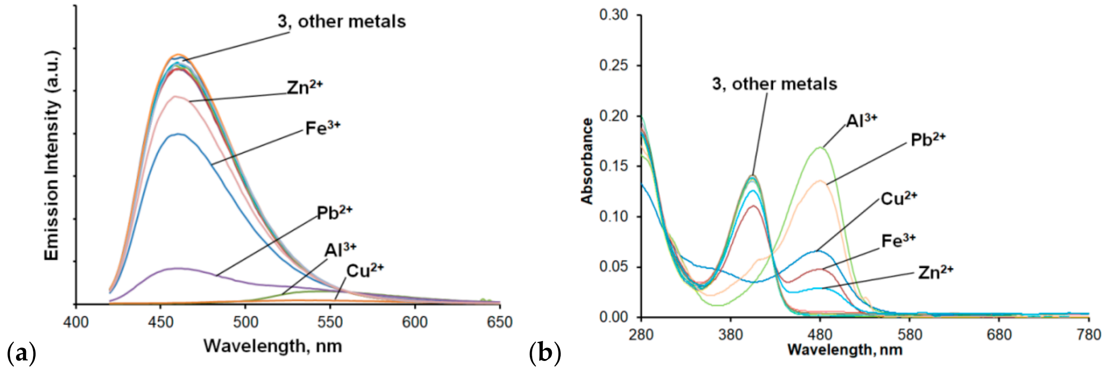

Figure S13. Luminescence (**a**) and absorption (**b**) spectra of **3** in acetonitrile ([3] = 6.0 μM, λ_{ex} = 405 nm) before and after addition of 5 equiv. of metal perchlorate salts.

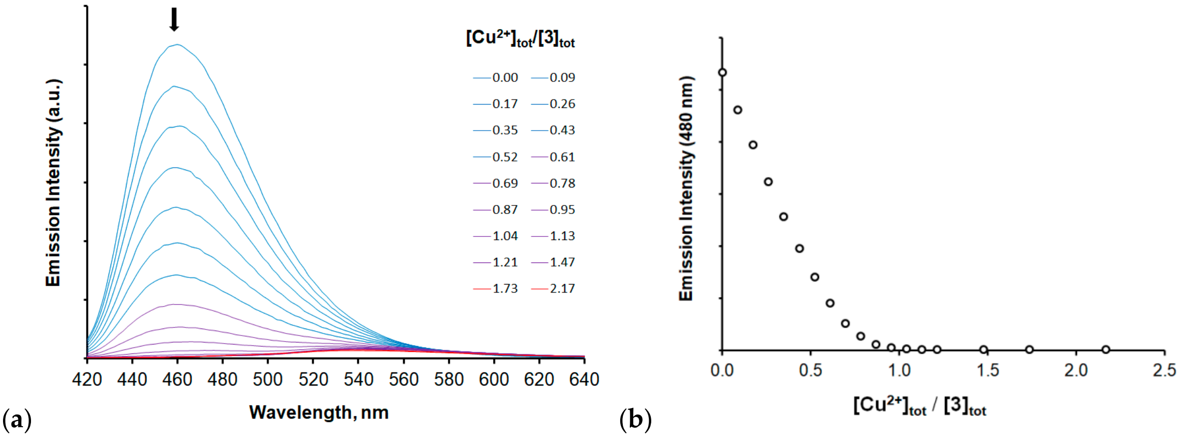

Figure S14. (**a**) Evolution of the emission spectrum of **3** in acetonitrile ([**3**]$_{tot}$ = 6.0 μM, λ_{ex} = 405 nm) upon addition of $Cu(ClO4)_2$ (0–2.1 equiv.). (**b**) Changes of the emission intensity as a function of the $[Cu^{2+}]_{tot}$/[**3**]$_{tot}$ ratio at λ_{em} = 480 nm.

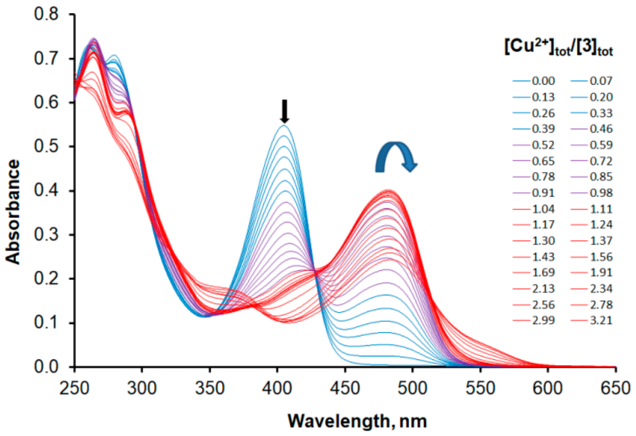

(**a**)

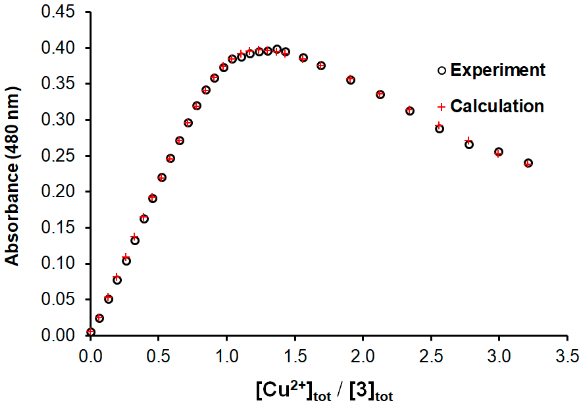

(**b**)

Figure S15. Evolution of the UV–vis absorption spectrum of **3** in acetonitrile ([**3**] = 23.9 μM) upon addition of Cu(ClO4)2 (0–3.2 equiv.). Changes of the absorbance as a function of the $[Cu^{2+}]_{tot}/[\mathbf{3}]_{tot}$ ratio at $\lambda = 480$ nm.

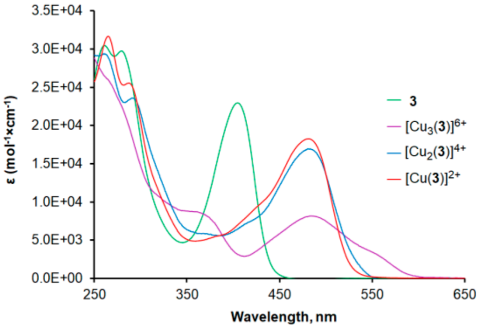

(**a**)

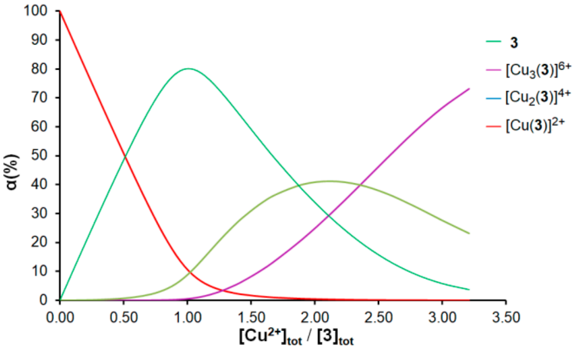

(**b**)

Figure S16. UV–vis spectra of **3**, $[Cu(\mathbf{3})]^{2+}$, $[Cu_2(\mathbf{3})]^{4+}$ and $[Cu_3(\mathbf{3})]^{6+}$ in acetonitrile calculated using HypSpec program.[2] (**b**) Species distribution diagram for the **3**/Cu^{2+} system in acetonitrile calculated using HypSpec program.[2] Data were fit with HypSpec using the following model:

$Cu^{2+} + L \leftrightarrows [CuL]^{2+}$ (log β = 7.57(7))

$2Cu^{2+} + L \leftrightarrows [Cu_2L]^{4+}$ (log β = 13.3(3))

$3Cu^{2+} + L \leftrightarrows [Cu_3L]^{6+}$ (log β = 18.7(2))

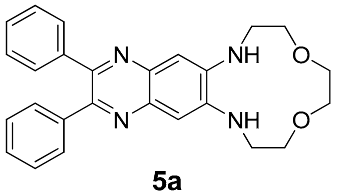

5a

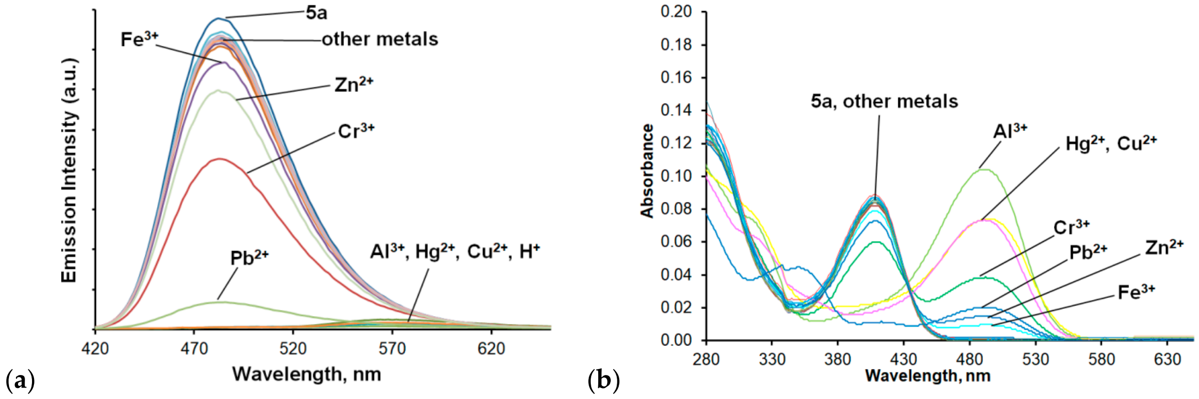

Figure S17. Luminescence (**a**) and absorption (**b**) spectra of **5a** in acetonitrile ([**5a**] = 5.2 μM, λ_{ex} = 408 nm) before and after addition of 5 equiv. of metal perchlorate salts.

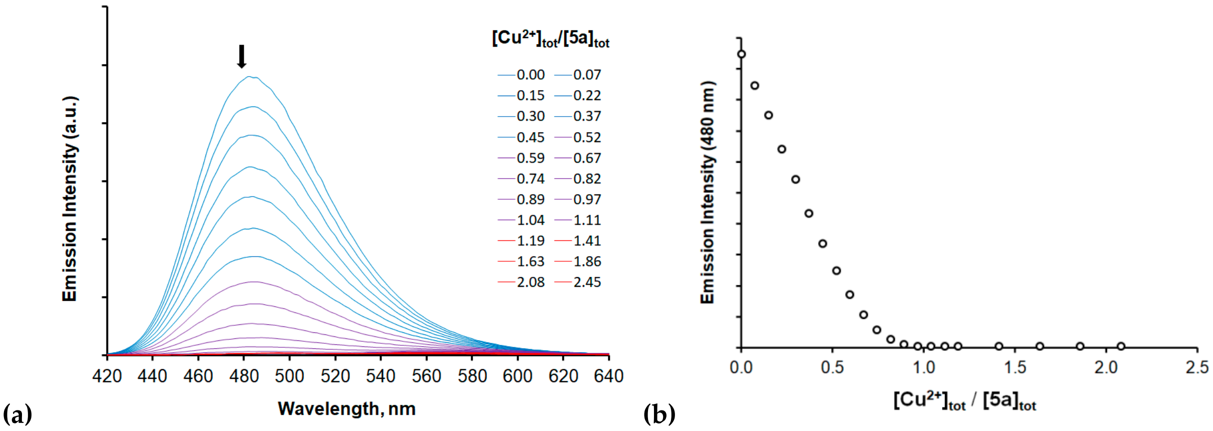

Figure S18. (a) Evolution of the emission spectrum of **5a** in acetonitrile ([**5a**]$_{tot}$ = 5.2 μM, λ_{ex} = 408 nm) upon addition of $Cu(ClO4)_2$ (0–2.4 equiv.). **(b)** Changes of the emission intensity as a function of the $[Cu^{2+}]_{tot}$/[**5a**]$_{tot}$ ratio at λ_{em} = 480 nm.

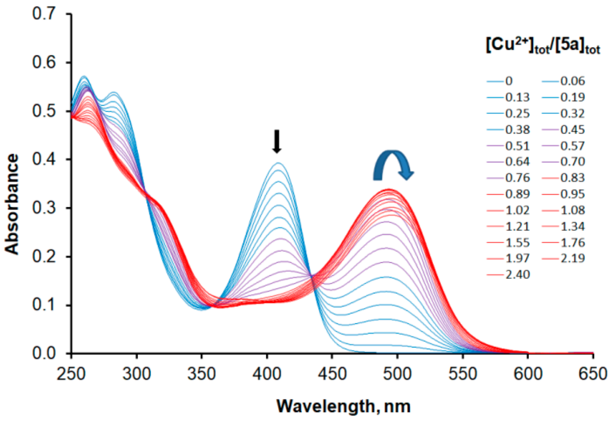

(**a**)

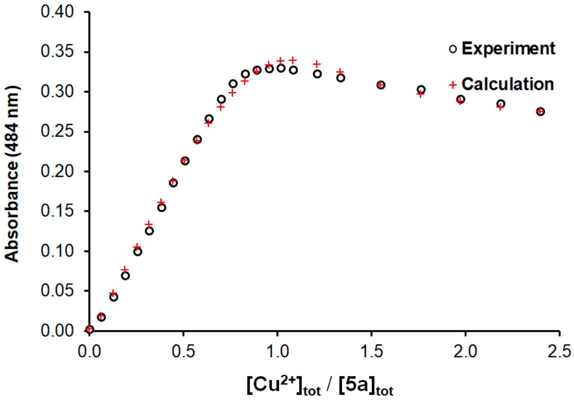

(**b**)

Figure S19. (**a**) Evolution of the UV–vis absorption spectrum of **5a** in acetonitrile ([**5a**] = 24.4 μM) upon addition of Cu(ClO4)2 (0–2.4 equiv.). (**b**) Changes of the absorbance as a function of the $[Cu^{2+}]_{tot}/[\mathbf{5a}]_{tot}$ ratio at λ =484 nm.

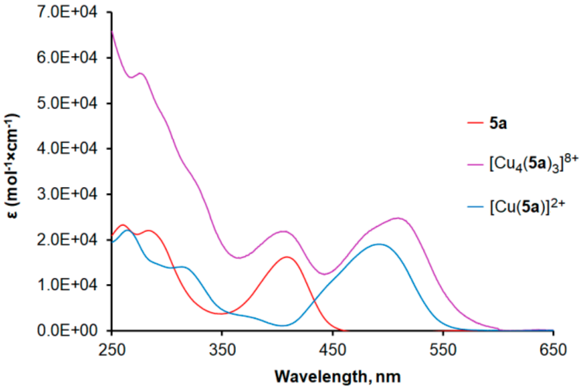

(a)

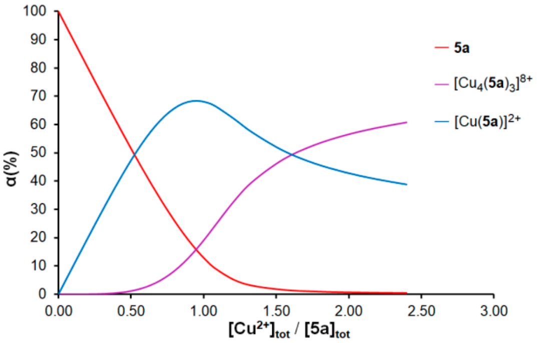

(b)

Figure S20. (**a**) UV–vis spectra of **5a**, $[Cu(\mathbf{5a})]^{2+}$ and $[Cu_4(\mathbf{5a})_3]^{8+}$ in acetonitrile calculated using HypSpec program.[2] (**b**) Species distribution diagram for the **5a**/Cu^{2+} system in acetonitrile calculated using HypSpec program.[2] Data were fit with HypSpec using the following model:

$Cu^{2+} + L \leftrightarrows [CuL]^{2+}$ (log β= 6.53(1))

$4Cu^{2+} + 3L \leftrightarrows [Cu_4L_3]^{8+}$ (log β= 33.9(3))

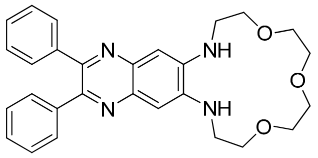

5b

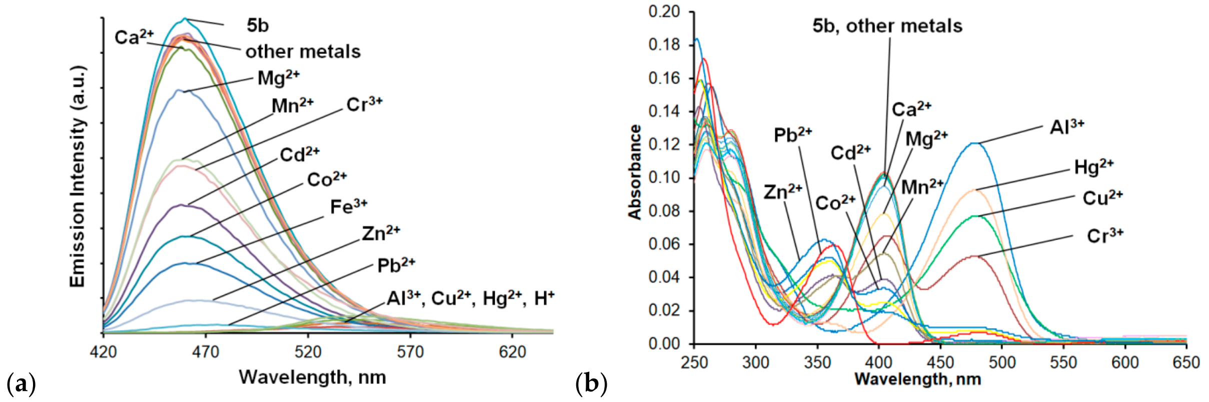

Figure S21. Luminescence (**a**) and absorption (**b**) spectra of **5b** in acetonitrile ([**5b**] = 4.8 μM, λ_{ex} = 404 nm) before and after addition of 5 equiv. of metal perchlorate salts.

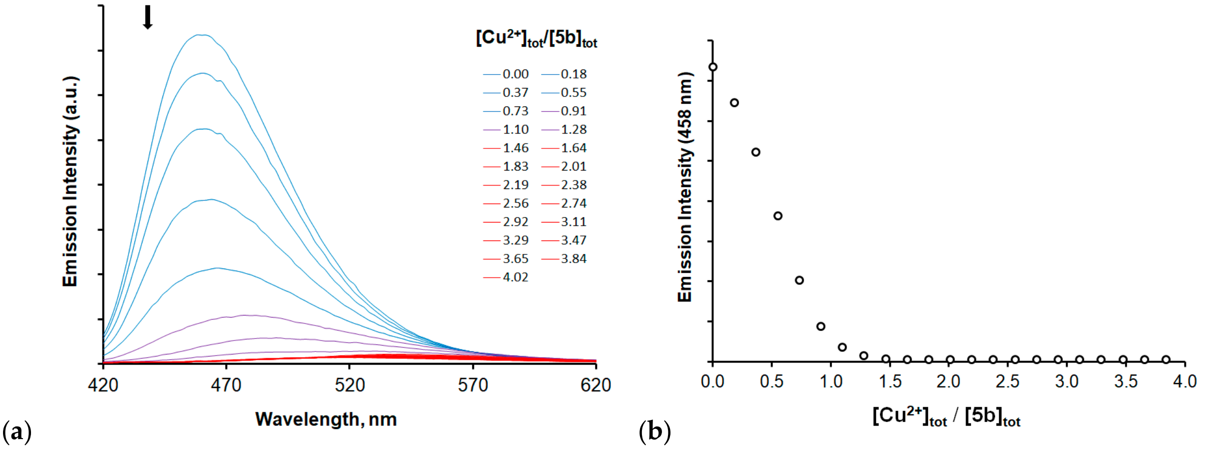

Figure S22. (**a**) Evolution of the emission spectrum of **5b** in acetonitrile ([**5b**]$_{tot}$ = 4.8 μM, λ_{ex} = 404 nm) upon addition of Cu(ClO4)2 (0– 4 equiv.). (**b**) Changes of the emission intensity as a function of the [Cu^{2+}]$_{tot}$/[**5b**]$_{tot}$ ratio at λ_{em} = 458 nm.

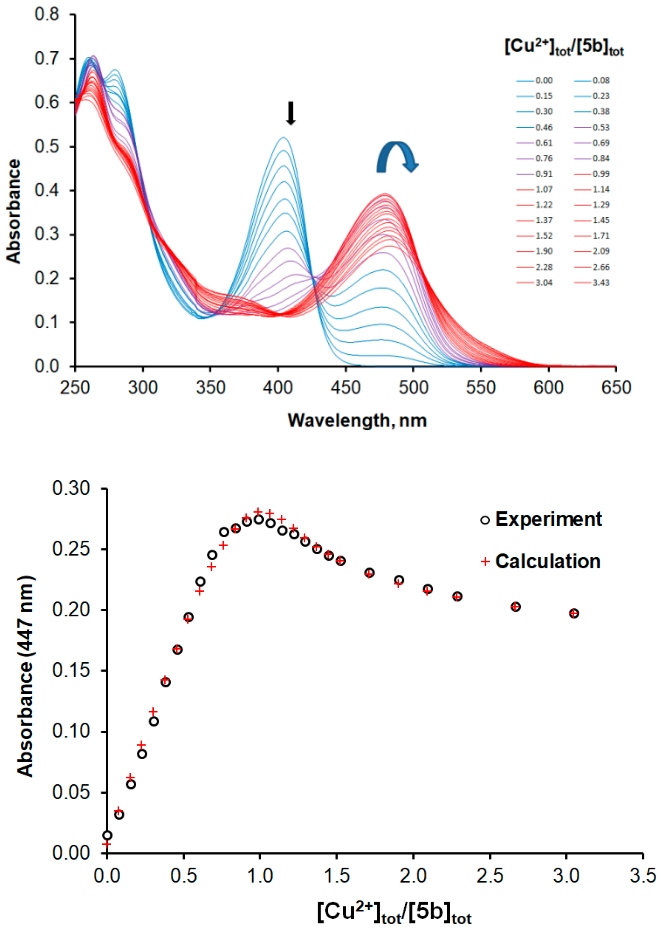

(**a**)

(**b**)

Figure S23. (**a**) Evolution of the UV–vis absorption spectrum of **5b** in acetonitrile ([**5b**] = 23.3 μM) upon addition of Cu(ClO4)2 (0–3 equiv.). (**b**) Changes of the absorbance as a function of the $[Cu^{2+}]_{tot}/[\mathbf{5b}]_{tot}$ ratio at λ =447 nm.

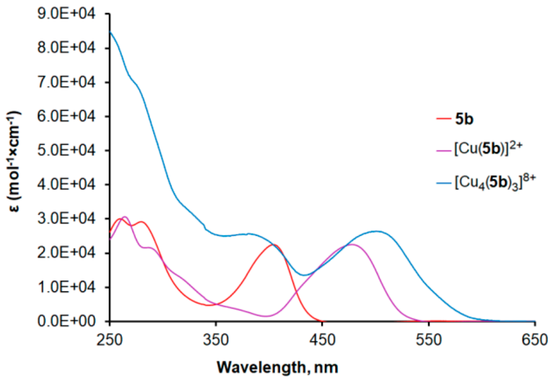

(a)

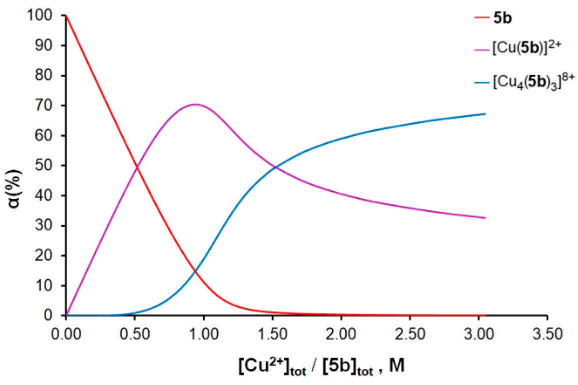

(b)

Figure S24. (**a**) UV–vis spectra of **5b**, $[Cu(\mathbf{5b})]^{2+}$ and $[Cu_4(\mathbf{5b})_3]^{8+}$ in acetonitrile calculated using HypSpec program.[2] (**b**) Species distribution diagram for the **5b**/Cu^{2+} system in acetonitrile calculated using HypSpec program.[2] Data were fit with HypSpec using the following model:

$Cu^{2+} + L \leftrightarrows [CuL]^{2+}$ (log β= 6.73(1))

$4Cu^{2+} + 3L \leftrightarrows [Cu_4L_3]^{8+}$ (log β= 34.7(1))

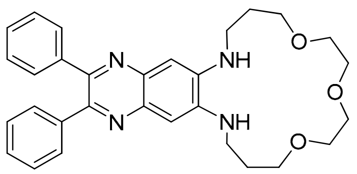

5c

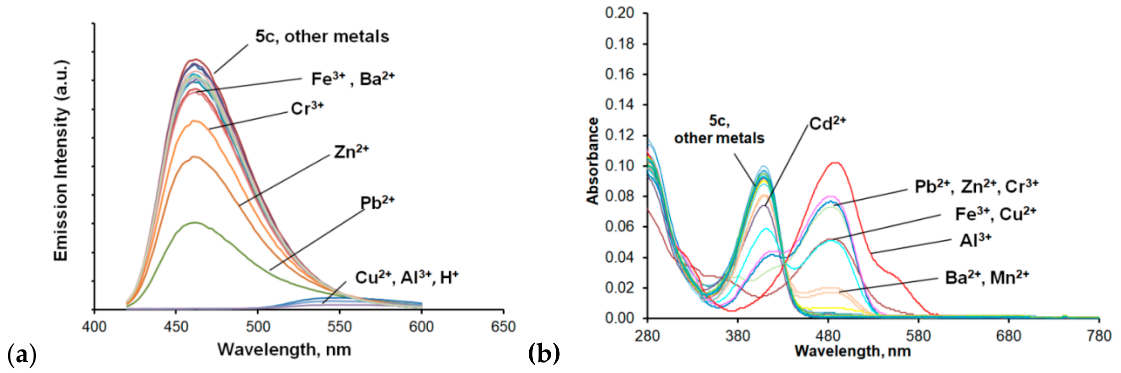

Figure S25. Luminescence (**a**) and absorption (**b**) spectra of **5c** in acetonitrile ([**5c**] = 4.2 μM, λ_{ex} = 409 nm) before and after addition of 5 equiv. of metal perchlorate salts.

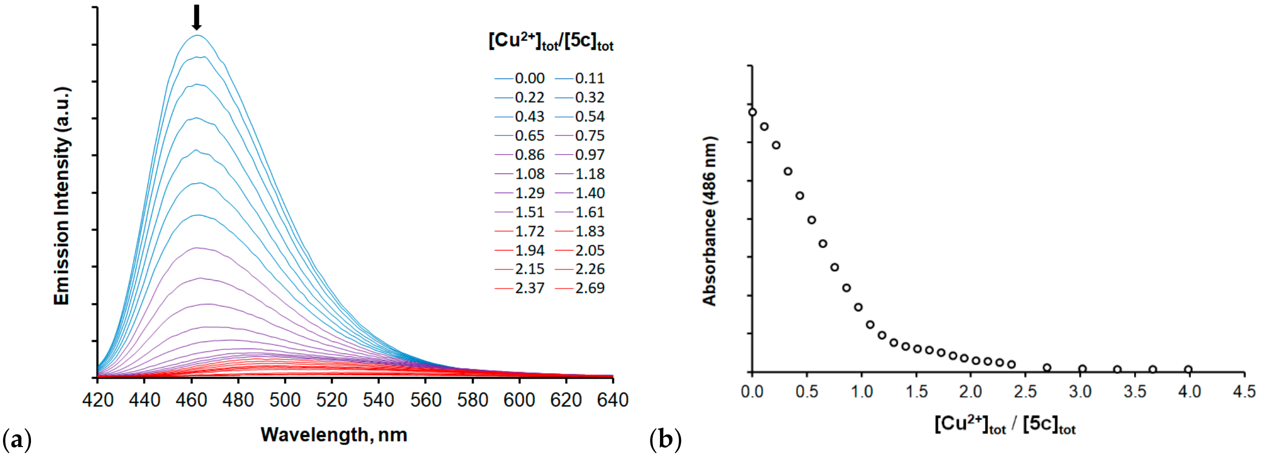

Figure S26. (**a**) Evolution of the emission spectrum of **5c** in acetonitrile ([**5c**]$_{tot}$ = 4.2 μM, λ_{ex} = 409 nm) upon addition of $Cu(ClO_4)_2$ (0–4 equiv.). (**b**) Changes of the emission intensity as a function of the $[Cu^{2+}]_{tot}/[\mathbf{5c}]_{tot}$ ratio at λ_{em} = 486 nm.

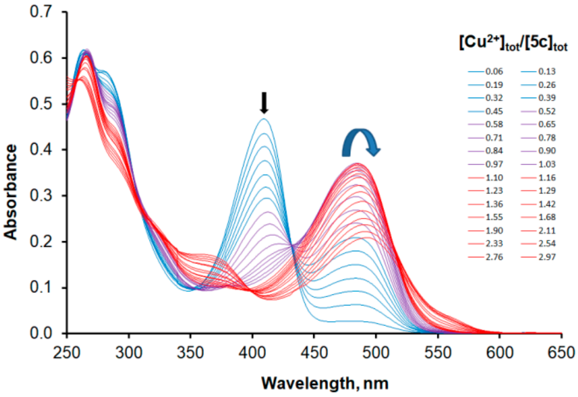

(**a**)

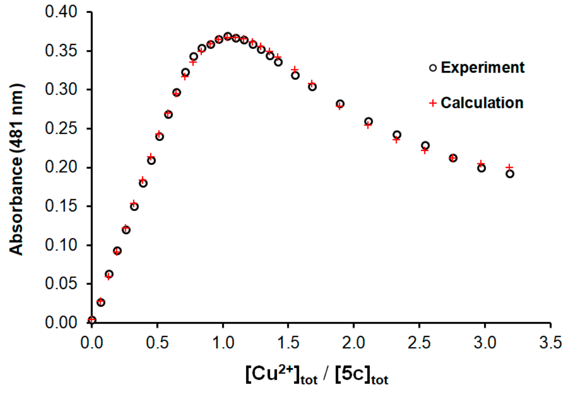

(**b**)

Figure S27. (**a**) Evolution of the UV–vis absorption spectrum of **5c** in acetonitrile ([**5c**] = 24.4 μM) upon addition of Cu(ClO4)2 (0–3 equiv.). (**b**) Changes of the absorbance as a function of the $[Cu^{2+}]_{tot}/[\mathbf{5c}]_{tot}$ ratio at λ =481 nm.

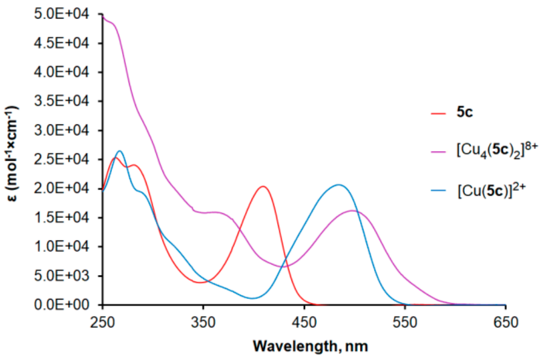

(**a**)

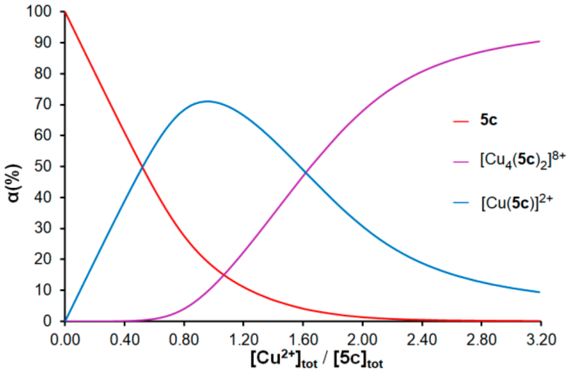

(**b**)

Figure S28. (**a**) UV–vis spectra of **5c**, $[Cu(\mathbf{5c})]^{2+}$ and $[Cu_4(\mathbf{5c})_2]^{8+}$ in acetonitrile calculated using HypSpec program.[2] (**b**) Species distribution diagram for the **5c**/Cu^{2+} system in acetonitrile calculated using HypSpec program.[2] Data were fit with HypSpec using the following model:

$Cu^{2+} + L \leftrightarrows [CuL]^{2+}$ (log β = 6.47(1))

$4Cu^{2+} + 2L \leftrightarrows [Cu_4L_2]^{8+}$ (log β = 28.3(3))

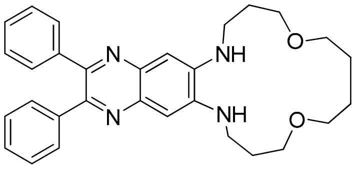

5d

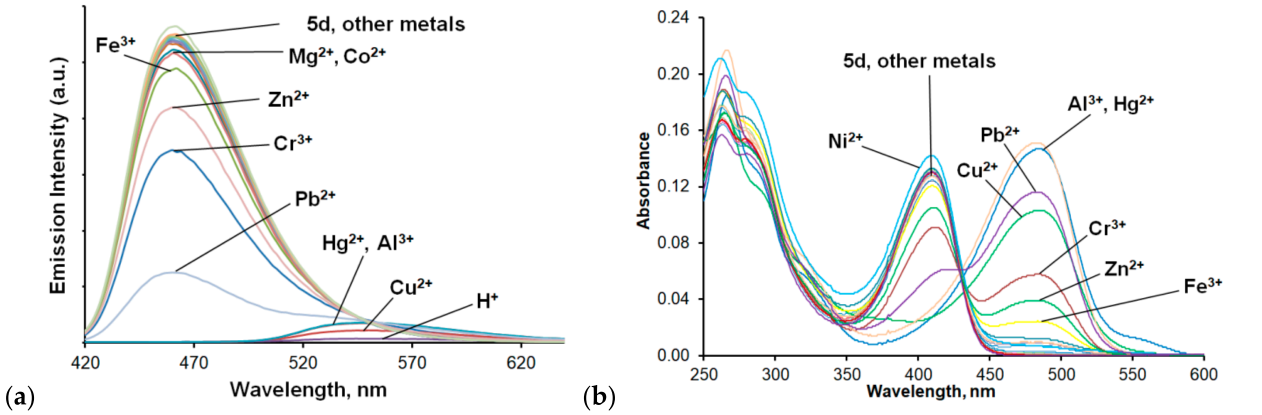

Figure S29. Luminescence (**a**) and absorption (**b**) spectra of **5d** in acetonitrile ([**5d**] = 4.8 μM, λ_{ex} = 410 nm) before and after addition of 5 equiv. of metal perchlorate salts.

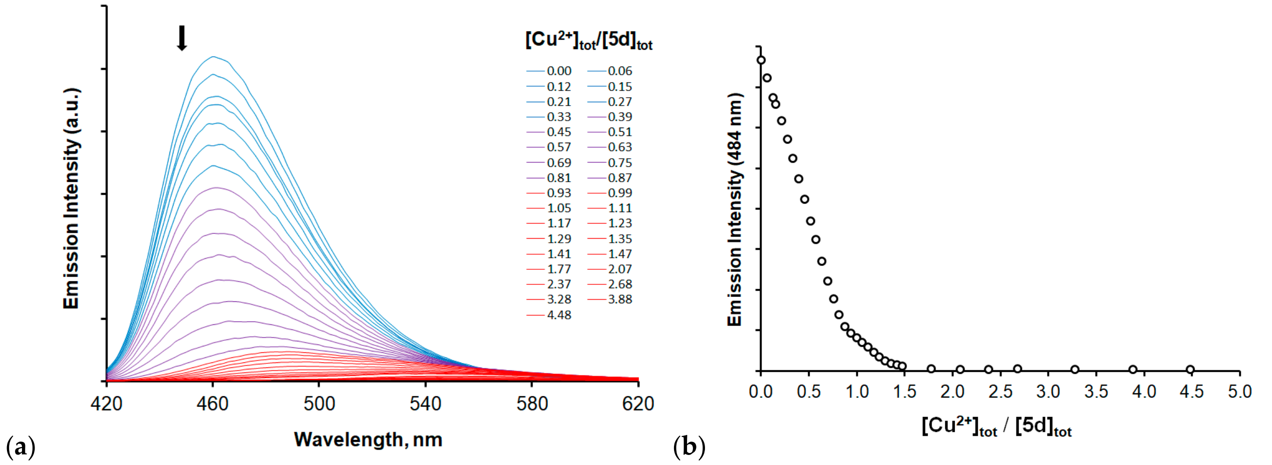

Figure S30. (**a**) Evolution of the emission spectrum of **5d** in acetonitrile ([**5d**]$_{tot}$ = 4.8 μM, λ_{ex} = 410 nm) upon addition of $Cu(ClO4)_2$ (0–4.5 equiv.). (**b**) Changes of the emission intensity as a function of the $[Cu^{2+}]_{tot}$/[**5d**]$_{tot}$ ratio at λ_{em} = 486 nm.

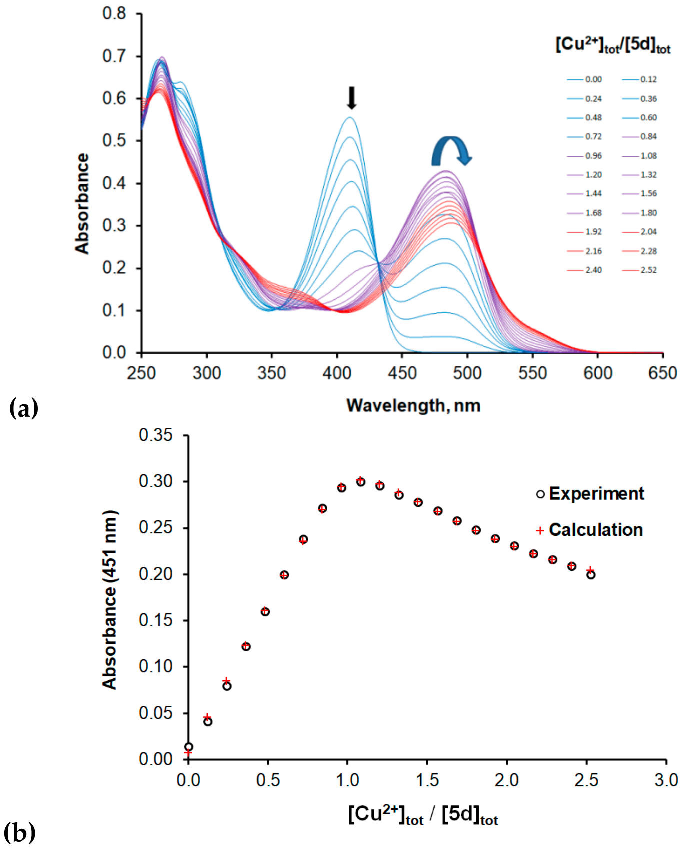

Figure S31. (**a**) Evolution of the UV–vis absorption spectrum of **5d** in acetonitrile ([**5d**] = 21.5 μM) upon addition of Cu(ClO4)2 (0–2.5 equiv.). (**b**) Changes of the absorbance as a function of the $[Cu^{2+}]_{tot}$/$[\mathbf{5d}]_{tot}$ ratio at λ =451 nm.

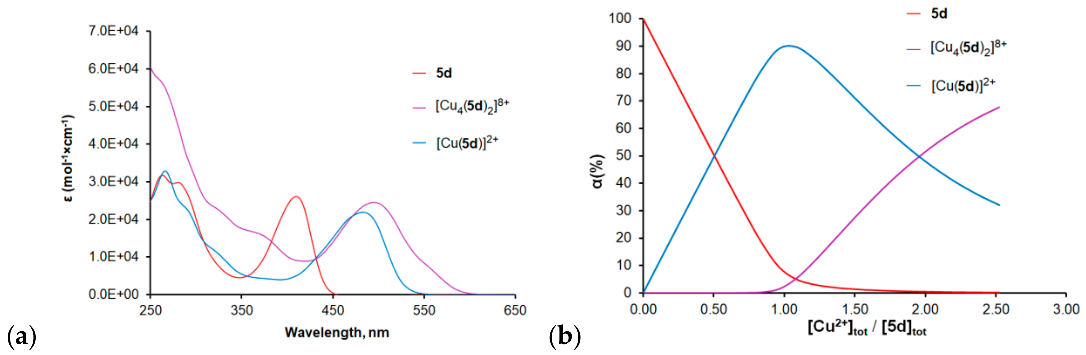

Figure S32. (**a**) UV–vis spectra of **5d**, $[Cu(\mathbf{5d})]^{2+}$ and $[Cu_4(\mathbf{5d})_2]^{8+}$ in acetonitrile calculated using HypSpec program.[2] (**b**) Species distribution diagram for the **5d**/Cu^{2+} system in acetonitrile calculated using HypSpec program.[2] Data were fit with HypSpec using the following model:

$Cu^{2+} + L \leftrightarrows [CuL]^{2+}$ (log β = 7.01(1))

$4Cu^{2+} + 2L \leftrightarrows [Cu_4L_2]^{8+}$ (log β = 28.7(1))

4. Protonation Studies of 5c and 5e

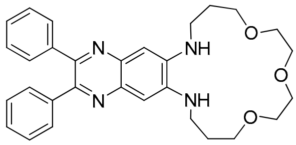

5c

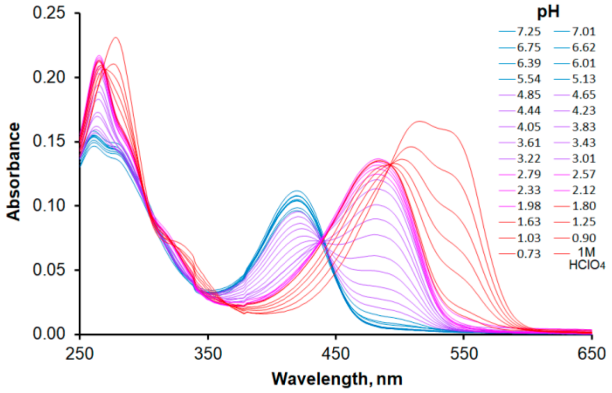

Figure S33. Spectrophotometric titration of **5c** as a function of pH ([**5c**]$_{tot}$ = 8.4 μM, 1 vol% MeOH, I = 0.1 M $NaClO_4$, pH = 0 – 7.3).

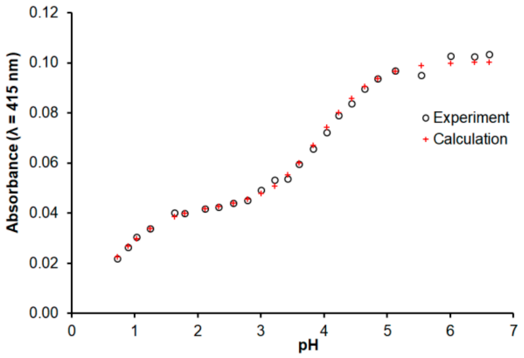

(**a**)

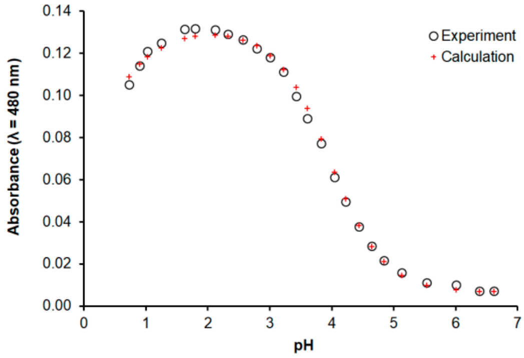

(**b**)

Figure S34. Absorbance changes with pH at λ = 415 nm (**a**) and λ = 480 nm (**b**) for **5c**.

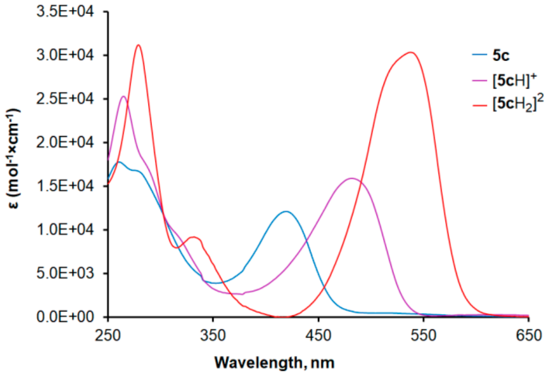

(**a**)

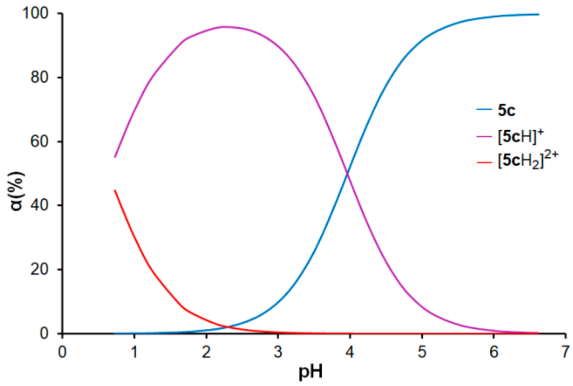

(**b**)

Figure S35. (**a**) UV–vis spectra of **5c**, [**5c**H]$^{+}$ and [**5c**H_2]$^{2+}$ in water calculated using HypSpec program.[2] (**b**) Species distribution diagram for the **5c** $\rightleftarrows$ [**5c**H]$^{+}$ $\rightleftarrows$ [**5c**H_2]$^{2+}$ system in water calculated using HypSpec program.[2]

pK_1 = 4.10(5); pK_2 = 0.6(1).

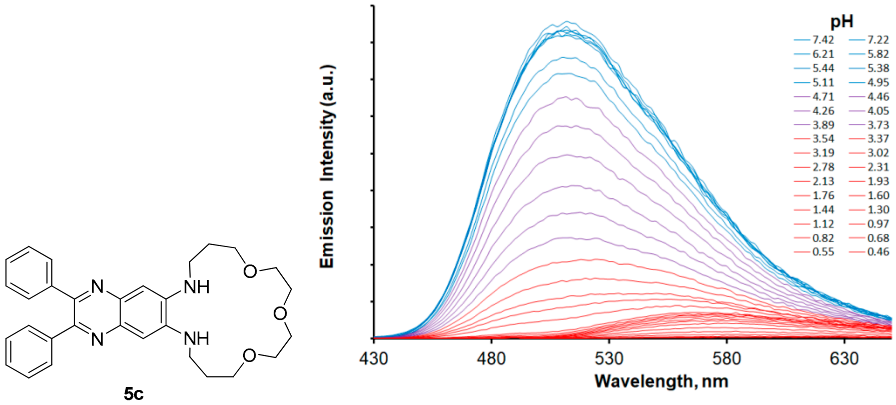

5c

Figure S36. Fluorimetric titration of **5c** as a function of pH ([**5c**]$_{tot}$ = 4.2 μM, 0.5 vol% MeOH, I = 0.1 M $NaClO_4$, λ_{ex} = 420 nm, pH = 0.5 – 7.4).

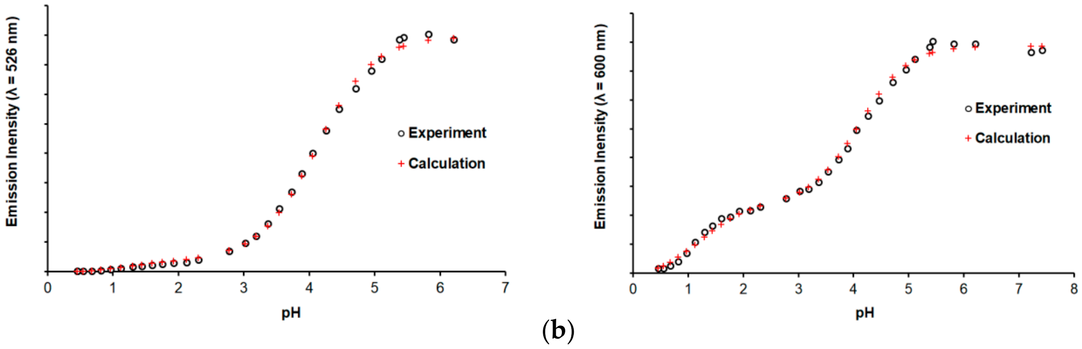

(**a**) (**b**)

Figure S37. Emission intensity changes with pH at λ = 526 nm (**a**) and λ = 600 nm (**b**) for **5c**.

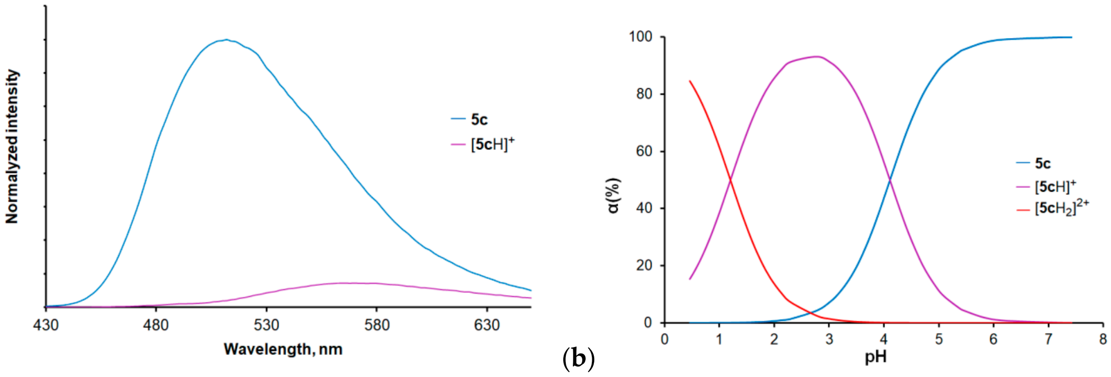

(**a**) (**b**)

Figure S38. (**a**) Emission spectra of **5c** and [**5c**H]$^{+}$ in water calculated using HypSpec program,[2] [**5c**H$_2$]$^{2+}$ was considered to be non-emissive; (**b**) Species distribution diagram for the **5c** $\rightleftarrows$ [**5c**H]$^{+}$ $\rightleftarrows$ [**5e**H$_2$]$^{2+}$system in water calculated using HypSpec program.[2]

pK_1 = 3.96(5); pK_2 = 1.20(5).

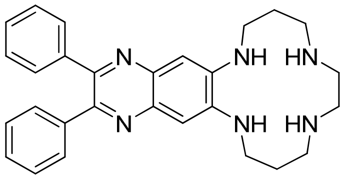

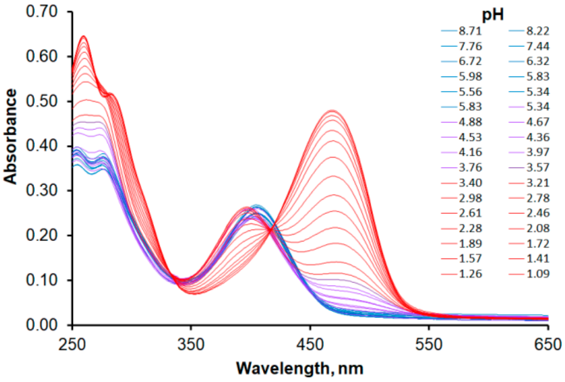

5e

Figure S39. Spectrophotometric titration of **5e** ([**5e**]$_{tot}$ = 31.1 μM, *I* = 0.1 M $NaClO_4$, pH = 1.1–8.8).

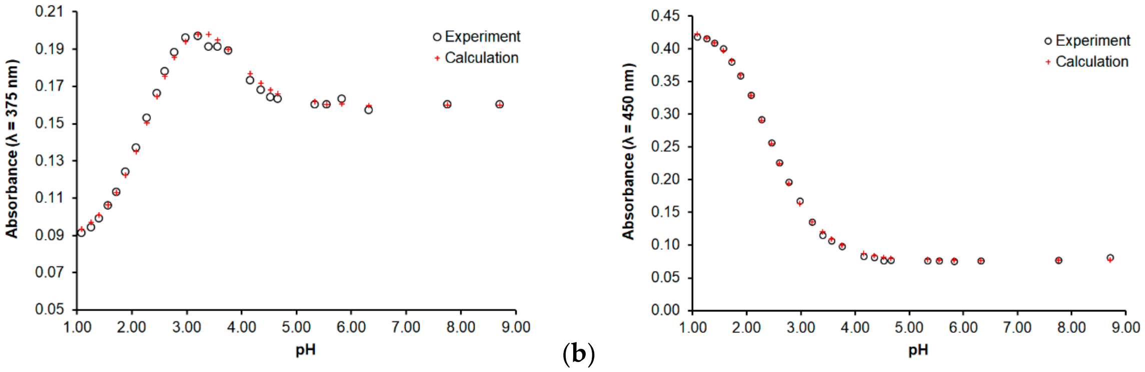

(**a**) (**b**)

Figure S40. Absorbance changes with pH at λ = 375 nm (**a**) and λ = 450 nm (**b**) for **5e**.

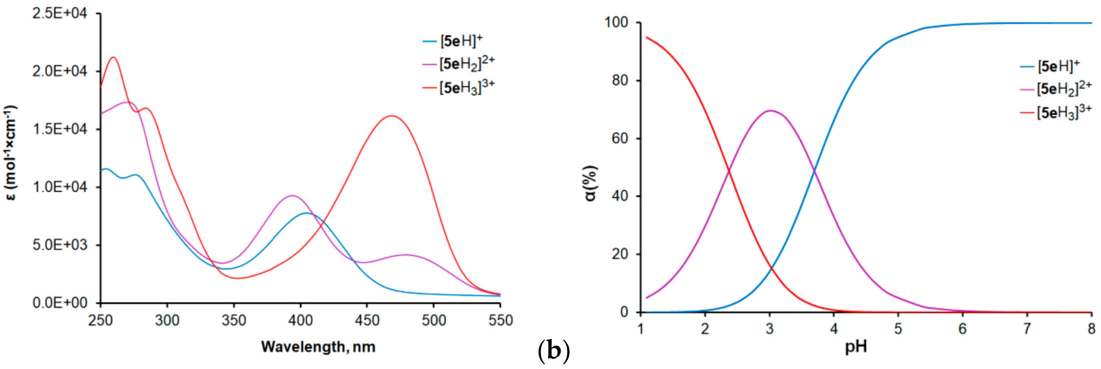

(**a**) (**b**)

Figure S41. (**a**) UV–vis spectra of [**5e**H]$^{+}$, [**5e**H_2]$^{2+}$ and [**5e**H_3]$^{3+}$ in water calculated using HypSpec program.[2] (**b**) Species distribution diagram for the [**5e**H]$^{+}$ $\rightleftharpoons$ [**5e**H_2]$^{2+}$ $\rightleftharpoons$ [**5e**H_3]$^{3+}$ system in water calculated using HypSpec program.[2]

pK_2 = 3.69(5); pK_3 = 2.36(5).

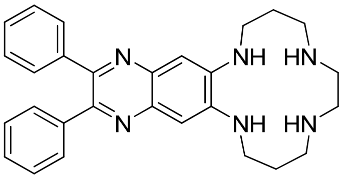

5e

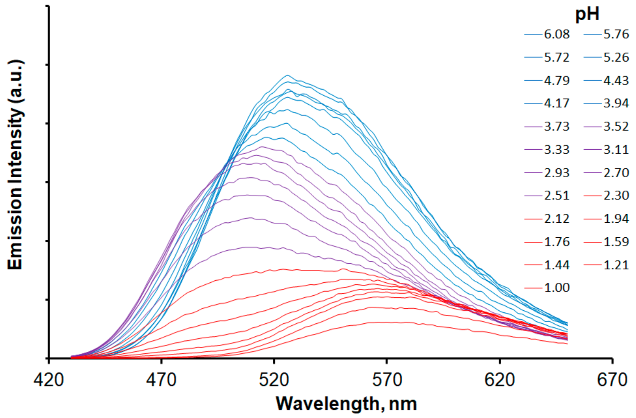

Figure S42. Fluorimetric titration of **5e** as a function of pH ([**5e**]$_{tot}$ = 8.04 μM, I = 0.1 M $NaClO_4$, λ_{ex} = 420 nm, pH = 1.0 – 6.1).

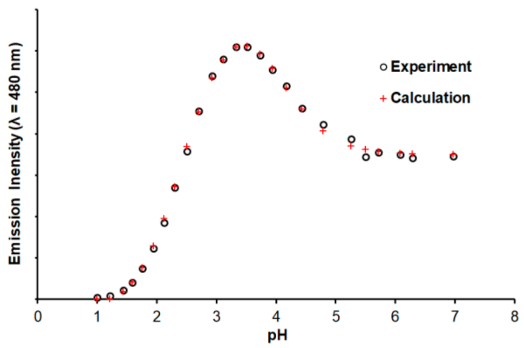

(**a**)

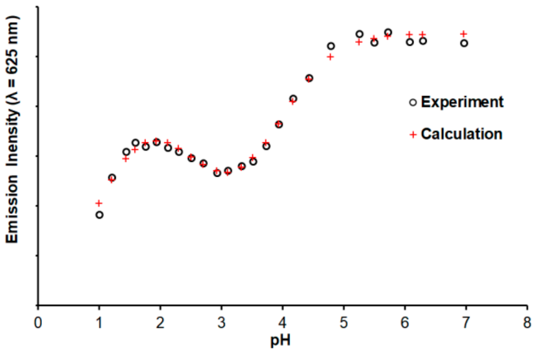

(**b**)

Figure S43. Emission intensity changes with pH at λ = 480 nm (**a**) and λ = 625 nm (**b**) for **5e**.

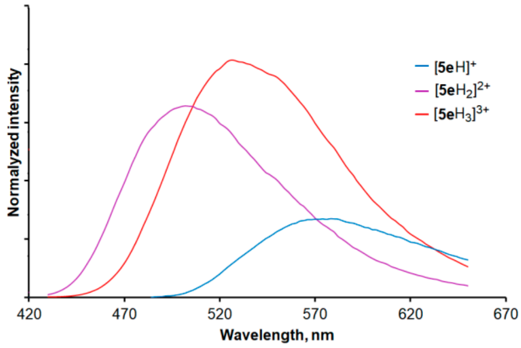

(**a**)

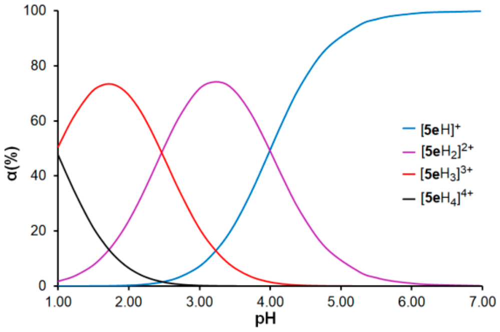

(**b**)

Figure S44. (**a**) Emission spectra of [**5e**H]$^{+}$, [**5e**H$_2$]$^{2+}$ and [**5e**H$_3$]$^{3+}$ in water calculated using HypSpec program,[2] [**5e**H$_4$]$^{4+}$ was considered to be non-emissive; (**b**) Species distribution diagram for the [**5e**H]$^{+}$ $\rightleftarrows$ [**5e**H$_2$]$^{2+}$ $\rightleftarrows$ [**5e**H$_3$]$^{3+}$ $\rightleftarrows$ [**5e**H$_4$]$^{4+}$system in water calculated using HypSpec program.[2]

pK_1 = 4.01(5); pK_2 = 2.46(5); pK_3 = 1.0(1).

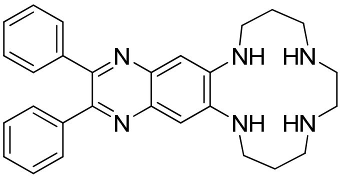

5e

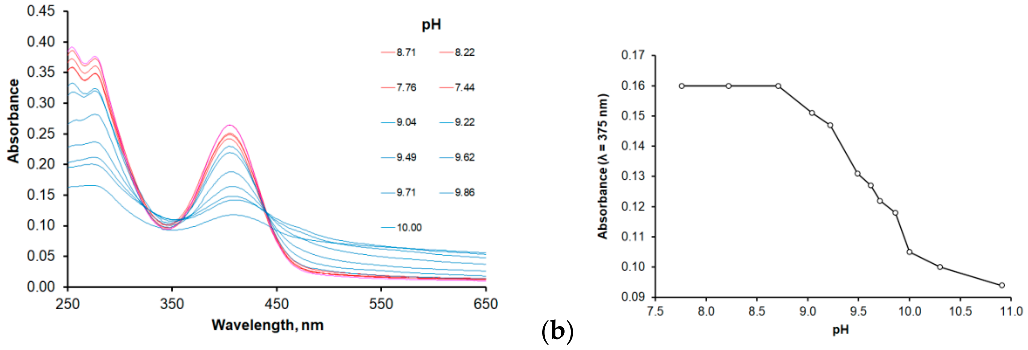

(**a**) (**b**)

Figure S45. (**a**) UV-vis spectra of **5e** solution ([**5e**]$_{tot}$ = 31.1 μM, I = 0.1 M $NaClO_4$, pH = 8.7–10.0); (**b**) Absorbance changes with pH at λ = 375 nm. Formation of precipitate is observed in the cell.

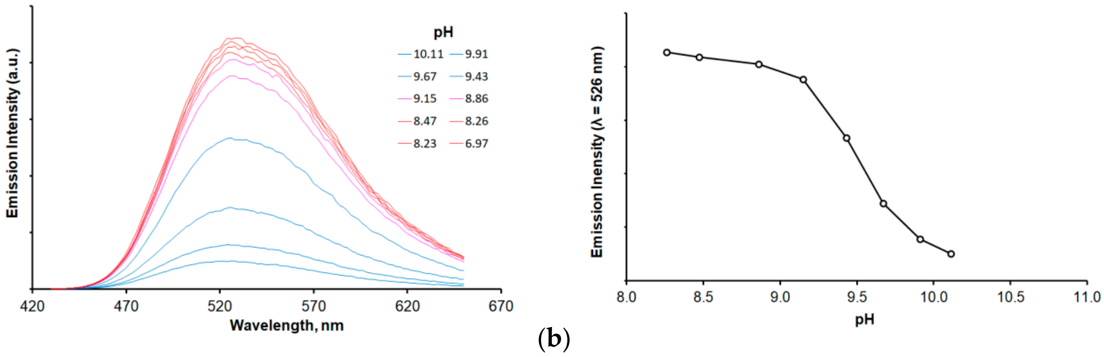

(**a**) (**b**)

Figure S46. (**a**) Luminescence spectra spectra of **5e** solution ([**5e**]$_{tot}$ = 8.04 μM, I = 0.1 M $NaClO_4$, pH = 7.0–10.1); (**b**) Emission intensity changes with pH at λ = 526 nm. Formation of precipitate is observed in the cell.

5. NMR Spectra of New Compounds.

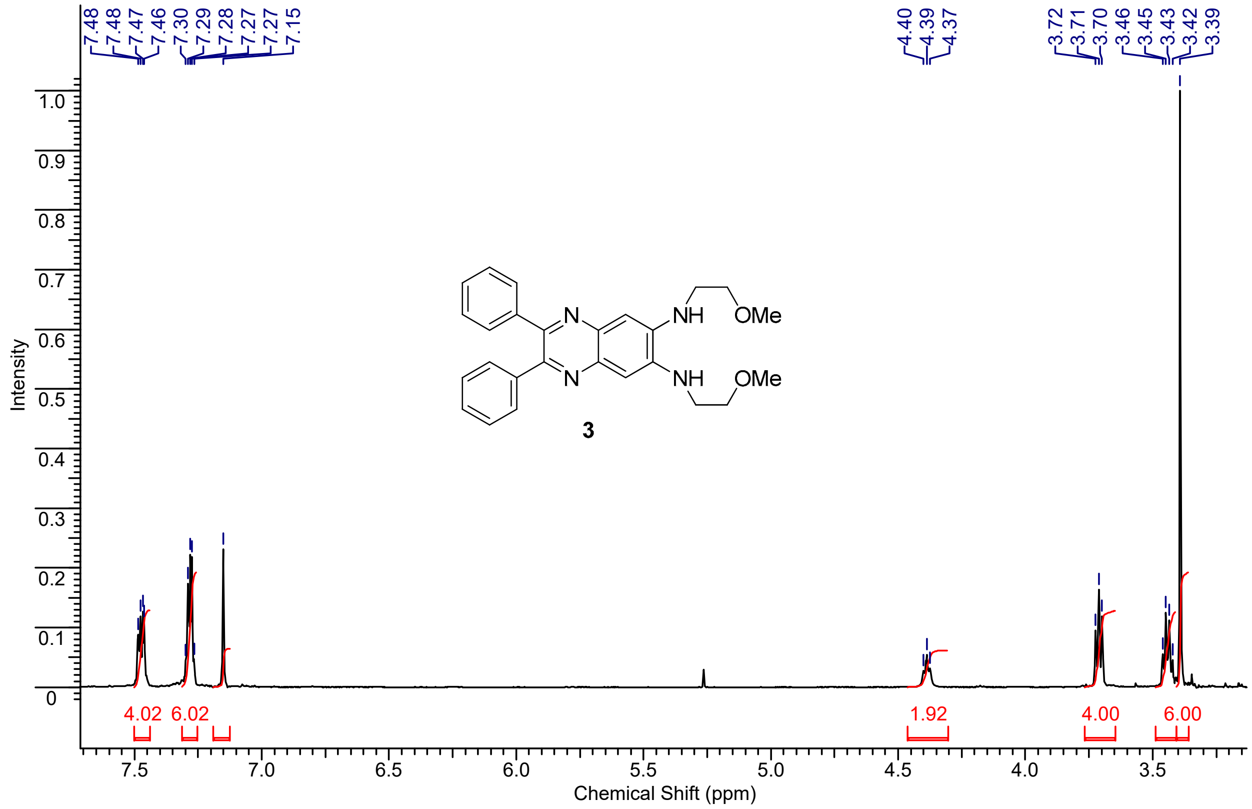

Figure S47. ^{1}H NMR spectrum of the compound **3** ($CDCl_3$, 400 MHz, 300 K).

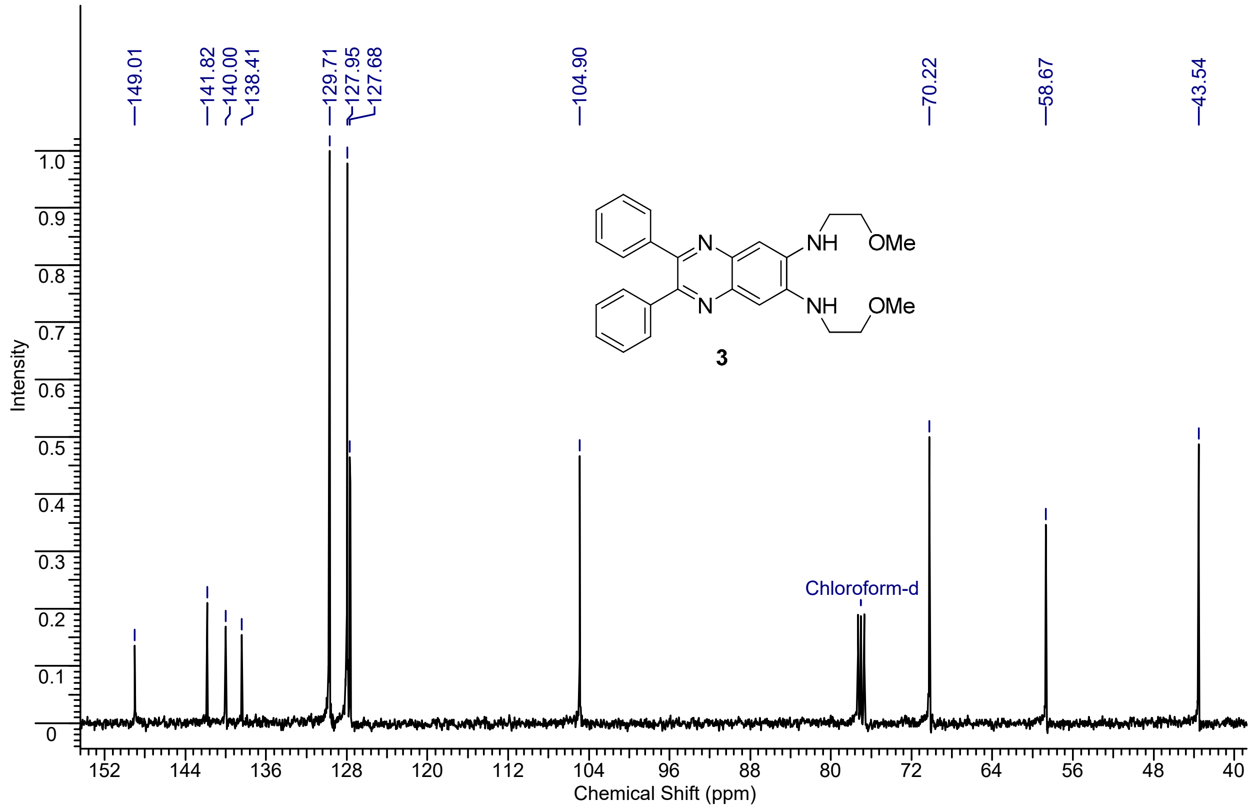

Figure S48. ^{13}C NMR spectrum of the compound **3** ($CDCl_3$, 100.6 MHz, 300 K).

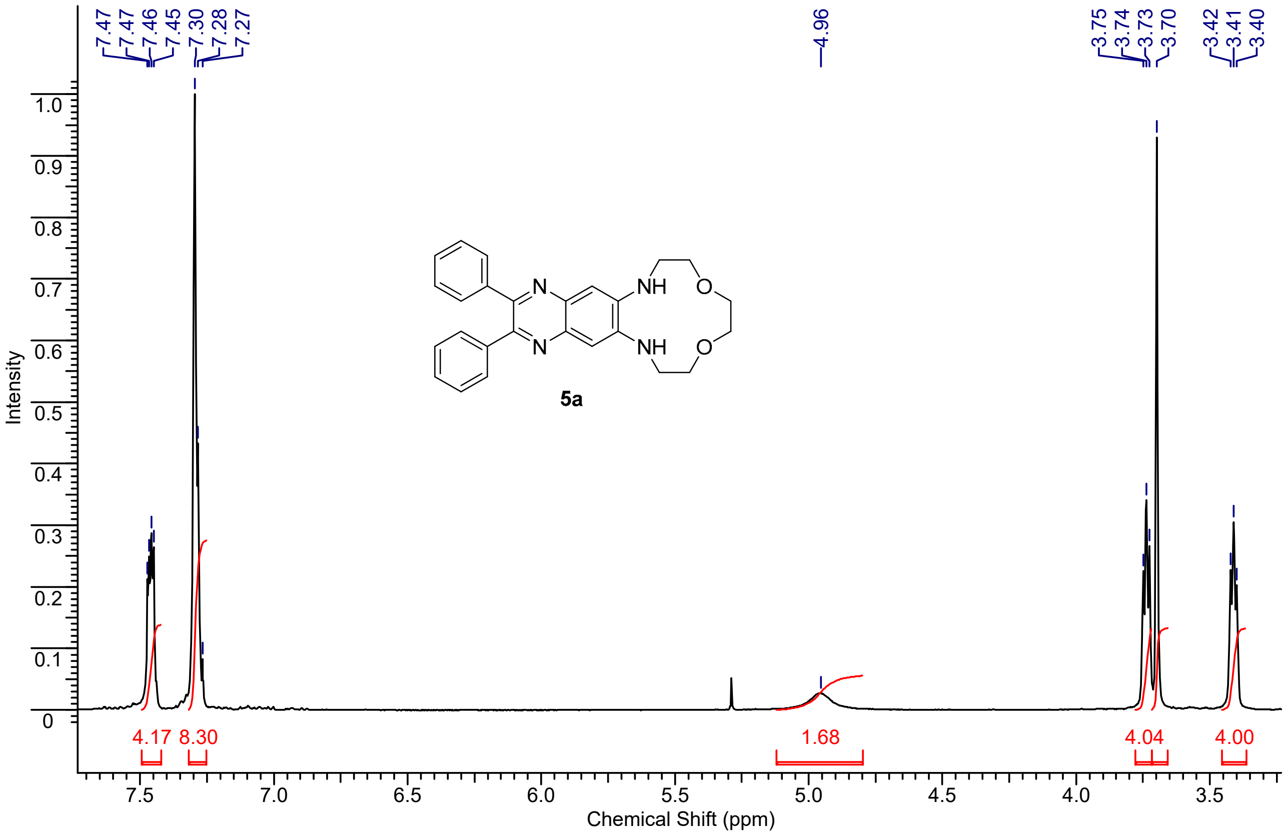

Figure S49. ^{1}H NMR spectrum of the compound **5a** ($CDCl_3$, 400 MHz, 300 K).

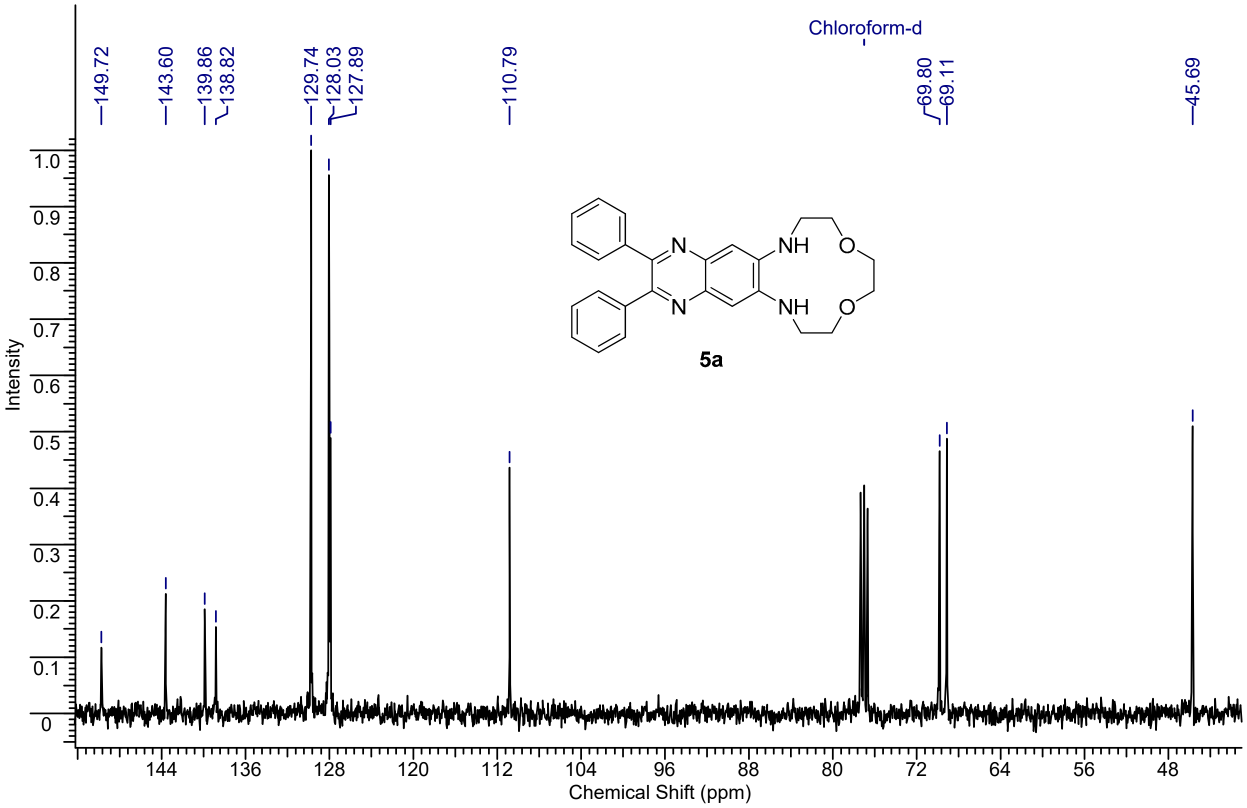

Figure S50. ^{13}C NMR spectrum of the compound **5a** ($CDCl_3$, 100.6 MHz, 300 K).

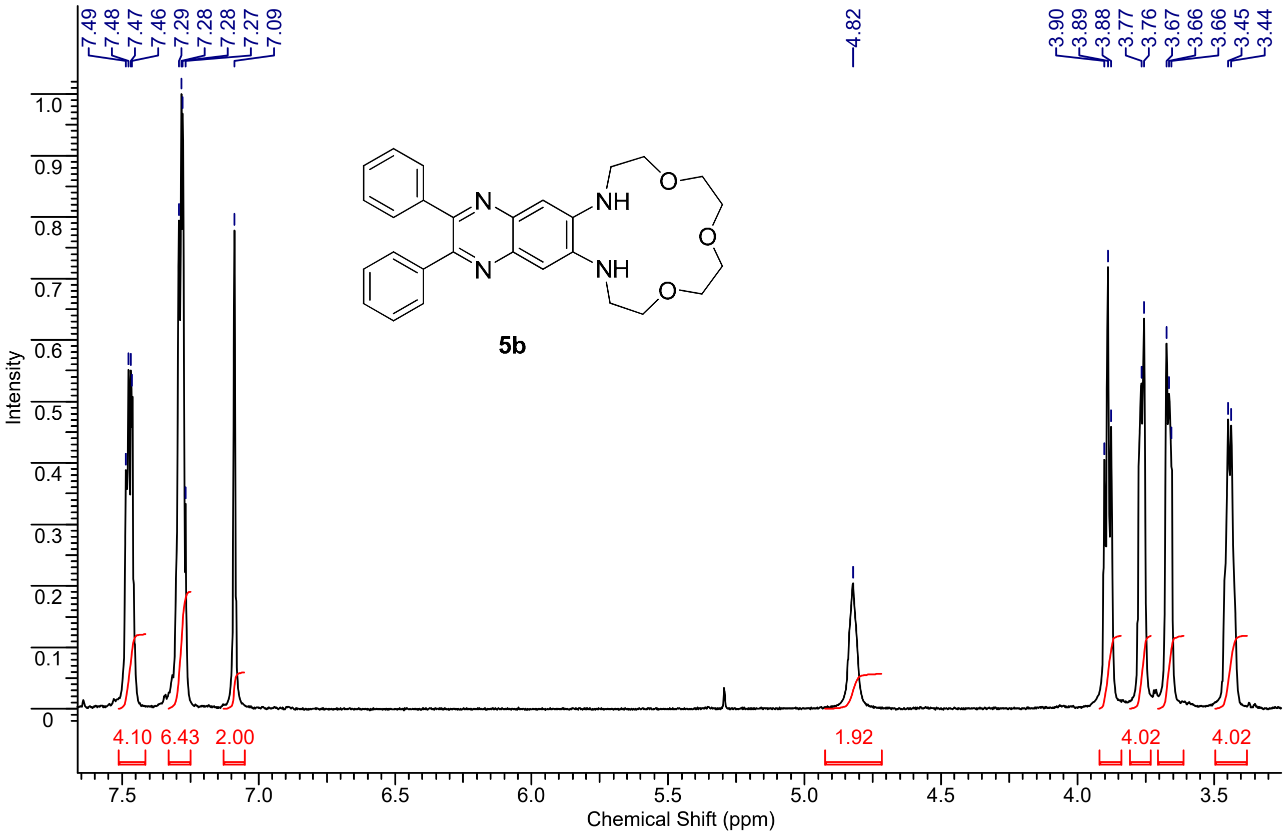

Figure S51.^{1}H NMR spectrum of the compound **5b** ($CDCl_3$, 400 MHz, 300 K).

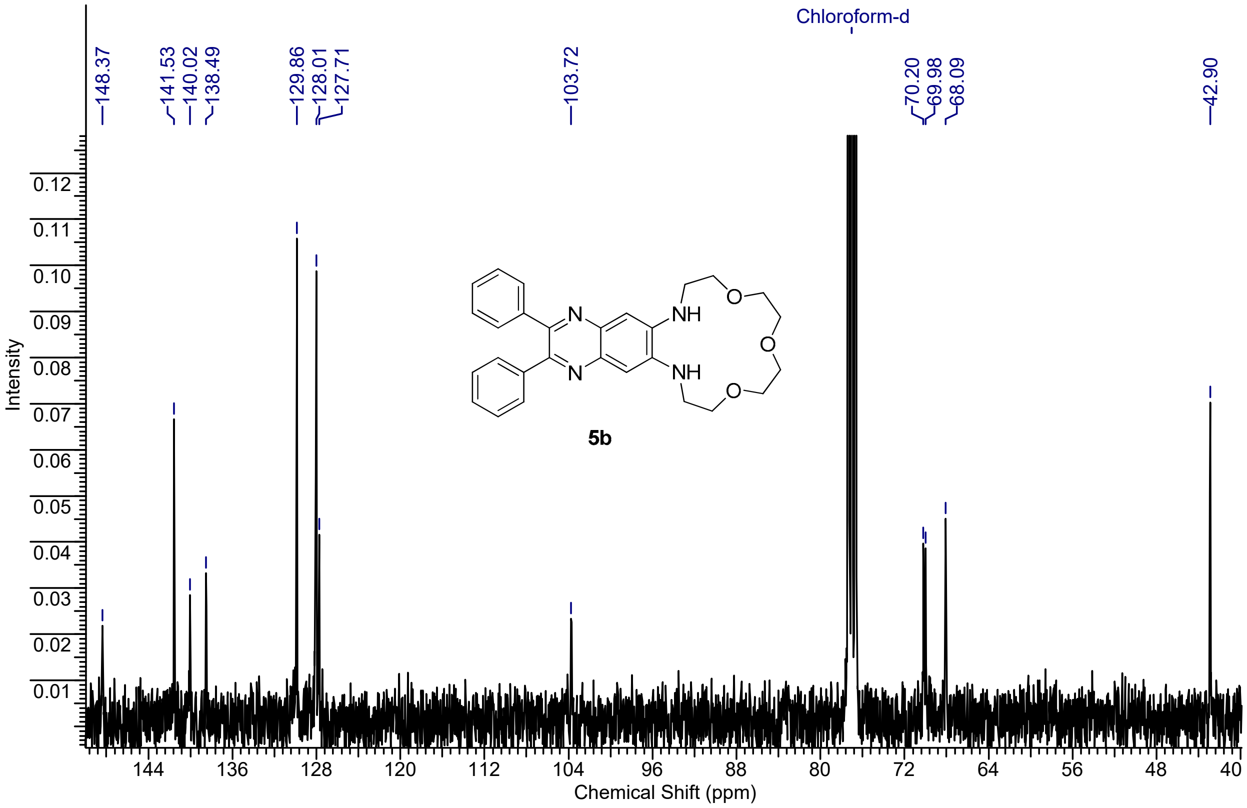

Figure S52. ^{13}C NMR spectrum of the compound **5b** ($CDCl_3$, 100.6 MHz, 300 K).

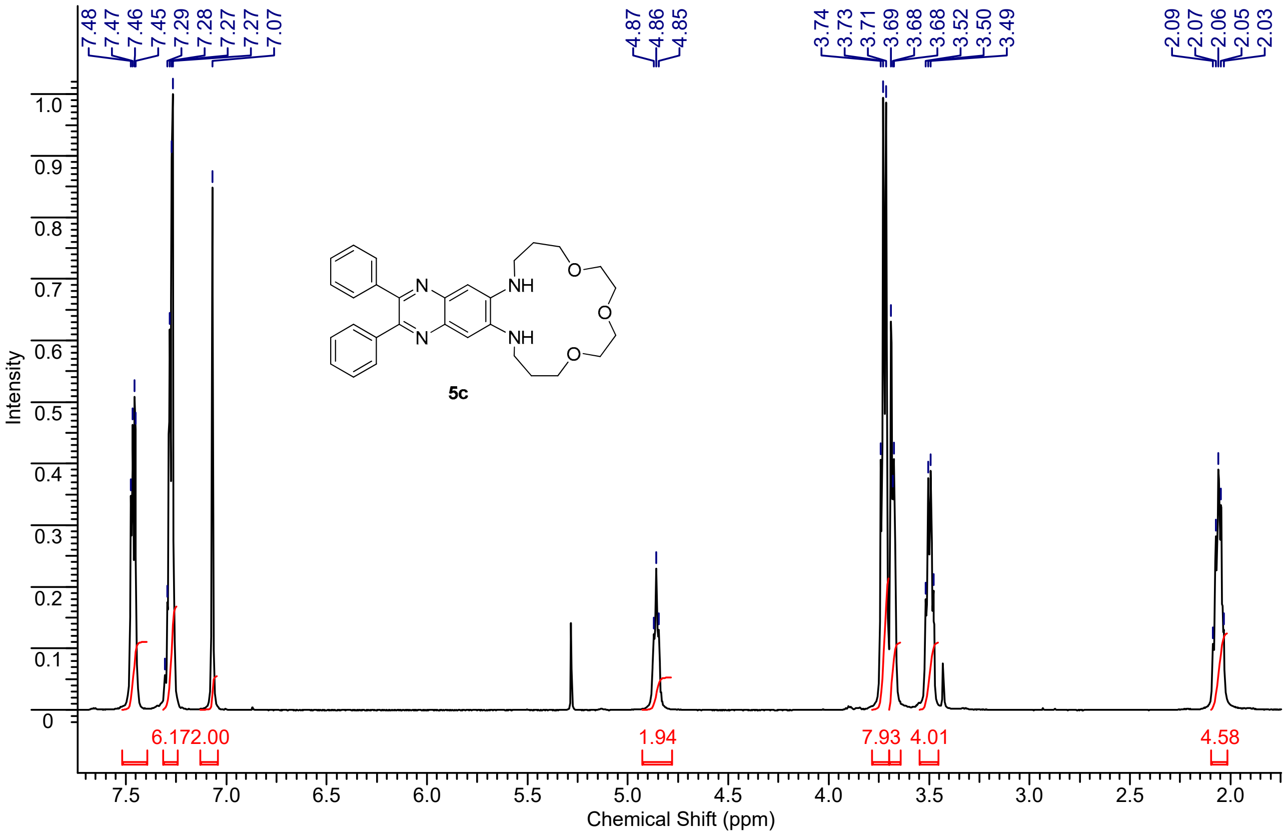

Figure S53. ^{1}H NMR spectrum of the compound **5c** ($CDCl_3$, 400 MHz, 300 K).

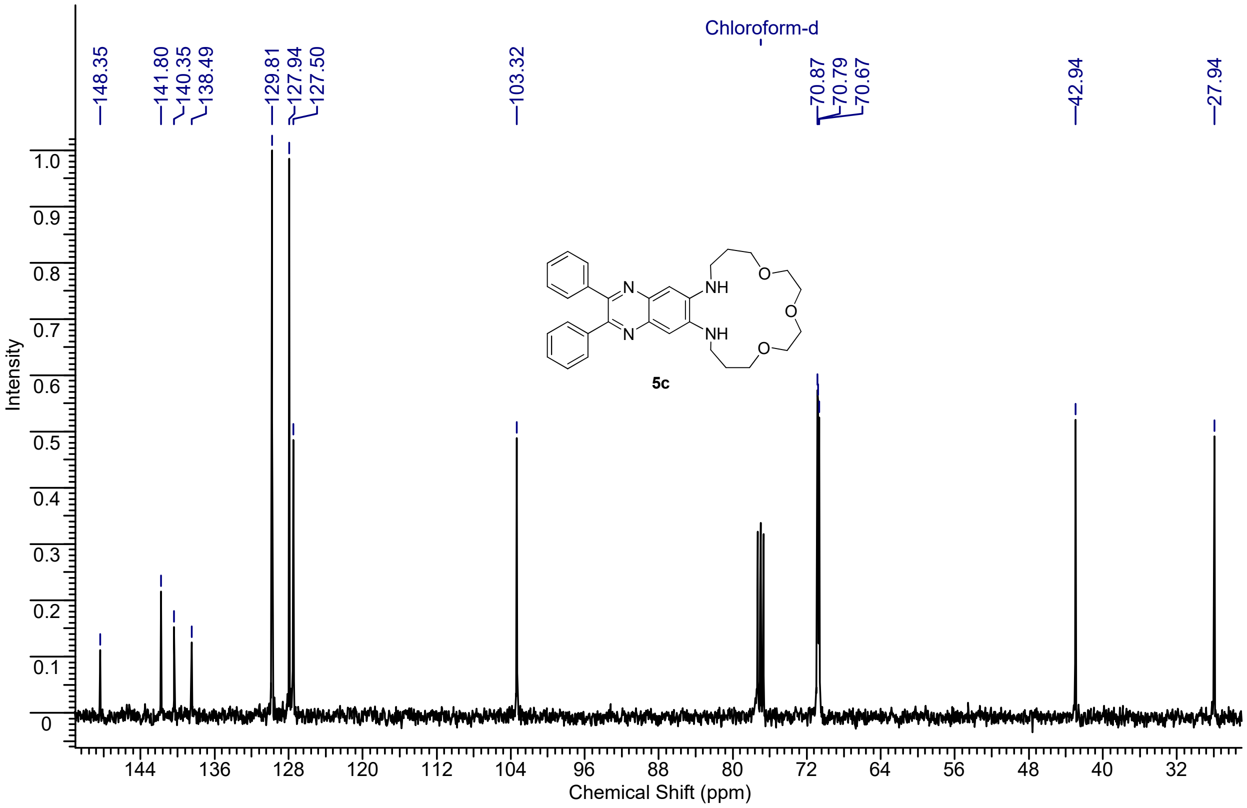

Figure S54. ^{13}C NMR spectrum of the compound **5c** ($CDCl_3$, 100.6 MHz, 300 K).

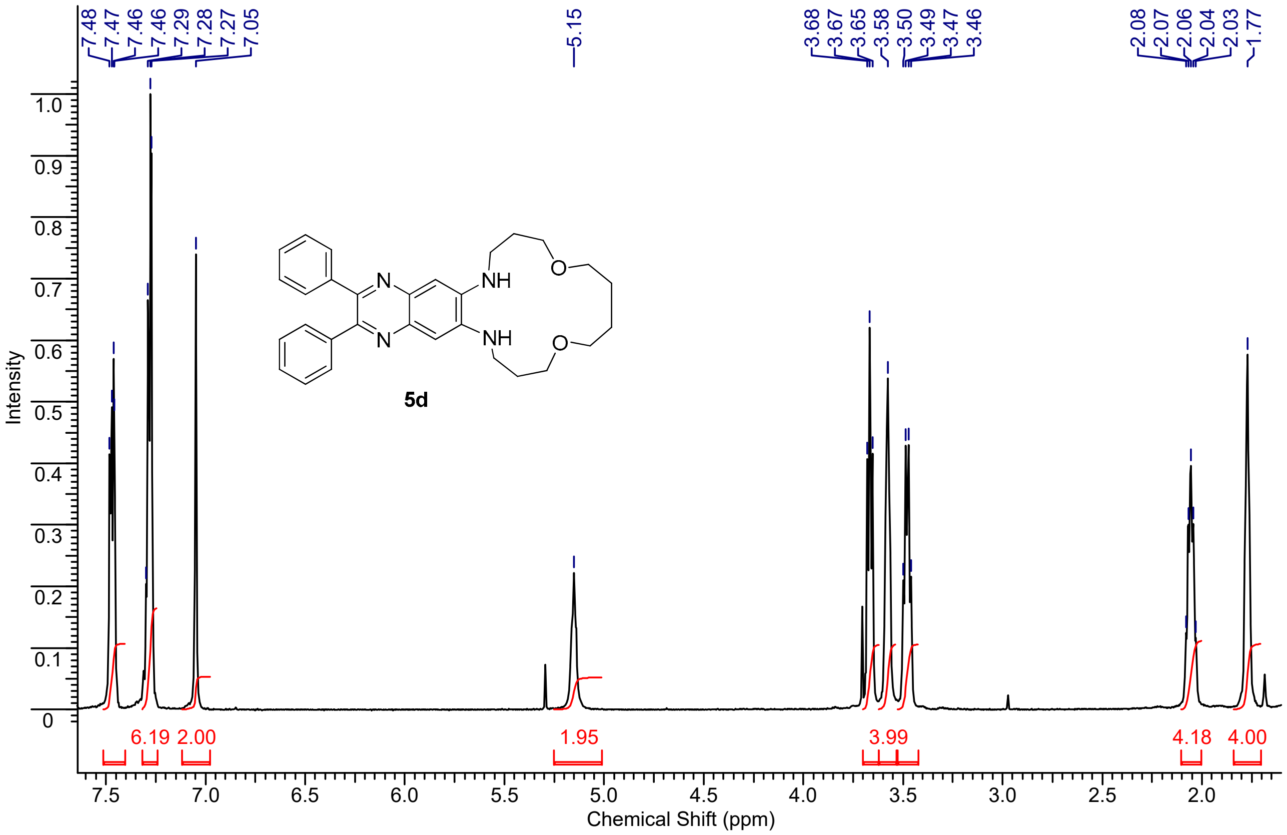

Figure S55. ^{1}H NMR spectrum of the compound **5d** ($CDCl_3$, 400 MHz, 300 K).

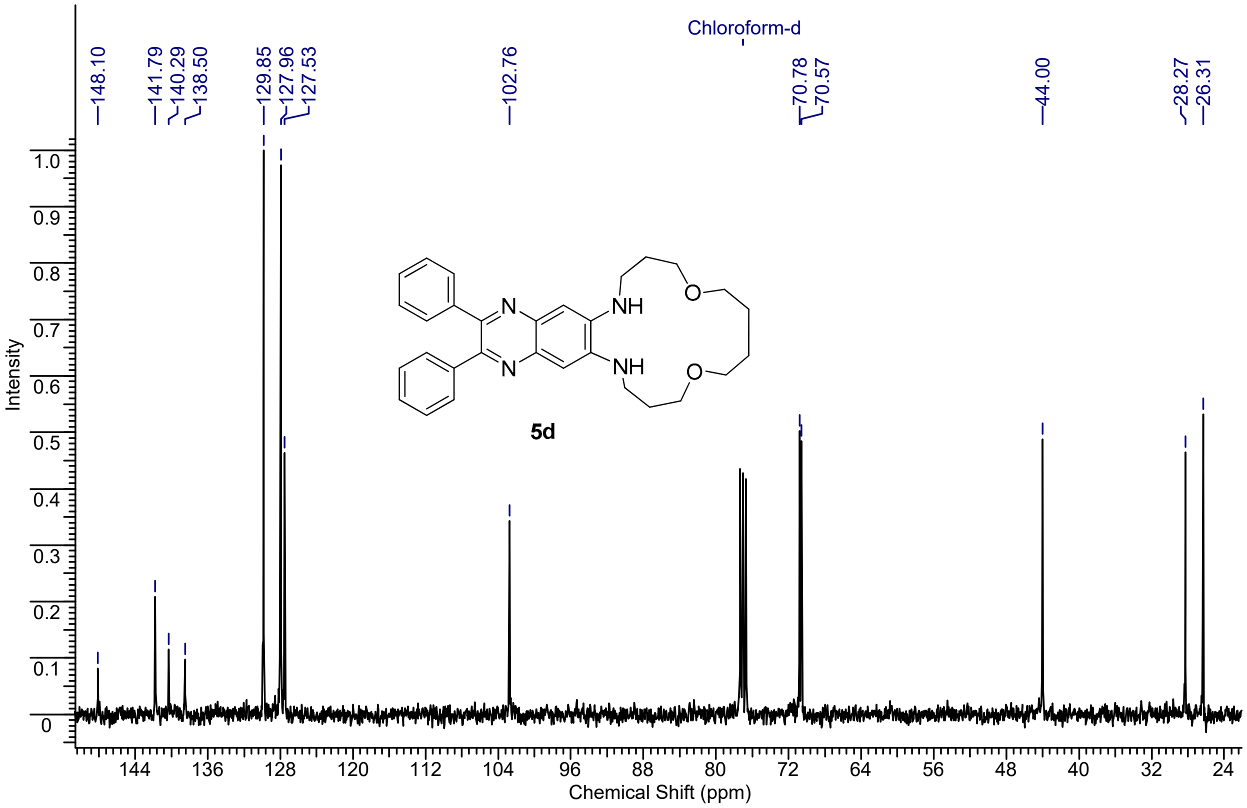

Figure S56. ^{13}C NMR spectrum of the compound **5d** ($CDCl_3$, 100.6 MHz, 300 K).

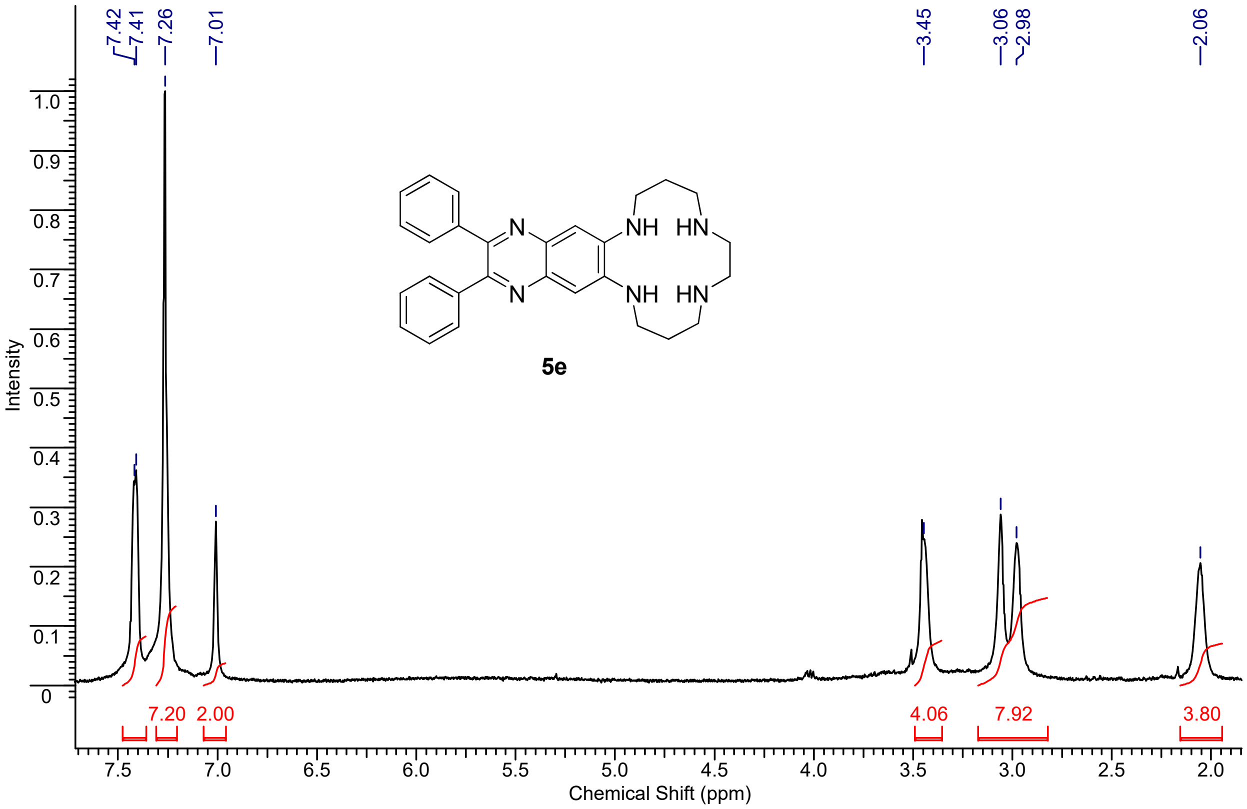

Figure S57. ^{1}H NMR spectrum of the compound **5e** ($CDCl_3/CD_3OD$, 5:1 v/v, 400 MHz, 300 K).

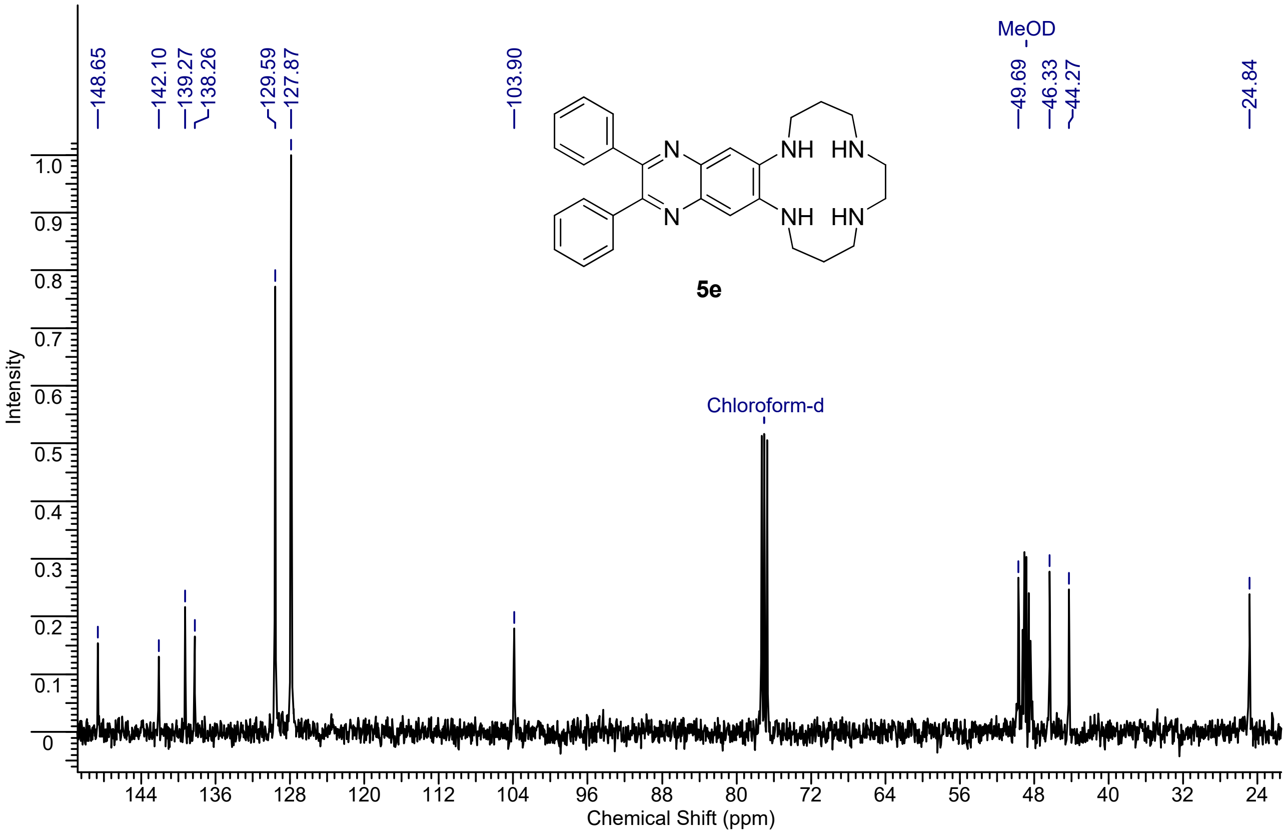

Figure S58. ^{13}C NMR spectrum of the compound **5e** ($CDCl_3/CD_3OD$, 5:1 v/v, 100.6 MHz, 300 K).

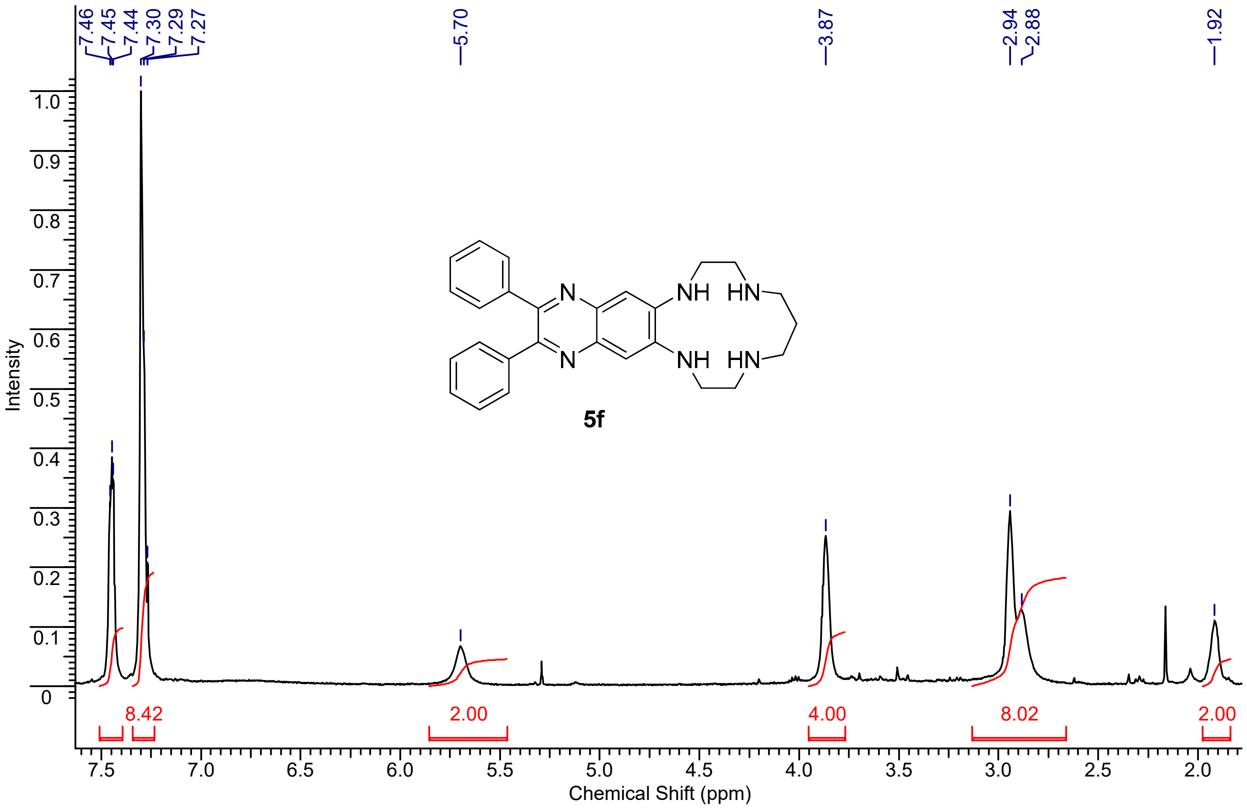

Figure S59. ^{1}H NMR spectrum of the compound **5f** ($CDCl_3/CD_3OD$, 5:1 v/v, 400 MHz, 300 K).

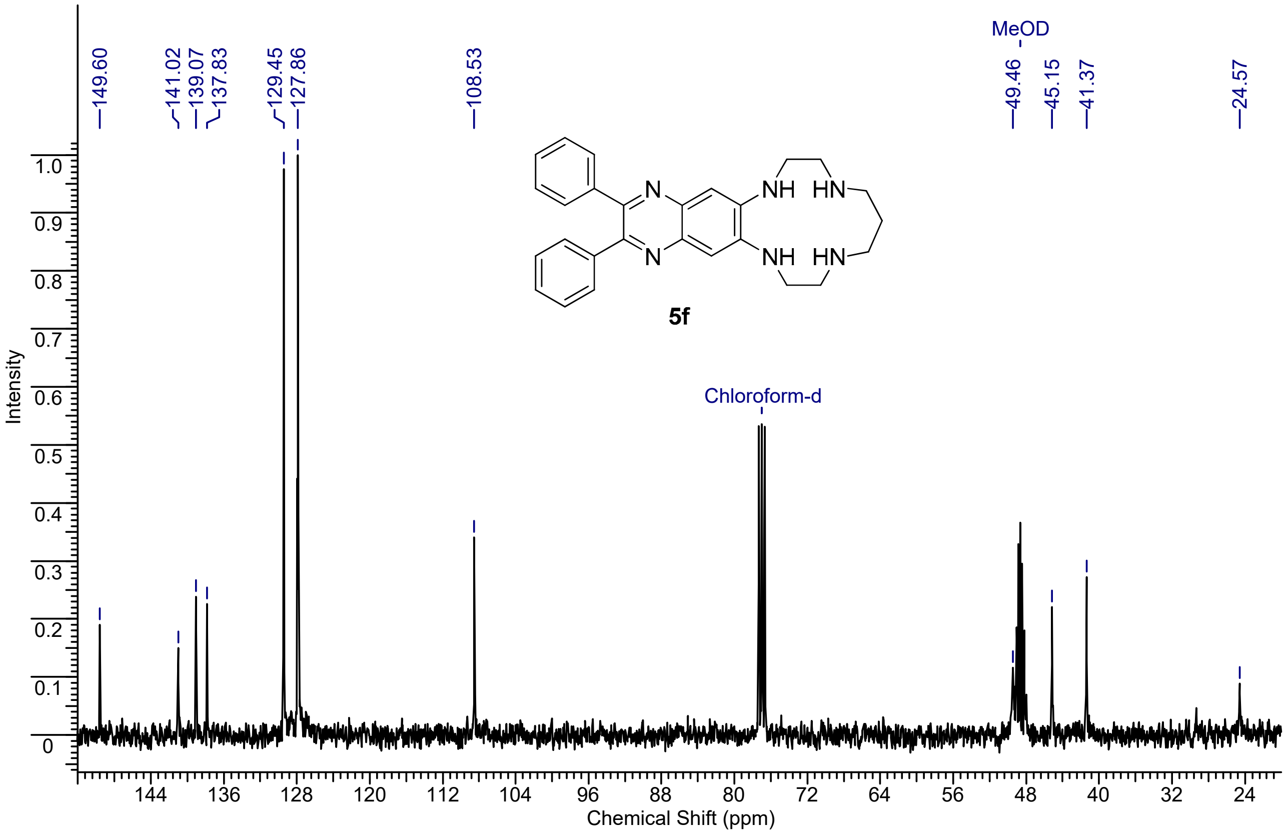

Figure S60. ^{13}C NMR spectrum of the compound **5f** ($CDCl_3/CD_3OD$, 5:1 v/v, 100.6 MHz, 300 K).

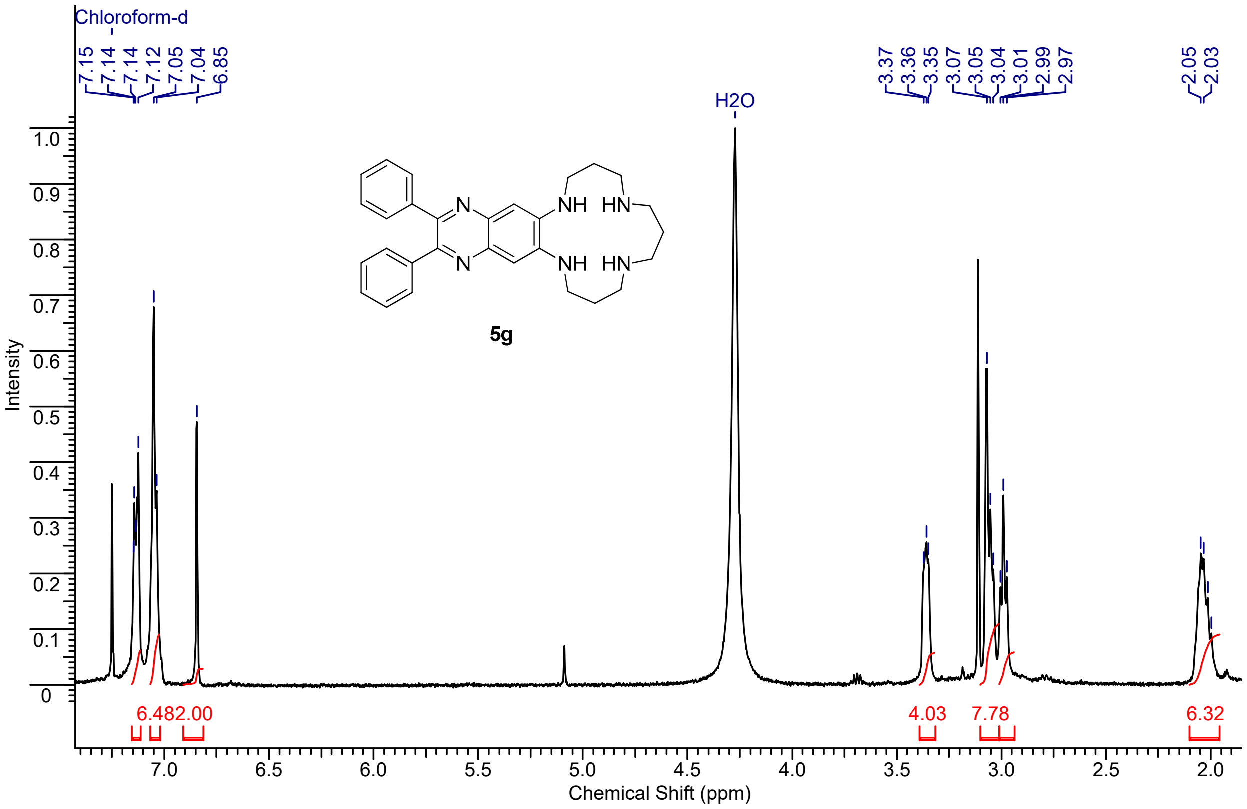

Figure S61. ^{1}H NMR spectrum of the compound **5g** ($CDCl_3/CD_3OD$, 5:1 v/v, 400 MHz, 300 K).

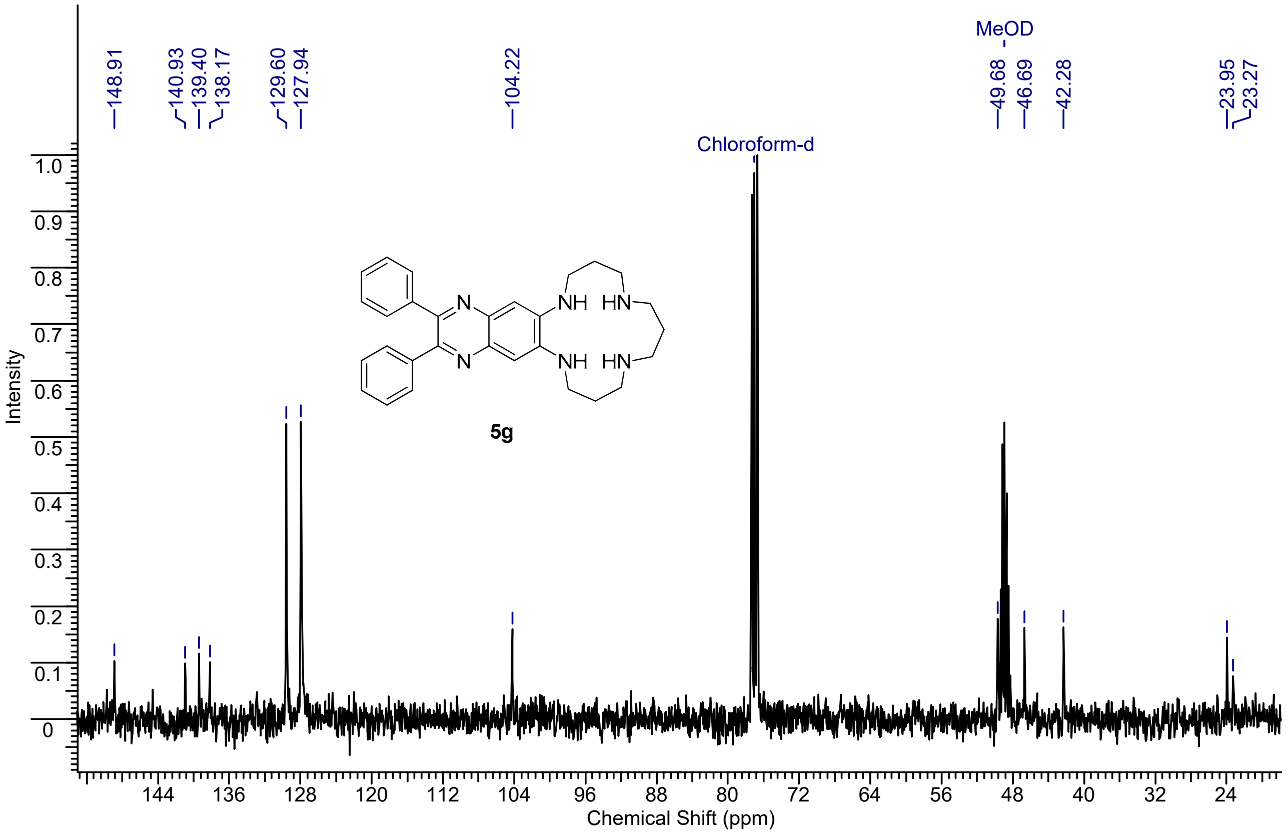

Figure S62. ^{13}C NMR spectrum of the compound **5g** ($CDCl_3/CD_3OD$, 5:1 v/v, 100.6 MHz, 300 K).

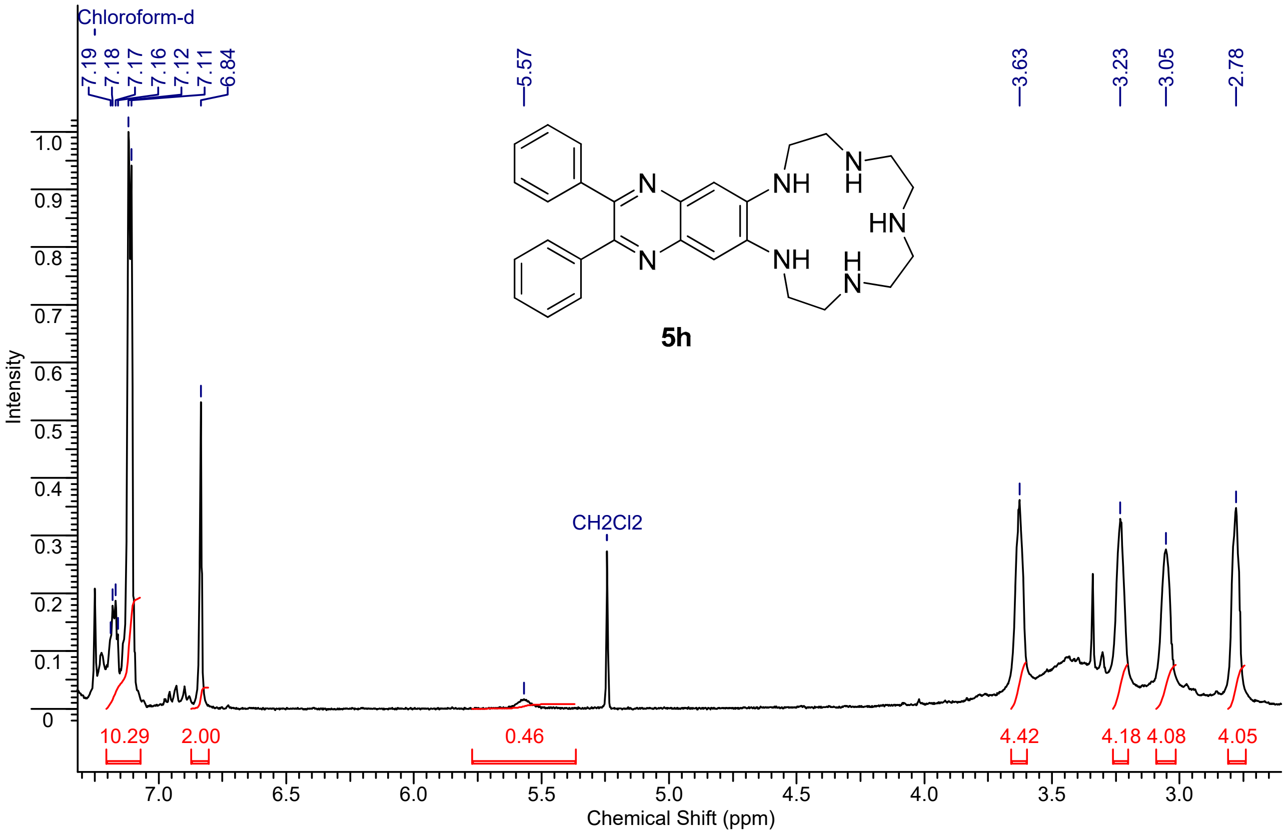

Figure S63. ^{1}H NMR spectrum of the compound **5h** ($CDCl_3/CD_3OD$, 10:1 v/v, 400 MHz, 300 K).

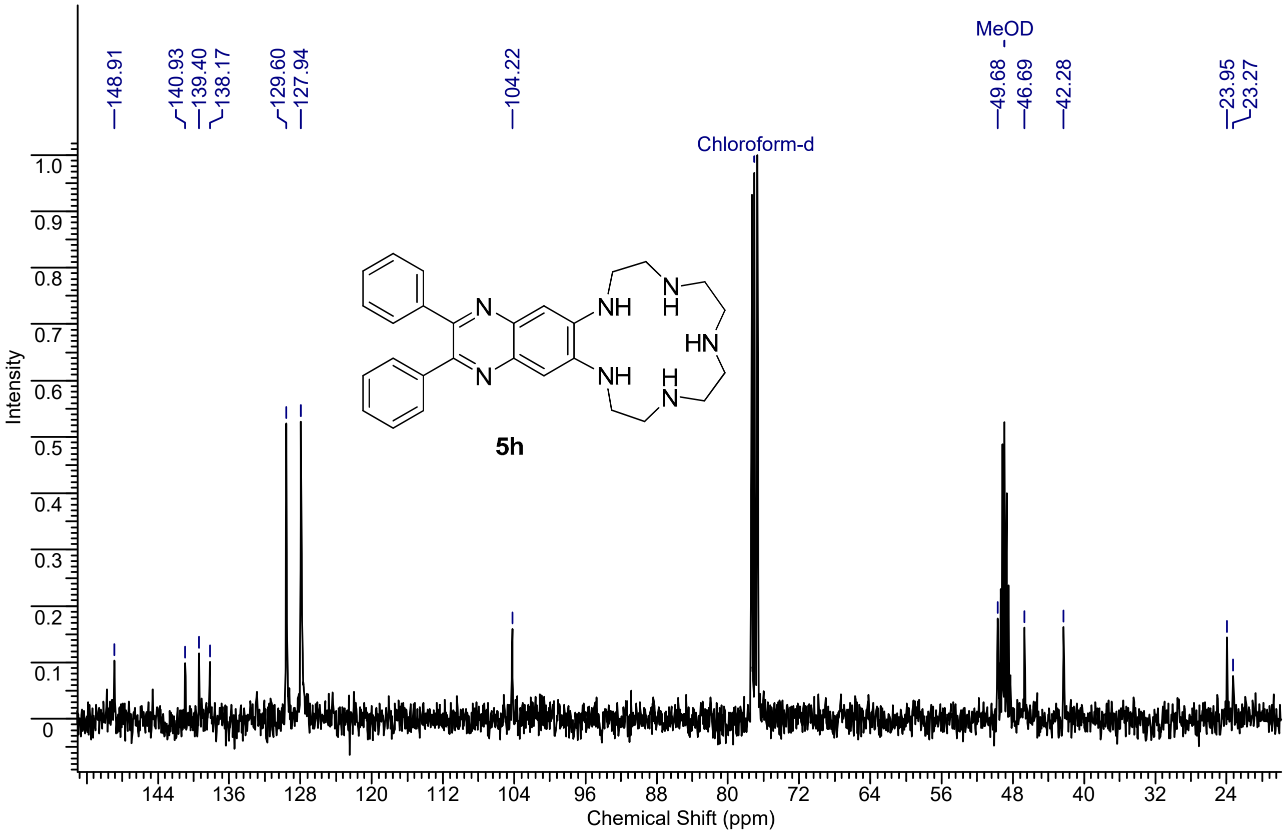

Figure S64. ^{13}C NMR of the compound **5h** ($CDCl_3/CD_3OD$, 10:1 v/v, 100.6 MHz, 300 K).

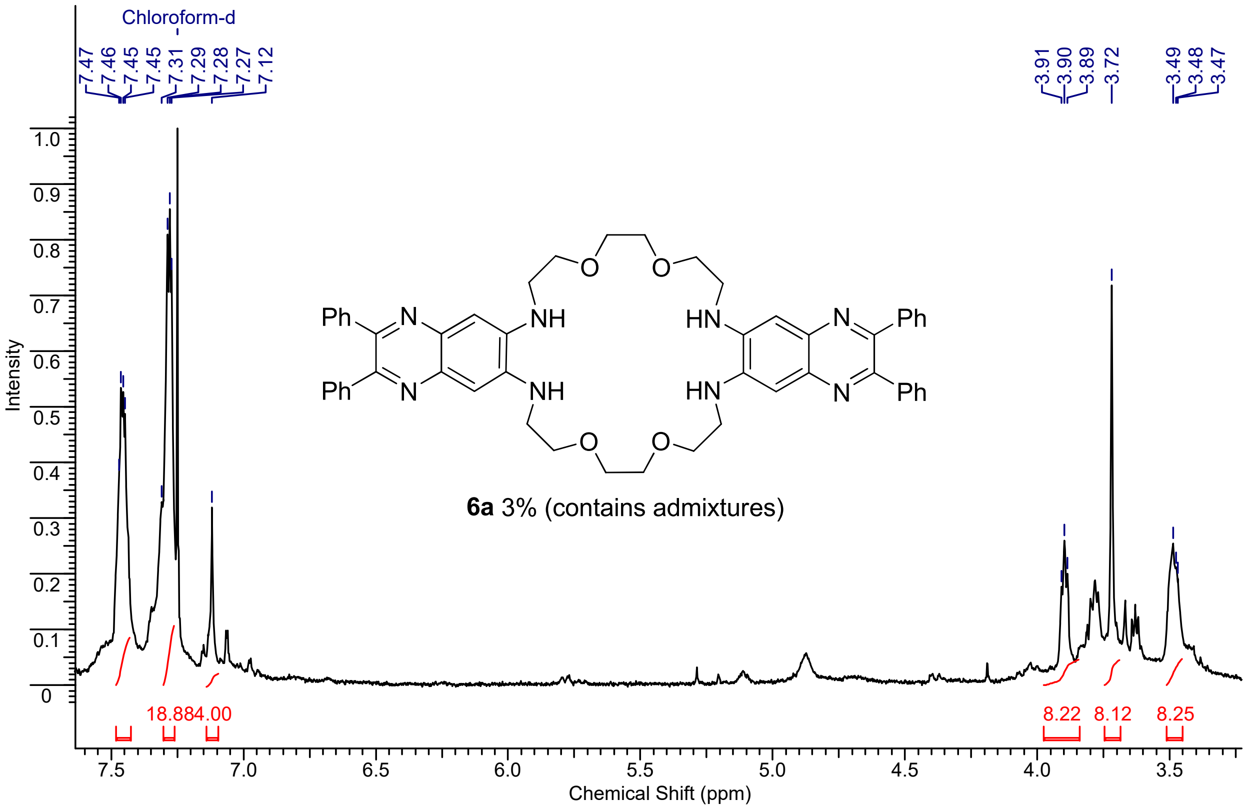

Figure S65.^{1}H NMR spectrum of the compound **6a** (contains admixtures **5a**), ($CDCl_3$, 400 MHz, 300 K).

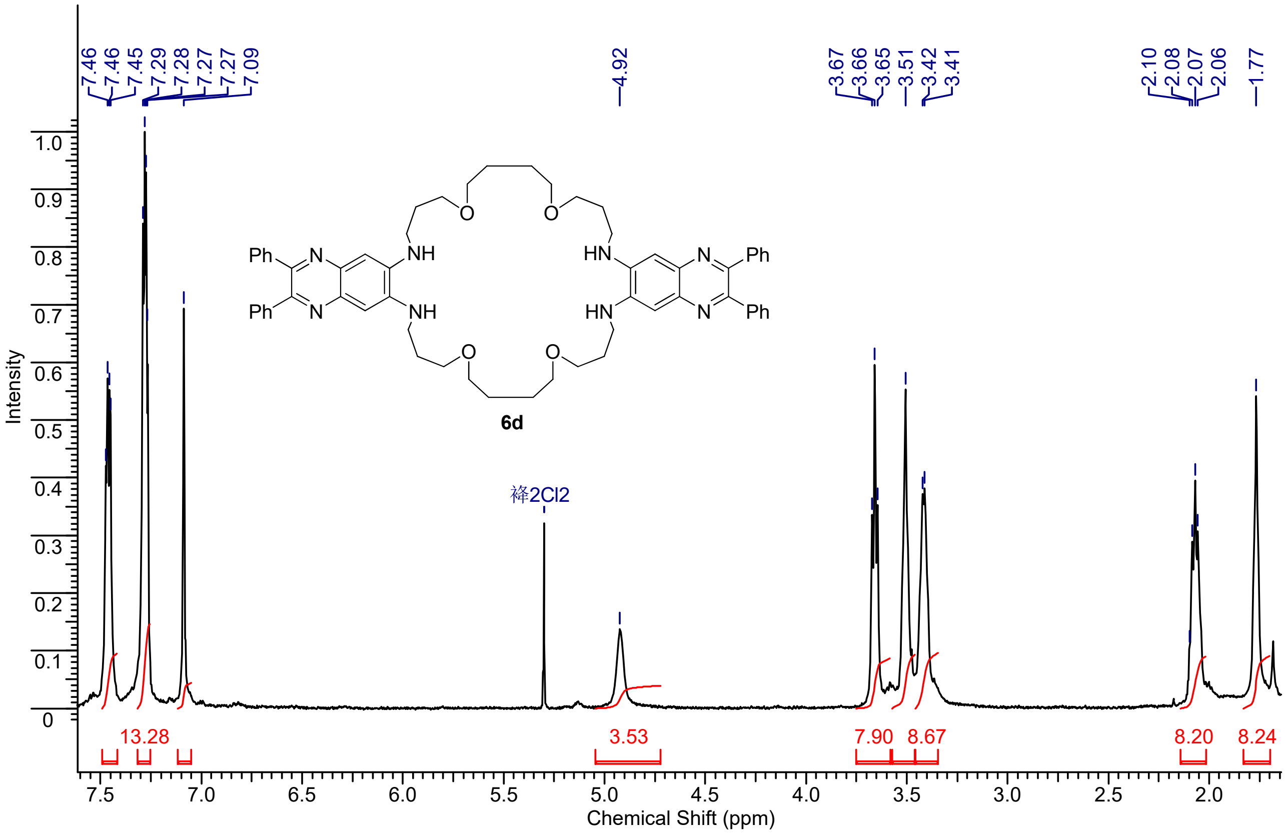

Figure S66. ^{1}H NMR spectrum of the compound **6d** ($CDCl_3$, 400 MHz, 300 K).

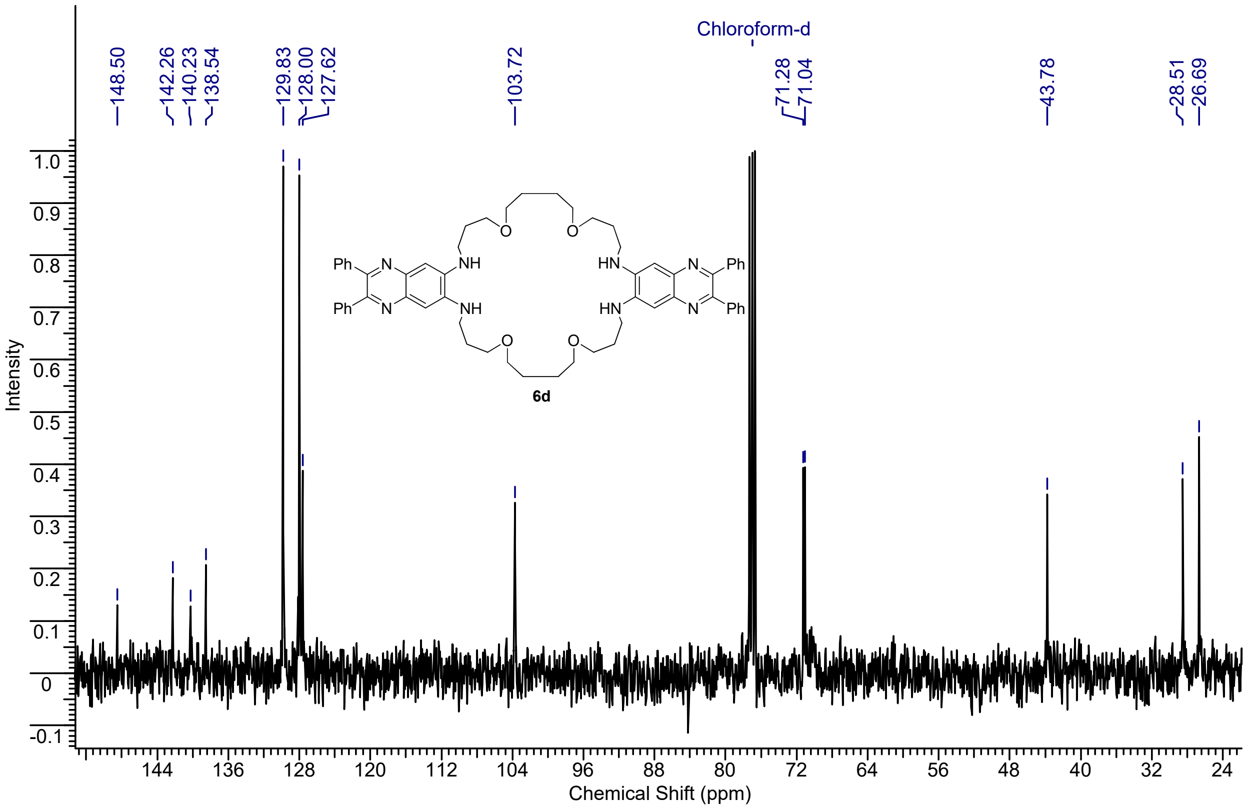

Figure S67. ^{13}C NMR spectrum of the compound **6d** ($CDCl_3$, 100.6 MHz, 300 K).

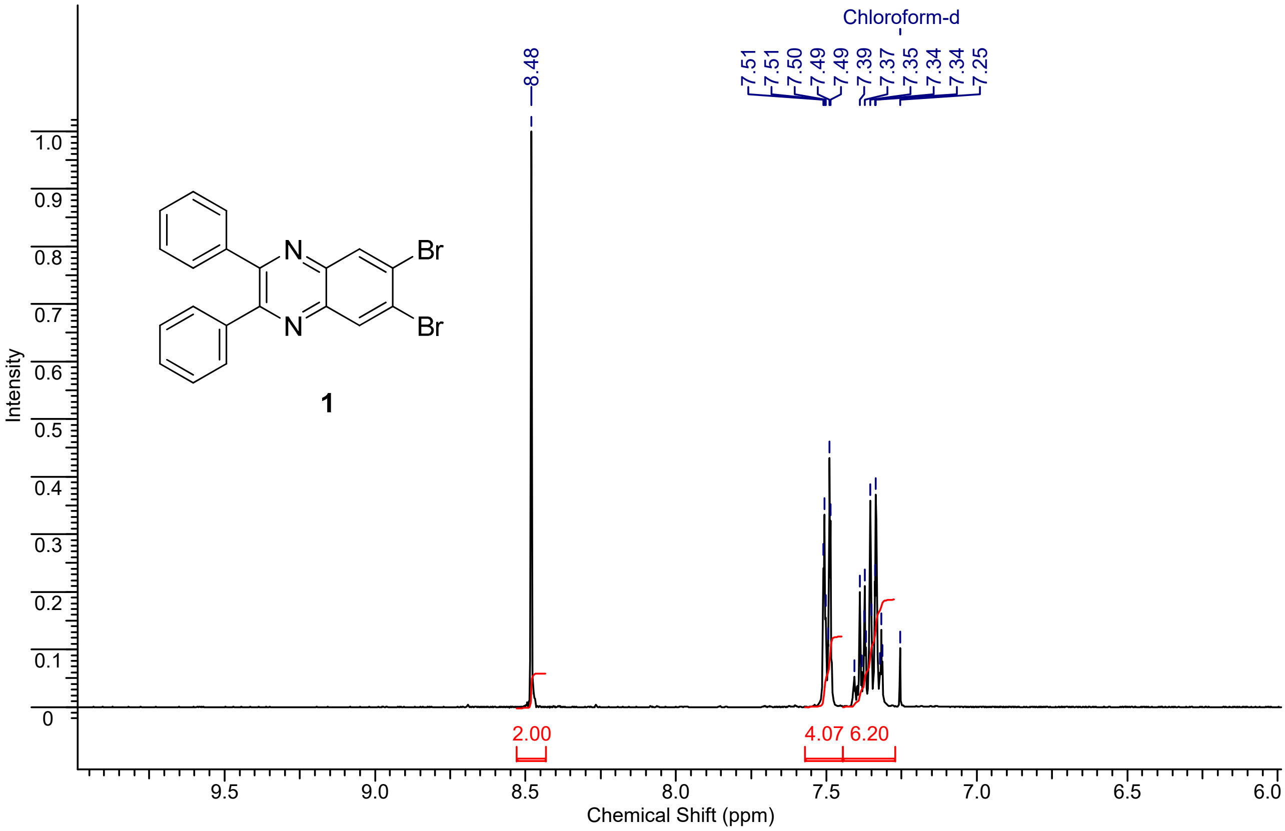

Figure S68. ^{1}H NMR spectrum of the compound **1** ($CDCl_3$, 400 MHz, 300 K).

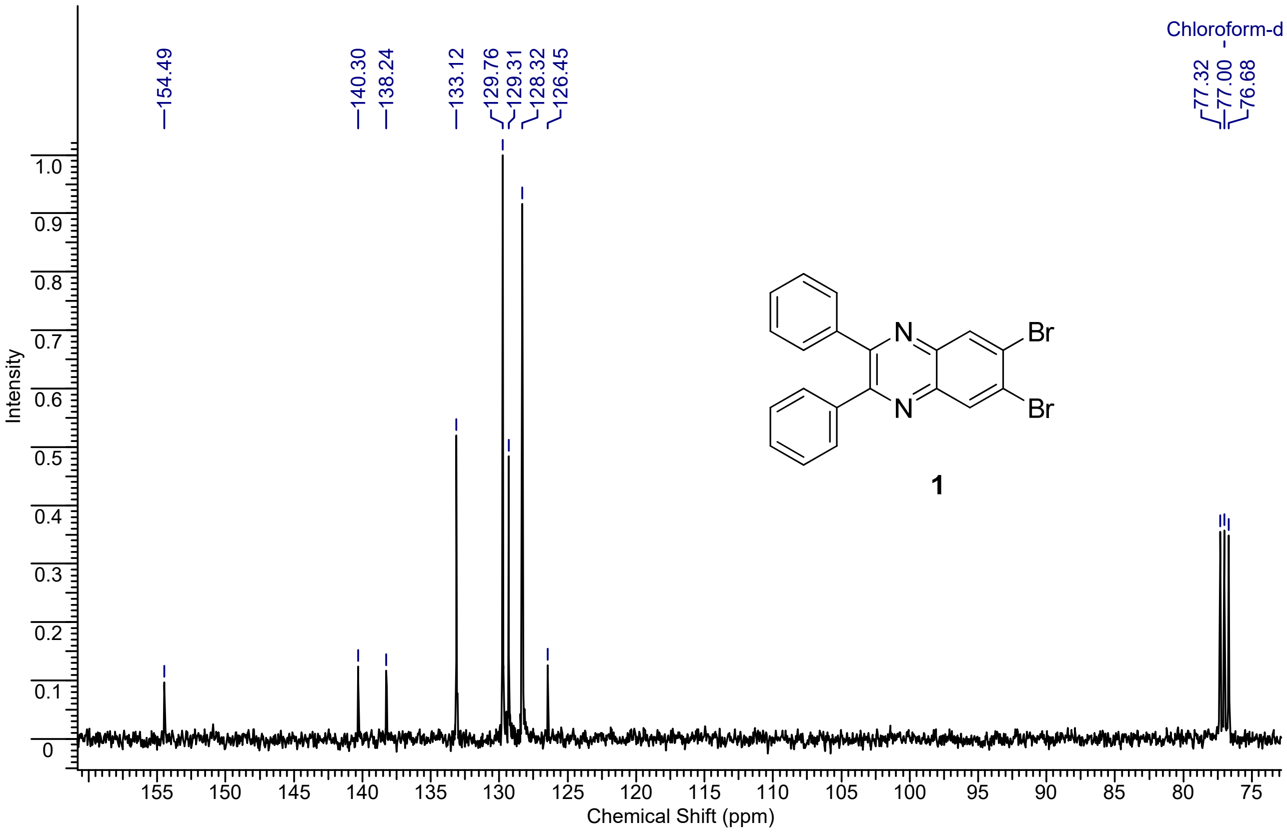

Figure S69. ^{13}C NMR spectrum of the compound **1** ($CDCl_3$, 100.6 MHz, 300 K).

6. References

1. Wong, K.-L.; Bünzli, J.-C.G.; Tanner, P.A. Quantum yield and brightness. *J. Lumin.* **2020**, *224*, 117256, doi:10.1016/j.jlumin.2020.117256.
2. Gans, P.; Sabatini, A.; Vacca, A. Investigation of equilibria in solution. Determination of equilibrium constants with the HYPERQUAD suite of programs. *Talanta* **1996**, *43*, 1739–1753, doi:10.1016/0039-9140(96)01958-3.
